# Dearomative Cyclization of Ynamides toward the Formation of Fused Diazabicycles

**DOI:** 10.1021/acs.orglett.5c02963

**Published:** 2025-09-02

**Authors:** Mohamed Agbaria, Zackaria Nairoukh

**Affiliations:** Institute of Chemistry, Casali Center of Applied Chemistry, The Hebrew University of Jerusalem, Jerusalem 9190401, Israel

## Abstract

We report a selective dearomative cyclization strategy for the synthesis of 3,4-fused diazabicycles from 3-substituted pyridyl ynamides. The method combines a chemo-, regio-, and stereoselective carbometalation with a regioselective dearomatization, enabling access to a broad range of diazabicyclic scaffolds with varied ring sizes. The protocol accommodates alkyl and aryl Grignard reagents, tolerates diverse functional groups, and supports stereodivergent synthesis, offering a versatile platform for constructing complex fused *N*-heterocycles with potential relevance to medicinal chemistry.

## Graphical abstract

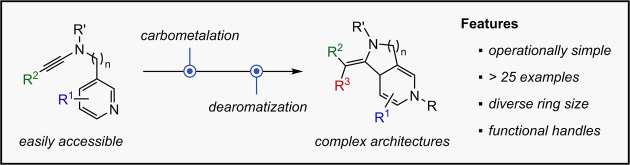

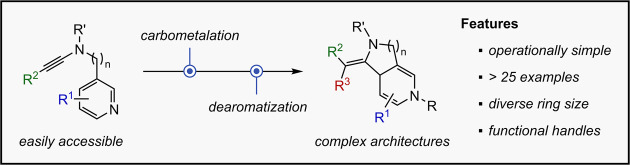

Nitrogen-containing aromatic and aliphatic heterocycles are ubiquitous in pharmaceuticals and natural products.^1^ In drug discovery, these scaffolds are frequently used to fine-tune key molecular properties such as lipophilicity, polarity, and hydrogen-bonding capacity.^1^ Notably, successful drug candidates increasingly feature sp^3^-rich frameworks,^2^ which are associated with improved solubility and enhanced target selectivity.^1b–1d,3^

This shift toward aliphatic *N*-heterocycles has highlighted a major limitation in synthetic chemistry: the lack of efficient and selective methods for their construction.^4^ Among the most direct strategies to address this challenge is dearomative functionalization reaction,^5,6^ which enables the conversion of planar *N*-heterocycles into three-dimensional, sp^3^-rich architectures while introducing valuable functionalities in a controlled manner. Despite the energetic challenge associated with disrupting aromaticity, recent advances have delivered powerful dearomatization protocols allowing access to a previously underexplored chemical space with high levels of selectivity.^7–9^

We have a longstanding interest in developing dearomative functionalization strategies for the synthesis of aliphatic *N*-heterocycles.^10^ More recently, we systematically explored the chemical space of aza-spiropiperidyl compounds by designing a selective dearomative spirocyclization reaction (Scheme 1).^11^ In this approach, 4-substituted pyridyl ynamides **1** underwent a chemo-, regio-, and stereoselective carbometalation event to form (*Z*)-vinyl metal intermediates. These reactive species subsequently engaged in an intramolecular, regioselective nucleophilic dearomatization reaction at the C4 position, furnishing diverse aza-spiro piperidine scaffolds **2** with multiple functional handles. This strategy was further extended to access diazaspiro heterocycles with varying ring sizes, underscoring its synthetic utility and structural versatility.^11^

**Scheme 1:**
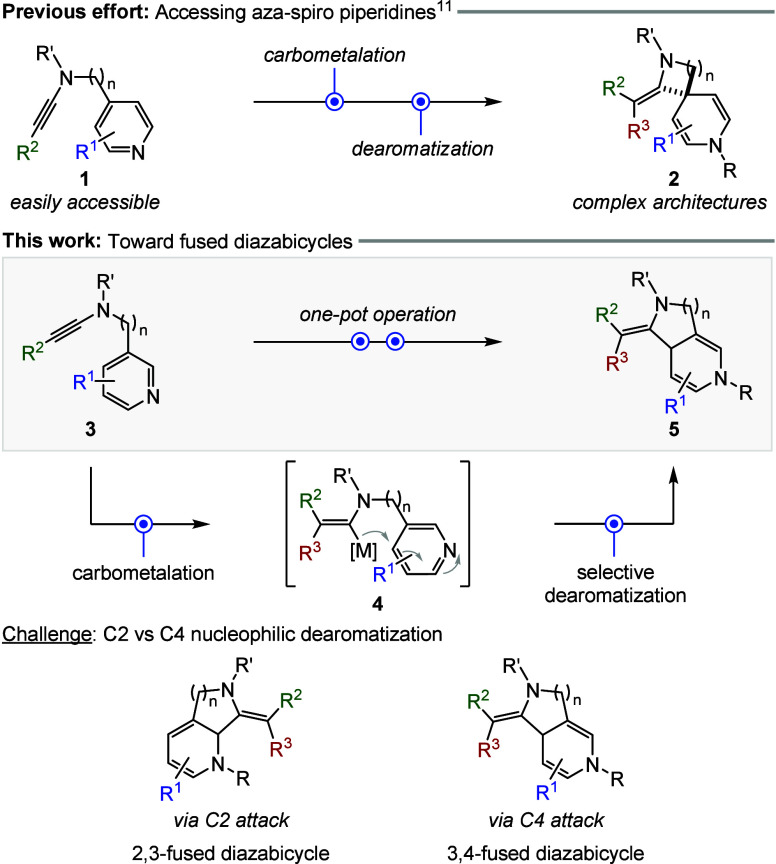
Dearomative Cyclization of Ynamides

In light of the above, we set out to explore the formation of fused diazabicyclic scaffolds using a related strategy (Scheme 1). To realize the desired structures, we envisioned that 3-substituted pyridyl ynamides **3** could undergo a chemo-, regio-, and stereoselective carbometalation to generate vinyl metal intermediates **4**. However, the subsequent nucleophilic dearomatization posed a significant regioselectivity challenge, as it could proceed via either C2 or C4 attack, leading to the formation of 2,3- or 3,4-fused diazabicycles, respectively. Achieving high regioselectivity in this context would therefore require careful fine-tuning of the metalated nucleophile.^12^

Herein, we present a dearomative cyclization strategy of ynamides that enables efficient access to structurally diverse 3,4-fused diazabicyclic scaffolds **5** in a chemo-, regio-, and stereoselective manner (Scheme 1).^13^ These rigid frameworks could serve as versatile building blocks for downstream functionalization, facilitating the construction of aliphatic, highly functionalized, fused *N*-heterocycles.

A library of 3-substituted pyridyl ynamides **3** was efficiently synthesized via C–N coupling reactions between bromoalkynes and suitable aminopyridine derivatives,^14,15^ as detailed in the Supporting Information.^16^ With these substrates in hand, we initiated our investigation by optimizing the carbometalation step, building on our previous findings^11^ and inspired by seminal reports on the carbocupration of ynamides (Table 1).^17,18^ Gratifyingly, the copper-catalyzed carbomagnesiation of ynamide **3a** with EtMgBr (2.0 equiv) in dichloromethane, followed by quenching with methanol, delivered (*Z*)-vinylpyridin-3-ylmethanamine **4a** as a single regio- and stereoisomer in 64% yield (entry 4). Note that the stereochemistry of the intermediate was confirmed by NMR spectroscopy.^16^ Reducing the amount of Grignard reagent to 1.5 and 1.1 equiv further improved the yield to 74% and 82%, respectively (entries 1 and 3).

**Table 1:**
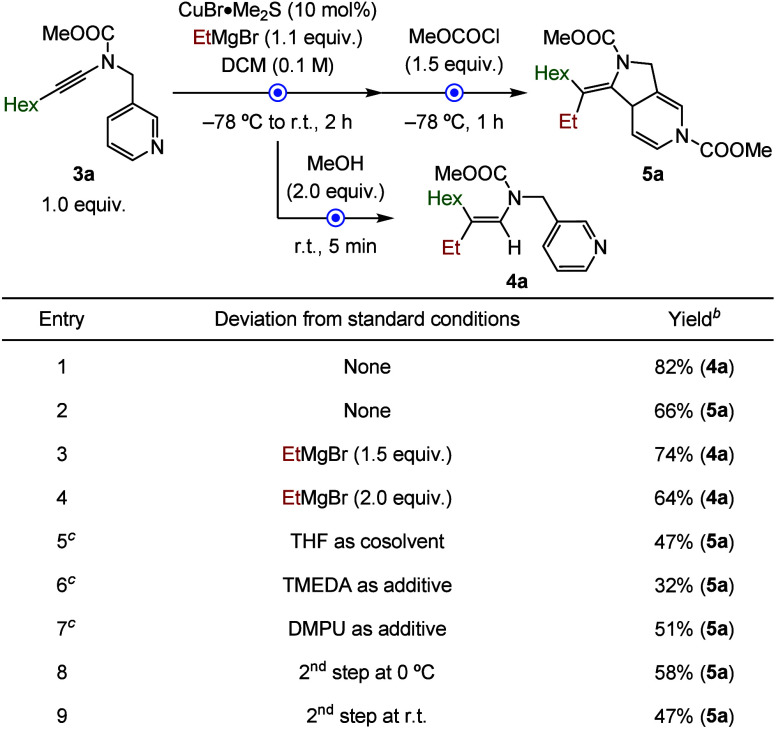
Effect of Reaction Parameters^a^

a Reactions were performed under N_2_ with 0.2 mmol of **3a**.

b Yields were determined after column chromatography.

c The additive was added in the second step prior to the addition of the Lewis acid. See Supporting Information for more details on optimization studies. Et, ethyl; Hex, hexyl; THF, tetrahydrofuran; TMEDA, tetramethylethylenediamine; DMPU, *N*,*N*′-dimethylpropyleneurea.

With the first step optimized, the subsequent cyclization was facilitated by the addition of methyl chloroformate at −78 °C (Table 1). The desired 3,4-fused diazabicyclic scaffold **5a** was obtained in 66%, with regioselectivity attributed to the soft character of the vinyl metal intermediate (entry 2).^12^ Attempts to improve the efficiency of this step by introducing cosolvents (e.g., THF) or metal scavengers such as TMEDA or DMPU resulted in reduced overall yields (entries 5, 6, and 7). Moreover, performing the cyclization at elevated temperatures negatively affected the reaction outcome (entries 8 and 9).

Building on the optimized low-temperature conditions, we next explored the substrate scope of the transformation (Scheme 2). Using ynamide **3a** (R^2^ = Hex) as a model substate, we first demonstrated that the synthesis of diazabicyclic product **5a** could be effectively scaled without significantly compromising the yield. Next, we examined the addition of other primary alkyl Grignard reagents, including methyl (Me), butyl (Bu), and hexyl (Hex) magnesium bromides to ynamide **3a**, which furnished the corresponding fused heterocycles **5b**–**5d** in moderate to good overall yields. The use of a secondary Grignard reagent, such as *i*PrMgBr, afforded the desired product **5e** in lower yield, likely due to steric hindrance impairing the efficiency of the carbometalation step. Interestingly, a variety of aryl Grignard reagents also proved compatible, delivering the vinyl metal intermediates as single regio- and stereoisomers and furnishing the fused adducts (**5f**, **5h**–**5k**) in acceptable yields as single isomers. Among those are *p*-anisyl- and *p*-anilyl magnesium bromides, delivering the corresponding adducts **5j** and **5k** with excellent selectivity, albeit in low yields, due to reduced efficiency in the carbometalation step. Unfortunately, *o*-tolylmagnesium bromide produced only the carbometalated intermediate, and all attempts to promote the subsequent dearomatization step were unsuccessful, presumably due to increased steric congestion.

**Scheme 2:**
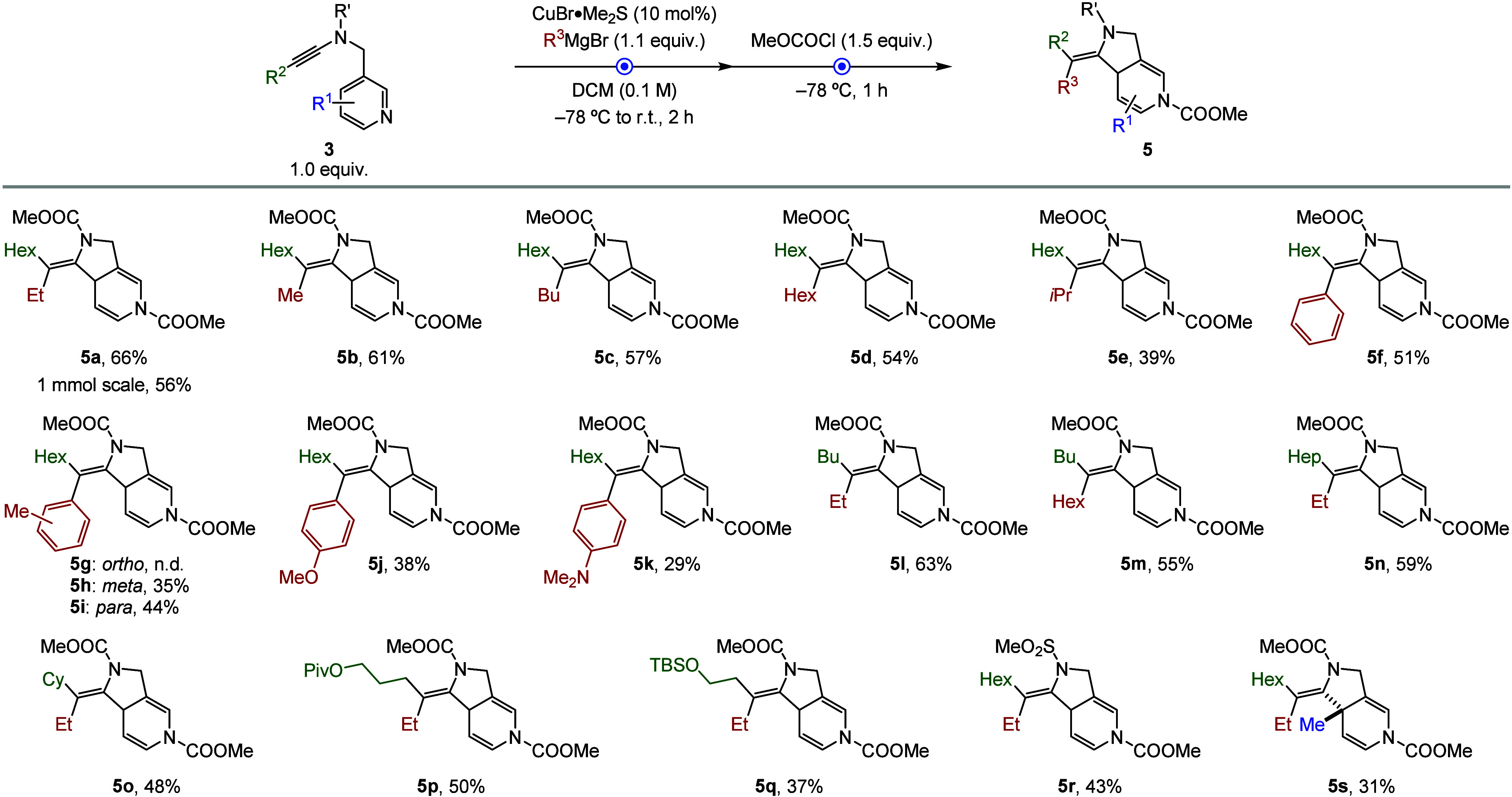
Scope of Tetrahydropyrrolopyridine Derivatives^a^

a Reactions were carried out on a 0.3 mmol scale. Yields were determined after column chromatography. For detailed experimental procedures, see Supporting Information. Me, methyl; Et, ethyl; *i*Pr, *iso*-propyl; Bu, butyl; Hex, hexyl; Hep, heptyl; Cy, cyclohexyl; Piv, pivaloyl; TBS, *tert*-butyldimethylsilyl.

Having established the scope with respect to Grignard reagents, we turned our attention to the ynamide component (Scheme 2). Variation of the R^2^ substituent to butyl, heptyl, or cyclohexyl provided tetrahydropyrrolopyridines **5l**–**5o** in good overall yields. Interestingly, exchanging the substituents on the alkyne and the Grignard reagent allowed access to the opposite isomer **5m** in 55% yield, still as a single isomer. This result highlights the stereodivergent potential of the one-pot carbometalation–dearomatization sequence and its flexibility for accessing complementary isomers from readily available starting materials.

Functional group tolerance was further demonstrated with ynamides bearing pivaloyl (Piv)- and *tert*-butyldimethylsilyl (TBS)-protected alcohols, which smoothly furnished the corresponding fused heterocycles with excellent selectivity. In addition, replacing the carbamate with methanesulfonamide group produced compound **5r** in good overall yield, indicating that the transformation tolerates a diverse nitrogen-based protecting groups. To further highlight the synthetic utility of this method, substituted pyridines were incorporated. This enabled the formation of compound **5s** in moderate yield, featuring a quaternary stereocenter, thus demonstrating the potential of this strategy for accessing highly decorated piperidine frameworks. While other ynamides bearing substituted pyridines are expected to be compatible with the reaction conditions, the scope explored here was limited by their synthetic accessibility.^14,15^

The effect of different Lewis acids on the dearomatization step was systematically evaluated (Scheme 3). Overall, alkyl and aryl chloroformates proved to be the most effective activators, delivering the desired diazabicyclic products (**5t**, **5u**, and **5v**) with higher efficiency. In contrast, other electrophilic activators, including acyl chlorides and trifluoroacetic anhydride, failed to promote the dearomatization under the standard conditions.

**Scheme 3:**
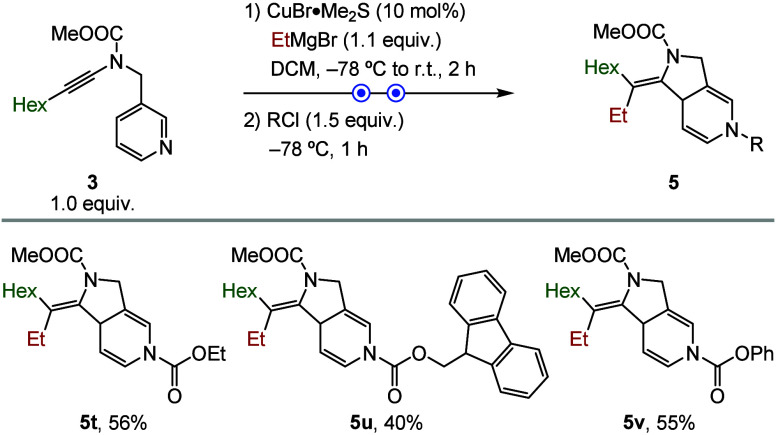
Evaluation of Chloroformate Activators^a^

a Reactions were carried out on a 0.3 mmol scale. Yields were determined after column chromatography. For detailed experimental procedures, see Supporting Information.

Having completed comprehensive studies on the formation of tetrahydropyrrolopyridine derivatives, we next turned our attention to ynamides bearing extended linkers (*n* = 2) to access hexahydronaphthyridine derivatives. Suitable substrates were prepared according to literature procedures.^17,18^ Pleasingly, copper-catalyzed carbomagnesiation of ynamide **6a** (R^2^ = Hex) with EtMgBr proceeded smoothly to afford the desired product **7a** in good overall yield and as a single isomer (Scheme 4). Replacing ethyl with butyl, hexyl, or *p*-anilyl Grignard reagents provided the corresponding adducts **7b**–**7d** in acceptable yields. Additional ynamides, including substrate bearing a TBS-protected alcohol, furnished functionalized product **7e** with excellent selectivity, albeit in modest yields.

**Scheme 4:**
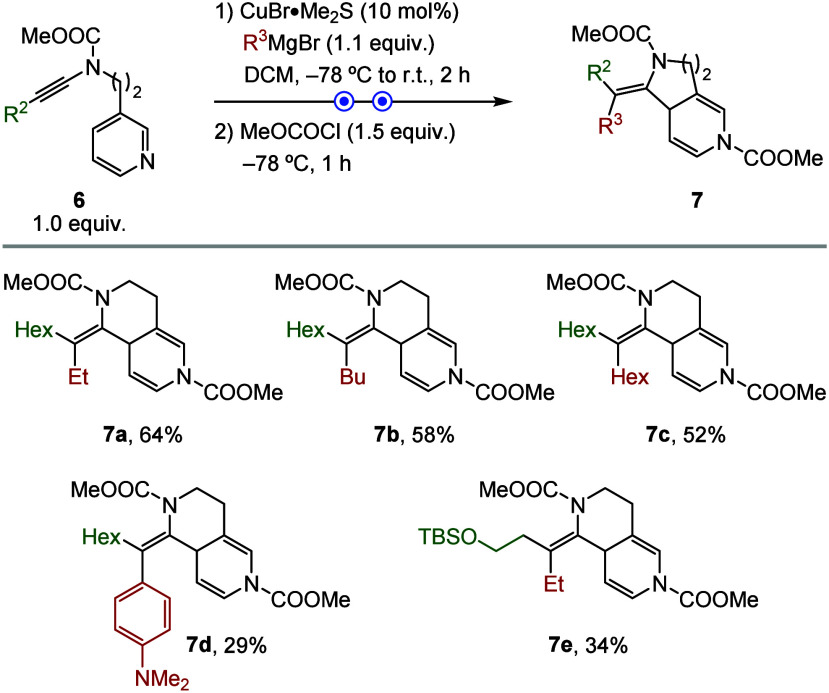
Scope of Hexahydronaphthyridine Derivatives^a^

a Reactions were carried out on a 0.3 mmol scale. Yields were determined after column chromatography. For detailed experimental procedures, see Supporting Information.

Postfunctionalization of the 3,4-fused *N*-heterocycles further highlighted the versatility of this method (Scheme 5). In particular, the hydrogenation of both tetrahydropyrrolopyridine **5a** and hexahydronaphthyridine **7a** could be precisely controlled. Under partial reduction conditions using catalytic Pd/C, the corresponding fused piperidines **8a** and **9a** were selectively obtained, while preserving the tetrasubstituted alkene moiety, a valuable functional handle for subsequent derivatization.

**Scheme 5:**
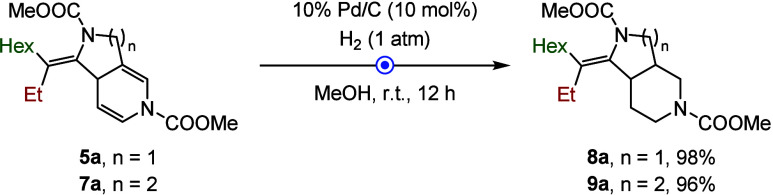
Partial Hydrogenation of 3,4-Fused Diazabicycles^a^

a Yields were determined after column chromatography. For detailed experimental procedure, see Supporting Information.

In conclusion, we have developed a stereoselective approach to 3,4-fused diazabicycles through a dearomative cyclization sequence. The approach combines a chemo-, regio-, and stereoselective carbometalation reaction with a regioselective dearomatization step. Its modular design enables access to fused *N*-heterocycles featuring diverse ring sizes and functional groups, offering a powerful platform for the synthesis of structurally complex scaffolds.

## Supplementary Material



## Data Availability

The data underlying this study are available in the published article and its Supporting Information.

## Notes

Experimental procedures, extensive optimization data, and comprehensive NMR analysis and MS data for all new compounds (PDF)

## References

[1] O’Hagan D. (2000). Pyrrole, Pyrrolidine,
Pyridine, Piperidine
and Tropane Alkaloids. Nat. Prod. Rep..

[2] Lovering F., Bikker J., Humblet C. (2009). Escape from Flatland:
Increasing Saturation as an Approach to Improving Clinical Success. J. Med. Chem..

[3] Ishikawa M., Hashimoto Y. (2011). Improvement
in Aqueous Solubility
in Small Molecule Drug Discovery Programs by Disruption of Molecular
Planarity and Symmetry. J. Med. Chem..

[4] Blakemore D. C., Castro L., Churcher I., Rees D. C., Thomas A. W., Wilson D. M., Wood A. (2018). Organic Synthesis Provides
Opportunities to Transform Drug Discovery. Nat.
Chem..

[5] Huck C. J., Sarlah D. (2020). Shaping Molecular Landscapes: Recent
Advances, Opportunities,
and Challenges in Dearomatization. Chem..

[6] Roche S. P., Porco Jr J. A. (2011). Dearomatization Strategies in the
Synthesis of Complex Natural Products. Angew.
Chem., Int. Ed..

[7] Fernández-Ibáñez M. Á., Maciá B., Pizzuti M. G., Minnaard A. J., Feringa B. L. (2009). Catalytic
Enantioselective Addition of Dialkylzinc Reagents to *N*-Acylpyridinium Salts. Angew. Chem., Int. Ed..

[8] Grozavu A., Hepburn H. B., Smith P. J., Potukuchi H. K., Lindsay-Scott P. J., Donohoe T. J. (2019). The Reductive C3
Functionalization
of Pyridinium and Quinolinium Salts through Iridium-Catalysed Interrupted
Transfer Hydrogenation. Nat. Chem..

[9] Zhang Y.-Q., Chen Y.-B., Liu J.-R., Wu S.-Q., Fan X.-Y., Zhang Z.-X., Hong X., Ye L.-W. (2021). Asymmetric Dearomatization
Catalysed by Chiral Brønsted Acids via Activation of Ynamides. Nat. Chem..

[10] Hu M., Ding D., Desnoo W., Tantillo D. J., Nairoukh Z. (2023). The Construction of Highly Substituted
Piperidines via Dearomative Functionalization Reaction. Angew. Chem., Int. Ed..

[11] Agbaria M., Egbaria N., Nairoukh Z. (2024). Dearomative Spirocyclization of Ynamides. Chem. Sci..

[12] Bull J. A., Mousseau J. J., Pelletier G., Charette A. B. (2012). Synthesis of Pyridine
and Dihydropyridine Derivatives by Regio- and Stereoselective Addition
to *N*-Activated Pyridines. Chem.
Rev..

[13] Agbaria M., Nairoukh Z. (2025). Dearomative Cyclization
of Ynamides towards the Formation
of Fused Diazabicycles. ChemRxiv.

[14] Yao P.-Y., Zhang Y., Hsung R. P., Zhao K. (2008). A Sequential Metal-Catalyzed C-N Bond Formation in the Synthesis
of 2-Amido-indoles. Org. Lett..

[15] DeKorver K.
A., Li H., Lohse A. G., Hayashi R., Lu Z., Zhang Y., Hsung R. P. (2010). Ynamides:
A Modern Functional Group for the New Millennium. Chem. Rev..

[16] See the Supporting Information for more details.

[17] Basheer A., Marek I. (2010). Recent Advances in Carbocupration
of α-Heterosubstituted Alkynes. Beilstein,
J. Org. Chem..

[18] Minko Y., Pasco M., Lercher L., Botoshansky M., Marek I. (2012). Forming All-Carbon Quaternary Stereogenic Centres in Acyclic Systems
from Alkynes. Nature.

