# Supplementary material for: Dearomative Cyclization of Ynamides toward the Formation of Fused Diazabicycles
Source: Org Lett. 2025 Sep 2;27(36):9982–6. doi: 10.1021/acs.orglett.5c02963 (PMC12442226; doi:10.1021/acs.orglett.5c02963)

Dearomative Cyclization of Ynamides towards the Formation of Fused Diazabicycles

Mohamed Agbaria and Zackaria Nairoukh*

Institute of Chemistry, Casali Center of Applied Chemistry, The Hebrew University of Jerusalem, Jerusalem 9190401, Israel

Correspondence to: z.nairoukh@mail.huji.ac.il

Supporting Information

Table of Contents

1. Materials and Methods... 2

2. Preparation of Starting Materials…... 3

3. Reaction Optimization 9

   3.1. Carbometalation reaction...9

   3.2. Carbometalation-dearomatization reaction……………………..……..……12

4. Diazabicycles Products ……………..…………………………………...........................15

5. Application 45

6. References..48

7. NMR Spectra... 49

1. Materials and Methods

All reactions were carried out in a flame-dried glassware under positive pressure of nitrogen in dry solvents using standard Schlenk techniques unless otherwise indicated.Thin layer chromatography was performed on TLC Silica gel 60 F254 plates. Visualization was accomplished with short wave UV light, and/or iodine, *p*-anisaldehyde, $KMnO_4$, phosphomolybdic acid (PMA), and Hanessian's (cerium ammonium molybdate) stains. Flash chromatography was performed on Apollo Scientific silica gel (40–63 mesh) by standard technique eluting with solvents as indicated.

^1H and ^{13}C spectra were recorded on a Bruker Avance II 400 or Avance II 500 in the indicated solvents. Chemical shifts (δ) are given in ppm relative to TMS. The residual solvent signals were used as references and the chemical shifts converted to the TMS scale ($CDCl_3$: $\delta_H = 7.26$ ppm, $\delta_C = 77.16$ ppm). High resolution mass spectrometry (HRMS) spectra were obtained on a Bruker miorOTOF-QII instrument, Bruker maXis impact II, and Thermo Fisher Scientific Dionex UltiMate 3000 UPLC-Q Exactive Plus.

Tetrahydrofuran, diethyl ether, toluene and dichloromethane (HPLC grade, non-stabilized, BioLab) were dried using Innovative Technology PureSolv PS-MD-2 solvent purifier (aluminum oxide columns) and kept under positive pressure of nitrogen (99.9999% purity grade).Methylmagnesium bromide solution in diethyl ether (3.0 M) was purchased from Aldrich and used as received.Other Grignard reagents were prepared according to standard procedure and titrated before use.[1-2]All organometallic compounds, dry solvents and reagents were transferred using plastic single use graduated syringes and oven dried stainless-steel needles. Copper (I) bromide – dimethylsulfide complex, copper(I) iodide, amines, alkynes, and other commercially available chemicals were obtained from BLD pharm, Across Organics, Aldrich Chemical Co., and Alfa Assar.

2. Preparation of Starting Materials

All ynamides were prepared on a multi-gram scale according to a literature procedure for copper-catalyzed C–N bond formation between bromoalkynes and protected 3-(aminoalkyl)pyridines.[3-5] All bromoalkynes were prepared according to a previously published procedure.[3]

Methyl (pyridin-3-ylmethyl)carbamate

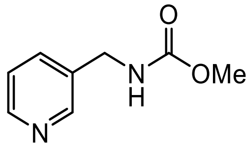

^{1}H NMR (400 MHz, $CDCl_3$) δ 8.52 (bs, 2H), 7.62 (d, *J* = 5.4 Hz, 1H), 7.29 – 7.20 (m, 1H), 5.32 (s, 1H), 4.36 (s, 2H), 3.68 (s, 3H); **^{13}C NMR** (101 MHz, $CDCl_3$) δ 157.7, 149.9, 149.1, 135.4, 134.1, 123.6, 52.4, 42.7.

***N*-(Pyridin-3-ylmethyl)methanesulfonamide**

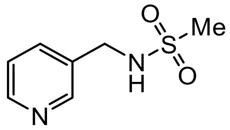

^{1}H NMR (400 MHz, $CDCl_3$) δ 8.52 (s, 1H), 8.50 (d, *J* = 4.6 Hz, 1H), 7.68 (d, *J* = 7.9 Hz, 1H), 7.26 (dd, *J* = 7.6, 4.8 Hz, 1H), 5.22 (s, 1H), 4.29 (s, 2H), 2.87 (s, 3H); **^{13}C NMR** (101 MHz, $CDCl_3$) δ 149.5, 149.2, 135.0, 123.8, 46.5, 44.5, 41.8.

Methyl ((4-methylpyridin-3-yl)methyl)carbamate

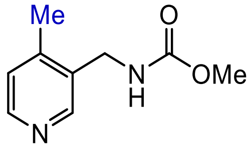

^{1}H NMR (400 MHz, $CDCl_3$) δ 8.27 (s, 1H), 8.25 (d, *J* = 4.9 Hz, 1H), 6.94 (d, *J* = 5.0 Hz, 1H), 4.98 (s, 1H), 4.24 (d, *J* = 5.4 Hz, 2H), 3.55 (t, *J* = 0.9 Hz, 3H), 2.20 (s, 3H); **^{13}C NMR** (101 MHz, $CDCl_3$) δ 155.3, 149.3, 146.3, 145.7, 131.9, 125.3, 53.8, 40.8, 27.7.

Methyl (2-(pyridin-3-yl)ethyl)carbamate

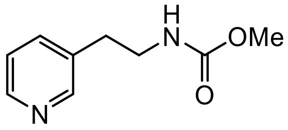

^{1}H NMR (400 MHz, $CDCl_3$) δ 8.50 – 8.33 (m, 2H), 7.53– 7.48 (m, 1H), 7.24 – 7.18 (m, 1H), 4.88 (s, 1H), 3.64 (s, 3H), 3.48 – 3.35 (m, 2H), 2.83 – 2.77 (m, 2H); **^{13}C NMR** (101 MHz, $CDCl_3$) δ 156.9, 150.1, 148.0, 136.2, 134.2, 123.4, 52.1, 41.9, 33.4.

Preparation of Ynamides

The preparation of the following ynamides was carried out according to the literature[5] as follows:

Methyl oct-1-yn-1-yl(pyridin-3-ylmethyl)carbamate (3a)

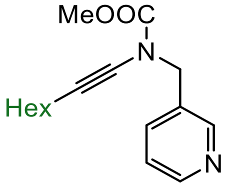

^{1}H NMR (400 MHz, $CDCl_3$) δ 8.61 (s, 1H), 8.57 (dd, J = 4.8, 1.4 Hz, 1H), 7.73 – 7.66 (m, 1H), 7.30 – 7.27 (m, 1H), 4.77 (s, 2H), 3.83 (s, 3H), 2.21 (q, J = 7.1 Hz, 2H), 1.49 – 1.40 (m, 2H), 1.34 – 1.13 (m, 6H), 0.78 (t, J = 7.5 Hz, 3H); **^{13}C NMR** (126 MHz, $CDCl_3$) δ 156.2, 150.0, 149.5, 136.2, 131.9, 123.5, 73.3, 71.3, 54.2, 51.5, 31.4, 28.9, 28.5, 22.6, 18.5, 14.1.

HRMS (ESI) m/z: $[M+H]^+$ Calcd for $C_{16}H_{23}N_2O_2$: 275.1754, Found: 275.1742.

IR ν = 2924 (m), 2923 (w), 2862 (w), 1728 (s), 1435 (m), 1388 (m), 1280 (s), 1126 (w), 1026 (w), 967 (s), 758 (m), 709 (m).

Methyl hex-1-yn-1-yl(pyridin-3-ylmethyl)carbamate (3l)

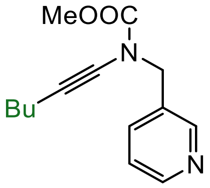

^{1}H NMR (400 MHz, $CDCl_3$) δ 8.60 – 8.49 (m, 2H), 7.67 (d, J = 7.9 Hz, 1H), 7.27 (bs, 1H), 4.58 (s, 2H), 3.78 (s, 3H), 2.21 (t, J = 6.9 Hz, 2H), 1.45 – 1.36 (m, 2H), 1.36 – 1.25 (m, 2H), 0.84 (t, J = 7.3, 3H); **^{13}C NMR** (101 MHz, $CDCl_3$) δ 156.5, 150.2, 149.8, 136.4, 132.3, 124.0, 73.6, 71.5, 54.5, 51.9, 31.3, 22.2, 18.4, 13.9.

HRMS (ESI) m/z: $[M+H]^+$ Calcd for $C_{14}H_{19}N_2O_2$: 247.1441, Found:247.1430.

IR ν = 2924 (m), 2931 (w), 2870 (w), 1720 (s), 1442 (m), 1388 (m), 1280 (s), 1234 (w), 1206, 709 (m).

Methyl non-1-yn-1-yl(pyridin-3-ylmethyl)carbamate (3n)

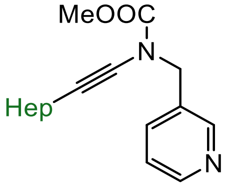

^{1}H NMR (500 MHz, $CDCl_3$) δ 8.59 (s, 1H), 8.55 (s, 1H), 7.67 (d, *J* = 7.8 Hz, 1H), 7.26 (t, *J* = 5.1 Hz, 1H), 4.59 (s, 2H), 3.79 (s, 3H), 2.21 (t, *J* = 7.1 Hz, 2H), 1.52 – 1.38 (m, 2H), 1.30 – 1.21 (m, 8H), 0.86 (t, *J* = 7.1 Hz, 3H); **^{13}C NMR** (126 MHz, $CDCl_3$) δ 156.2, 150.0, 149.5, 136.2, 131.9, 123.5, 73.3, 71.3, 54.2, 51.5, 31.8, 29.0, 28.8, 28.8, 22.7, 18.5, 14.2.

HRMS (ESI) m/z: $[M+H]^+$ Calcd for $C_{17}H_{25}N_2O_2$: 289.1910, Found: 289.1895.

IR ν = 2933 (w), 2860 (w), 1726 (s), 1438 (m), 1390 (w), 1280 (s), 1132 (w), 756 (s), 713 (m).

Methyl (cyclohexylethynyl)(pyridine-3-ylmethyl)carbamate (3o)

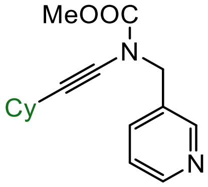

^{1}H NMR (400 MHz, $CDCl_3$) δ 8.56 (s, 1H), 8.51 (s, 1H), 7.63 (d, *J* = 7.8 Hz, 1H), 7.25 – 7.14 (m, 1H), 4.54 (s, 2H), 3.74 (s, 3H), 2.40 – 2.33 (m, 1H), 1.68 – 1.59 (m, 2H), 1.57 – 1.50 (m, 2H), 1.43 – 1.36 (m, 2H), 1.25 – 1.17 (m, 4H); **^{13}C NMR** (101 MHz, $CDCl_3$) δ 156.1, 150.1, 149.6, 136.4, 129.2, 128.3, 74.8, 72.9, 54.2, 51.6, 33.0, 32.4, 28.9, 28.0, 26.0, 24.9.

HRMS (ESI) m/z: $[M+H]^+$ Calcd for $C_{16}H_{21}N_2O_2$: 273.1597, Found: 273.1586.

IR ν = 2931 (m), 2854 (w), 1720 (s), 1442 (m), 1388 (w), 1280 (s), 1134 (w), 910 (w), 725 (s).

5-((Methoxycarbonyl)(pyridin-3-ylmethyl)amino)pent-4-yn-1-yl pivalate (**3p**)

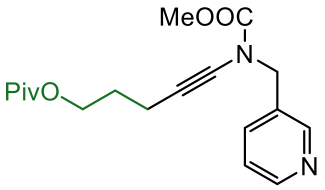

^{1}H NMR (400 MHz, $CDCl_3$) δ 8.52 (bs, 2H), 7.63 (d, *J* = 7.9 Hz, 1H), 7.25 – 7.21 (m, 1H), 4.54 (s, 2H), 4.00 (t, *J* = 6.3 Hz, 2H), 3.75 (s, 3H), 2.28 (t, *J* = 6.8 Hz, 2H), 1.80 – 1.66 (m, 2H), 1.11 (s, 9H); **^{13}C NMR** (101 MHz, $CDCl_3$) δ 178.4, 155.9, 149.8, 149.4, 136.0, 135.8, 123.5, 73.9, 69.4, 62.8, 60.3, 54.2, 38.8, 28.0, 27.2, 14.2.

HRMS (ESI) m/z: $[M+H]^+$ Calcd for $C_{18}H_{25}N_2O_4$: 333.1808, Found: 333.1797.

IR ν = 2970 (w), 2854 (w), 1720 (s), 1442 (m), 1388 (w), 1149 (s), 1034 (w), 972 (w), 709 (w).

Methyl (4-((*tert*-butyldimethylsilyl)oxy)but-1-yn-1-yl)(pyridin-3-ylmethyl)carbamate (3q)

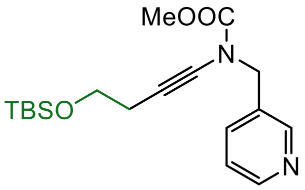

^{1}H NMR (400 MHz, $CDCl_3$) δ 8.53 (bs, 2H), 7.63 (d, *J* = 7.9 Hz, 1H), 7.24 – 7.20 (m, 1H), 4.54 (s, 2H), 3.74 (s, 3H), 3.59 (t, *J* = 7.1 Hz, 2H), 2.40 (t, *J* = 7.1 Hz, 2H), 0.81 (s, 9H), 0.00 (s, 6H);**^{13}C NMR** (101 MHz, $CDCl_3$) δ 156.1, 149.9, 149.5, 136.1, 123.6, 74.8, 68.2, 62.1, 54.2, 51.6, 26.0, 22.4, 18.4, -5.2.

HRMS (ESI)m/z: $[M+H]^+$ Calcd for $C_{18}H_{29}N_2O_3Si$: 349.1942, Found: 349.1937.

IR ν = 2970 (w), 2854 (w), 1728 (s), 1442 (m), 1388 (w), 1288 (s), 1095(s), 833 (s), 791 (s), 709 (w).

N-(oct-1-yn-1-yl)-N-(pyridin-3-ylmethyl)methanesulfonamide (3r)

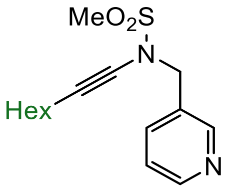

^{1}H NMR (400 MHz, $CDCl_3$) δ 8.58 (s, 1H), 8.54 (dd, J = 4.8, 1.7 Hz, 1H) ,7.73– 7.69 (m, 1H), 7.29 – 7.22 (m, 1H), 4.52 (s, 2H), 2.91 (s, 3H), 2.17 (t, J = 7.1 Hz, 2H), 1.45 – 1.30 (m, 2H), 1.28 – 1.09 (m, 6H), 0.80 (t, J = 6.87, 3H); **^{13}C NMR** (101 MHz, $CDCl_3$) δ 150.1, 150.1, 136.5, 130.7, 123.7, 72.4, 72.3, 53.0, 38.4, 31.3, 28.8, 28.5, 22.6, 18.5, 14.1.

HRMS (ESI) m/z: $[M+H]^+$ Calcd for $C_{15}H_{23}N_2O_2S$: 295.1474, Found: 295.1465.

IR ν = 2931 (w), 2854 (w), 1705 (w), 1573 (w), 1350 (s), 1157 (s), 1026 (m), 960 (m), 709 (m).

Methyl ((4-methylpyridin-3-yl)methyl)(oct-1-yn-1-yl)carbamate (3s)

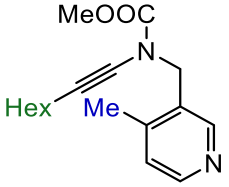

The NMR spectra contain mixture of rotamers. Only the peaks of the major rotamer are listed.

^{1}H NMR (400 MHz, $CDCl_3$) δ 8.47 (s, 1H), 8.40 (bs, 1H), 7.09 (d, J = 4.6 Hz, 1H), 4.64 (s, 2H), 3.81 (s, 3H), 2.35 (s, 3H), 2.16 (t, J = 7.0 Hz, 2H), 1.35 (q, J = 6.7 Hz, 2H), 1.24 (t, J = 7.1 Hz, 6H), 0.85 (t, J = 6.7 Hz, 3H); **^{13}C NMR** (101 MHz, $CDCl_3$) δ 156.1, 150.8, 149.5, 146.4, 130.1, 125.4, 72.4, 71.4, 60.5, 54.1, 31.4, 28.9, 28.5, 22.5, 18.9, 18.4, 14.2.

HRMS (ESI) m/z: $[M+H]^+$ Calcd for $C_{17}H_{25}N_2O_2$: 289.1910, Found: 289.1915.

IR ν = 3008 (w), 2971 (w), 2893 (w), 2206 (m), 1728 (s), 1627 (s), 1442 (m), 1342 (m), 1234 (m), 1180 (m), 972 (w), 756 (m).

Methyl oct-1-yn-1-yl(2-(pyridin-3-yl)ethyl)carbamate (6a)

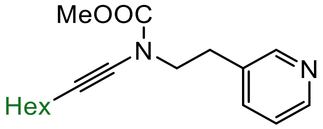

^{1}H NMR (400 MHz, $CDCl_3$) δ 8.43 (bs, 2H), 7.49 (d, *J* = 7.9 Hz, 1H), 7.16 (dd, *J* = 8.0, 4.6 Hz, 1H), 3.36 (s, 3H), 3.62 (t, *J* = 7.19 Hz, 2H), 289 (t,*J* = 7.51 Hz, 2H), 2.22 (t, *J* = 7.0 Hz, 2H), 2.21 (t,*J* = 7.12 Hz, 2H), 1.38 – 1.27 (m, 2H), 1.27 – 1.18 (m, 4H), 0.86 (t *J*= 6.61 Hz, 3H); **^{13}C NMR** (101 MHz, $CDCl_3$) δ 156.1, 150.4, 148.2, 144.6, 136.4, 123.5, 73.4, 70.9, 54.0, 50.7, 31.4, 29.8, 29.1, 28.7, 22.7, 18.6, 14.2.

HRMS (ESI) m/z: $[M+H]^+$ Calcd for $C_{17}H_{25}N_2O_2$: 289.1910, Found: 289.1896.

IR ν = 2931 (m), 2854 (w), 1728 (s), 1450 (m), 1342 (m), 1288 (s), 1203 (w), 763 (w), 709 (w).

Methyl (4-((*tert*-butyldimethylsilyl)oxy)but-1-yn-1-yl)(2-(pyridin-3-yl)ethyl)carbamate (6e)

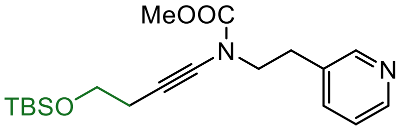

^{1}H NMR (400 MHz, $CDCl_3$) δ 8.45 (s, 1H), 8.40 (d, *J* = 1.6 Hz, 1H), 7.49 (d, *J* = 7.8 Hz, 1H), 7.20 – 7.12 (m, 1H), 3.68 (s, 3H), 3.64 (q, *J* = 6.6, 4H), 2.90 (t, *J* = 6.8 Hz, 2H), 2.45 (t, *J* = 7.1 Hz, 2H), 0.84 (s, 9H), 0.01 (s, 6H); **^{13}C NMR** (101 MHz, $CDCl_3$) δ 156.1, 150.4, 148.2, 136.5, 133.7, 123.5, 72.1, 67.8, 62.3, 60.0, 54.0 , 26.0, 23.0, 18.5, -5.1.

HRMS (ESI) m/z: $[M+H]^+$ Calcd for $C_{19}H_{31}N_2O_3Si$: 363.2098, Found: 363.2087.

IR ν **=** 2956 (w), 2868 (w), 1730 (m), 1462 (m), 1294 (w), 1259 (w), 1109 (w), 840 (w), 760 (s).

3. Reaction Optimization

3.1 Carbometalation reaction[6]

General procedure:

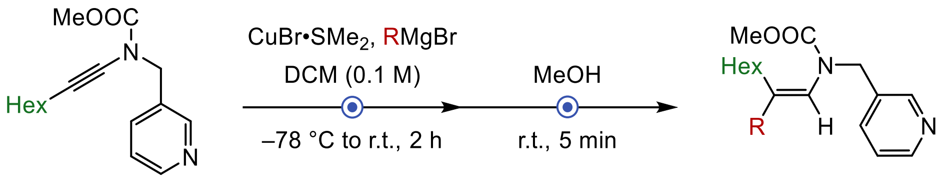

3a 1.0 equiv. **4a**

An oven-dried reaction vessel (10 mL screw-cap vial) equipped with a stirring bar was allowed to cool to room temperature under vacuum. CuBr•SMe_2 (10% mmol) and methyl oct-1-yn-1-yl(pyridin-3-ylmethyl)carbamate **3a** (55 mg, 0.2 mmol) were added under air. The vessel was then depressurized and pressurized with nitrogen three times, followed by addition of dry DCM (0.1 M, 2.0 ml). The solution was cooled to -78 °C using dry ice/acetone bath. Then, a solution of EtMgBr (2.0 equiv., in diethyl ether) was added dropwise at -78 °C. The resulting solution was stirred for 5 min then allowed to warm to room temperature. Stirring continued for 2 h at the indicated temperature (monitored by TLC on silica gel). After quenching the reaction by the addition of MeOH, the solution was concentrated under reduced pressure. Further purification of the crude via column chromatography (0-40% ethyl acetate in hexane) afforded the desired product **4a**.

The following products were isolated using the optimized reaction conditions:

Methyl (*Z*)-(2-ethyloct-1-en-1-yl)(pyridin-3-ylmethyl)carbamate (4a)

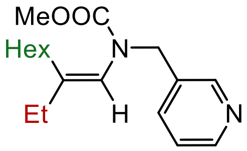

Following the general procedure on 0.2 mmol scale. The product was isolated as a yellow oil (50 mg, 0.16 mmol, 82%, *single isomer*).

Broadening in NMR spectra was observed due to the rotameric behavior of the N–COOMe group which significantly affected ^{1}H and ^{13}C NMR spectra.

^{1}H NMR (400 MHz, $CDCl_3$) δ 7.80 (bs, 2H), 5.62 (bs, 1H), 4.54 (s, 2H), 3.70 (s, 3H), 2.02 (d, *J* = 7.1 Hz, 2H), 1.87 (bs, 2H), 1.31 – 1.15 (m, 8H), 0.98 (t, *J* = 7.4 Hz, 3H), 0.87 (t, *J* = 7.0 Hz, 3H); **^{13}C NMR** (126 MHz, $CDCl_3$) δ 53.1, 31.7, 29.6, 26.9, 26.2, 22.7, 15.6, 14.2, 13.9, 12.8, 11.0.

HRMS (ESI) m/z: $[M+H]^+$ Calcd for $C_{18}H_{29}N_2O_2$: 305.2222, Found: 305.2209.

IR ν = 2924 (m), 2854 (m), 1697 (s), 1450 (m), 1377 (m), 1219 (m), 756 (s).

Methyl (*E*)-(2-phenyloct-1-en-1-yl)(pyridin-3-ylmethyl)carbamate (4f)

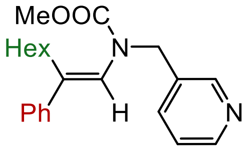

Following the general procedure on 0.2 mmol scale. The product was isolated as a yellow oil (52 mg, 0.15 mmol, 74%, *single isomer*).

Broadening in NMR spectra was observed due to the rotameric behavior of the N–COOMe group which significantly affected ^{1}H and ^{13}C NMR spectra.

^{1}H NMR (400 MHz, $CDCl_3$) δ 7.92 (s, 1H), 7.29 (d, *J* = 8.2 Hz, 4H), 6.03 (bs, 1H), 4.80 (s, 2H), 3.75 (s, 3H), 2.33 (s, 2H), 1.90 – 1.66 (m, 1H), 1.27 – 1.07 (m, 8H), 0.83 (t, *J* = 6.9 Hz, 3H); **^{13}C NMR** (126 MHz, $CDCl_3$) δ 139.4, 130.6, 128.5, 127.8, 127.0, 53.3, 31.7, 29.6, 27.3, 22.7, 14.1.

HRMS (ESI) m/z: $[M+H]^+$ Calcd for $C_{22}H_{29}N_2O_2$: 353.2222, Found: 353.2210.

IR ν = 2974 (w), 2935 (w), 1695 (m), 1446 (w), 1278 (w), 1055 (s), 1018 (s), 763 (w) 700 (w).

Table S1. Evaluation of reaction Grignard reagent.

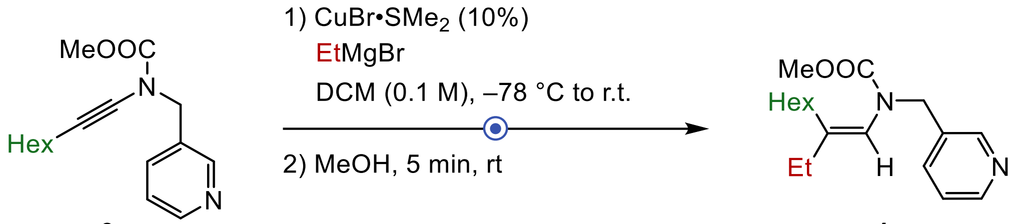

Entry	EtMgBr (equiv)	Yield (%)
**1**	**1.1**	**82**
2	1.5	74
3	2	64

Reactions were performed under N_2 with 0.2 mmol of **3a**. The reported yields were determined after column chromatography.

3.2 Carbometalation-dearomatization reaction

General procedure:

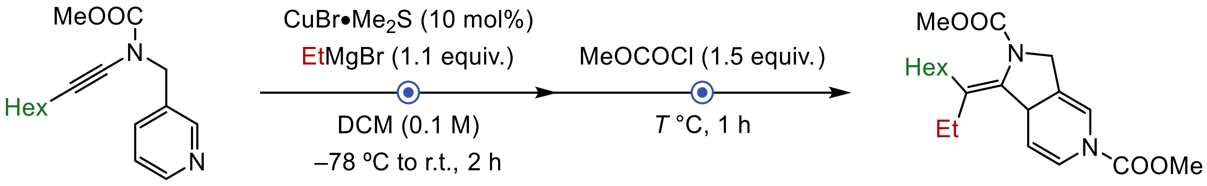

3a 1.0 equiv. **5a**

An oven dried Schlenk flask equipped with a stirring bar was allowed to cool to room temperature under vacuum. CuBr•SMe_2 (10% mmol), and methyl oct-1-yn-1-yl(pyridin-3-ylmethyl)carbamate **3a** (55.0 mg , 0.2 mmol, 1.0 equiv.) were added under air. The vessel was then depressurized and pressurized with nitrogen three times, followed by addition of dry DCM (0.1 M, 2.0 ml). The solution was cooled to -78 °C using dry ice/acetone bath. Then, a solution of EtMgBr (1.1 equiv., in diethyl ether) was added dropwise at -78 °C. The resulting solution was stirred for 5 min then allowed to warm to room temperature. Stirring continued for 2 h at the indicated temperature. Upon the completion of the reaction, methyl chloroformate (0.3 mmol, 1.5 equiv.) was added dropwise at the indicated temperature. After stirring for 1 h, the solution concentrated under reduced pressure. Further purification of the crude via column chromatography (0-30% Et_2O in petroleum ether) afforded the final product **5a**.

Table S2. Evaluation of reaction temperature.

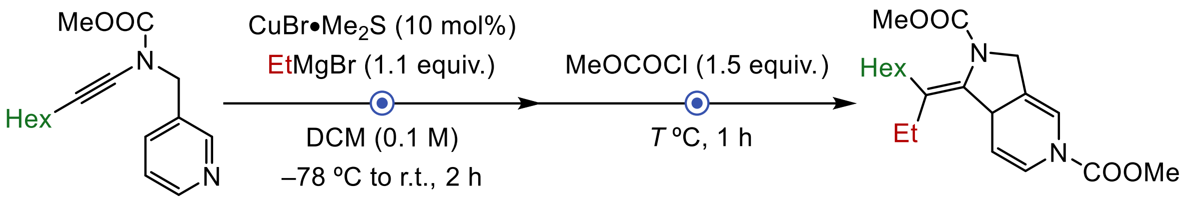

3a
1.0 equiv.

5a

Entry	*T* °C	Yield (%)
1	rt	47
2	0	58
**3**	**–78**	**66**

Reactions were performed under N_2 with 0.2 mmol of **3a**. The reported yields were determined after column chromatography.

Table S3. Evaluation of the effect of additives on the second step.

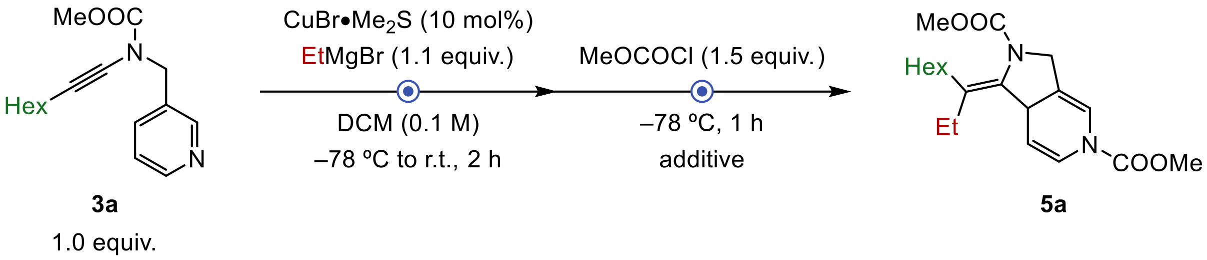

Entry	Additive	Yield (%)
1	THF	47
2	TMEDA	32
3	DMPU	51
4	DMEDA	33
**5**	**none**	**66**

Reactions were performed under N_2 with 0.2 mmol of **3a**. The reported yields were determined after column chromatography.

4. Diazabicycles products

General procedure:

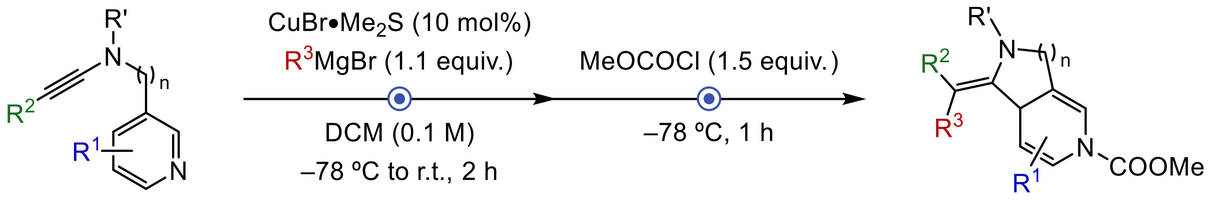

An oven dried Schlenk flask equipped with a stirring bar was allowed to cool to room temperature under vacuum. CuBr•Me_2S complex (10.0 mol%), and ynamide (1.0 equiv.) were added under air. The vessel was then depressurized and pressurized with nitrogen three times, followed by addition of dry DCM (0.1 M). The solution was cooled to -78 °C using dry ice/acetone bath. A solution of R^3MgBr (1.1equiv., in diethyl ether) was added dropwise keeping the indicated temperature. The resulting solution was stirred for 5 min then allowed to warm to room temperature. Stirring continued for 2 h at the indicated temperature (monitored by TLC on silica gel (using 30% ethyl acetate in hexane as eluent). Upon completion of the first step, the reaction temperature decreased to -78 °C and the acylating agents (1.5 equiv.) was added dropwise to reaction mixture. Stirring was continued for 1 h at the indicated temperature (monitored by TLC on silica gel using 30% Et_2O in petroleum ether). Upon the completion of the second step, the resulting solution was concentrated under reduced pressure, and the residue was purified by column chromatography (30% Et_2O in petroleum ether unless otherwise indicated) to afford the final product.

Note: NMR measurements were conducted in C_6D_6 due to the rapid decomposition of the products in $CDCl_3$. Despite the use of concentrated samples, this solvent compromised the quality of the ^{13}C NMR spectra.

Dimethyl (*Z*)-1-(nonan-3-ylidene)-1,7a-dihydro-2*H*-pyrrolo[3,4-c]pyridine-2,5(3*H*)-dicarboxylate (5a)

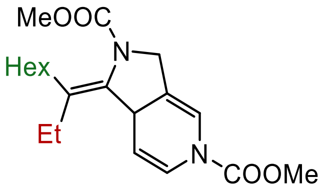

Following general procedure on a 0.3 mmol scale. Purification via column chromatography with 30% Et_2O in petroleum ether. The product was isolated as a yellow oil (71.6 mg, 0.2 mmol, 66%).

For 1 mmol scale, the product was isolated as a yellow oil (202 mg, 0.56 mmol, 56%).

Broadening in NMR spectra was observed due to the rotameric behavior of the N–COOMe groups. The peaks of all rotamers are listed.

^{1}H NMR (400 MHz, C_6D_6) δ 7.06 (bs, 1H), 6.94 (bs, 1H), 6.58 (bs, 1H), 6.38 (bs, 1H), 5.00 (bs, 2H), 4.50 (bs, 2H), 3.57 (s, 2H), 3.53 (d, J = 12.8 Hz, 2H), 3.44 (s, 6H), 3.32 (s, 6H), 2.41 – 2.32 (m, 2H), 2.29 – 2.17 (m, 2H), 2.08 (s, 4H), 1.43 – 1.23 (m, 16H), 1.01 (t, J = 7.2 Hz, 6H), 0.90 (t, J = 6.9 Hz, 6H); **^{13}C NMR** (126 MHz, C_6D_6) δ 155.0, 152.1, 148.9, 133.6, 124.8, 124.4, 118.2, 117.9, 106.9, 106.5, 52.9, 52.5, 50.7, 50.6, 38.3, 37.6, 32.4, 32.3, 32.2, 31.8, 30.2, 30.1, 27.3, 23.1, 14.4, 13.2.

HRMS (ESI) m/z: $[M+H]^+$ Calcd for $C_{20}H_{31}N_2O_4$: 363.2278, Found: 363.2276.

IR ν = 2985 (w), 2862 (w), 1681 (m), 1508 (w), 1458 (w), 1060 (s), 1004 (s), 763 (s).

The dynamics of conformational rotations of N–$COCH_3$ bonds in **5a** was monitored by ^{1}H NMR experiment at different temperatures. At high temperature (318 K), the rotation was fast on the NMR time scale and single and/or broad merged peaks were observed (**Supplementary Fig. 1**). Furthermore, a 2D COSY experiment was performed to aid in structural assignment by confirming key proton–proton correlations (**Supplementary Fig. 2**).

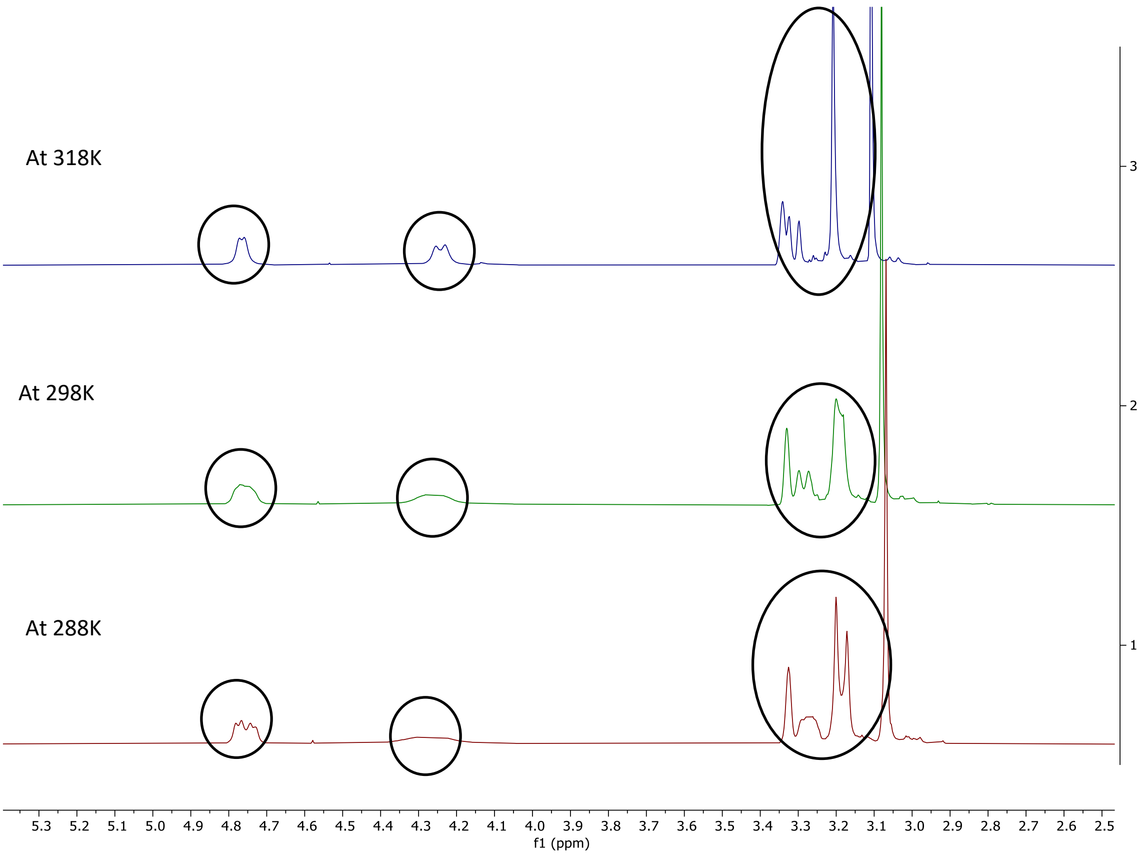

Supplementary Figure 1.^{1}H NMR experiments of **5a** at different temperature in C_6D_6.

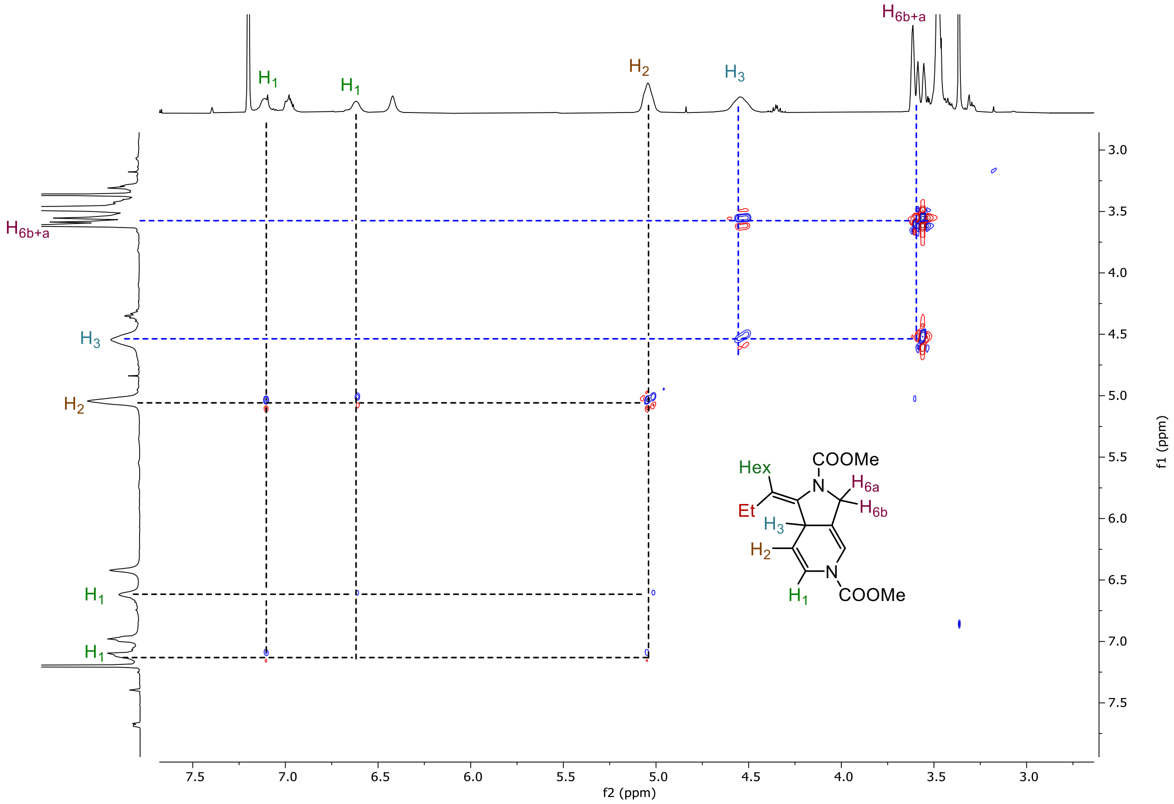

Supplementary Figure 2. 2D COSY experiment for **5a**. Correlation between (H&H2, H3 & H6) was observed.

Dimethyl (*Z*)-1-(octan-2-ylidene)-1,7a-dihydro-2*H*-pyrrolo[3,4-c]pyridine-2,5(3*H*)-dicarboxylate (5b)

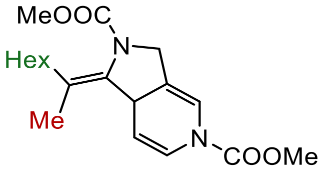

Following general procedure on a 0.3 mmol scale. Purification via column chromatography with 30% Et_2O in petroleum ether. The product was isolated as a yellow oil (63 mg, 0.18 mmol, 61%).

Broadening in NMR spectra was observed due to the rotameric behavior of the N–COOMe groups. The peaks of all rotamers are listed.

^{1}H NMR (500 MHz, C_6D_6) δ 7.05 (bs, 1H), 6.98 (bs, 1H), 6.61 (bs, 1H), 6.42 (bs, 1H), 5.04 (bs, 2H), 4.55 (bs, 2H), 3.57 (s, 4H), 3.48 (s, 6H), 3.37 (s, 6H), 2.26 (s, 4H), 1.63 (s, 6H), 1.36 – 1.28 (m, 16H), 0.95 (t, J = 6.95 Hz, 6H); **^{13}C NMR** (126 MHz, C_6D_6) δ 155.1, 152.1, 133.6 124.7, 124.3, 118.2, 118.0, 117.4, 106.3, 106.0, 52.9, 52.5, 50.6, 38.4, 35.3, 32.3, 30.0, 27.3, 23.1, 17.2, 14.4.

HRMS (ESI) m/z: $[M+H]^+$ Calcd for $C_{19}H_{29}N_2O_4$: 349.2127, Found: 349.2124.

IR ν = 2985 (w), 2900 (w), 1712 (m), 1473 (w), 1334 (w), 1265 (s), 756 (s).

Dimethyl (*Z*)-1-(dodecan-5-ylidene)-1,7a-dihydro-2*H*-pyrrolo[3,4-c]pyridine-2,5(3*H*)-dicarboxylate (5c)

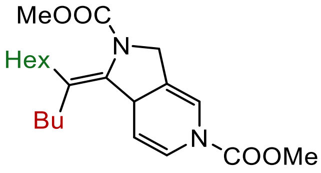

Following general procedure on a 0.3 mmol scale. Purification via column chromatography with 30% Et_2O in petroleum ether. The product was isolated as a yellow oil (67 mg, 0.17 mmol, 57%).

Broadening in NMR spectra was observed due to the rotameric behavior of the N–COOMe groups. The peaks of all rotamers are listed.

^{1}H NMR (500 MHz, C_6D_6) δ 7.18 (bs, 1H), 7.04 (bs, 1H), 6.69 (bs, 1H), 6.49 (bs, 1H), 5.16 (bs, 2H), 4.60 (s, 2H), 3.72 (s, 2H), 3.66 (d, *J* = 12.3 Hz, 2H), 3.55 (bs, 6H), 3.43 (s, 6H), 2.55 – 2.40 (m, 2H), 2.37 – 2.32 (m, 2H), 2.30 – 2.18 (m, 4H), 1.72 – 1.56 (m, 12H), 1.47 – 1.32 (m, 24H), 1.03 – 0.97 (m, 12H); **^{13}C NMR** (126 MHz, C_6D_6) δ 154.5, 151.7, 133.5, 129.4, 124.5, 124.1, 117.8, 117.5, 106.4, 106.1, 52.5, 52.1, 50.1, 38.0, 31.9, 31.7, 30.6, 30.2, 29.7, 26.9, 22.8, 22.7, 13.9, 13.9.

HRMS (ESI) m/z: $[M+H]^+$ Calcd for $C_{22}H_{35}N_2O_4$: 391.2669, Found: 391.2662.

IR ν = 2957 (w), 2954 (w), 2912 (w), 1700 (s), 1444 (m), 1339 (m), 1196 (w), 737 (s).

Dimethyl 1-(tridecan-7-yl)-1,7a-dihydro-2*H*-pyrrolo[3,4-c]pyridine-2,5(3*H*)-dicarboxylate (5d)

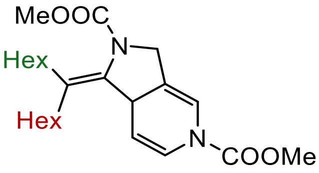

Following general procedure on 0.3 mmol scale. Purification via column chromatography with 30% Et_2O in petroleum ether. The product was isolated as a yellow oil (68 mg, 0.16 mmol, 54%).

Broadening in NMR spectra was observed due to the rotameric behavior of the N–COOMe groups. The peaks of all rotamers are listed.

^{1}H NMR (400 MHz, C_6D_6) δ 7.09 (bs, 1H), 6.94 (bs, 1H), 6.59 (bs, 1H), 6.39 (bs, 1H), 5.10 (bs, 2H), 4.50 (bs, 2H), 3.65 (s, 2H), 3.57 (d, *J* = 12.9 Hz, 2H), 3.45 (s, 6H), 3.33 (s, 6H), 2.44 – 2.32 (m, 2H), 2.32 – 2.23 (m, 2H), 2.15 (t, *J* = 6.9 Hz, 4H), 1.58 – 1.43 (m, 8H), 1.32 – 1.27 (m, 24H), 0.96 – 0.83 (m, 12H); **^{13}C NMR** (101 MHz, C_6D_6) δ 154.9, 152.1, 152.0, 148.9, 144.9, 144.1, 137.3, 134.0, 133.9, 132.6, 129.8, 118.0, 117.4, 117.2, 106.9, 106.5, 53.0, 52.6, 52.3, 50.7, 50.5, 50.3, 38.4, 35.5, 32.3, 32.2, 32.1, 30.1, 29.9, 29.9, 29.8, 29.7, 29.6, 28.9, 27.6, 27.4, 23.1, 23.1, 23.0, 14.4, 14.4, 14.3, 14.3.

HRMS (ESI) m/z: $[M+H]^+$ Calcd for $C_{24}H_{39}N_2O_4$: 419.2909, Found: 419.2900.

IR ν = 2927 (m), 2858 (w), 1714 (s), 1441 (s), 1355 (m), 1264 (w), 1198 (w), 1028 (w), 737 (s).

Dimethyl (*E*)-1-(2-methylnonan-3-ylidene)-1,7a-dihydro-2*H*-pyrrolo[3,4-c]pyridine-2,5(3*H*)-dicarboxylate (5e)

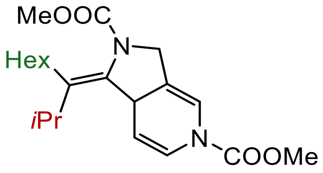

Following general procedure on a 0.3 mmol scale. Purification via column chromatography with 30% Et_2O in petroleum ether. The product was isolated as a yellow oil (44mg, 0.11 mmol, 39%).

Broadening in NMR spectra was observed due to the rotameric behavior of the N–COOMe groups. The peaks of all rotamers are listed.

^{1}H NMR (500 MHz, C_6D_6) δ 7.08 (bs, 1H), 6.96 (bs, 1H), 6.60 (bs, 1H), 6.40 (bs, 1H), 5.02 (bs, 2H), 4.52 (s, 2H), 3.59 (d, J = 1.4 Hz, 2H), 3.44 (s, 6H), 3.34 (s, 6H), 2.65 (bs, 4H), 2.07 –2.01 (m, 2H), 1.67 – 1.54 (m, 2H), 1.52 – 1.40 (m, 2H), 1.36 – 1.26 (m, 16H), 1.08 (d, J = 7.0 Hz, 6H), 0.97 (d, J = 6.8 Hz, 6H), 0.91 (t, J = 5.4 Hz, 6H); **^{13}C NMR** (126 MHz, C_6D_6) δ 152.0, 151.8, 148.7, 148.7, 144.7, 135.0, 133.3, 124.7, 124.3, 118.2, 117.9, 107.2, 106.9, 52.8, 52.3, 50.4, 50.4, 38.1, 31.9, 31.4, 30.4, 28.6, 27.8, 23.5, 22.9, 22.9, 21.8, 20.2, 14.2, 14.1.

HRMS (ESI) m/z: $[M+H]^+$ Calcd for $C_{21}H_{33}N_2O_4$: 377.2434, Found: 377.2422.

IR ν = 2956 (w), 2914 (w), 1726(s), 1439 (s), 1277 (m), 1197 (w), 1098 (w), 1028 (w), 751 (s).

Methyl (*E*)-2-(2-methoxy-2-oxoethyl)-1-(1-phenylheptylidene)-1,2,3,7a-tetrahydro-5*H*-pyrrolo[3,4-c]pyridine-5-carboxylate (5f)

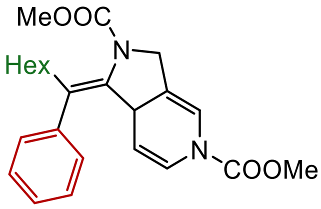

Following general procedure on a 0.3 mmol scale. Purification via column chromatography with 30% Et_2O in petroleum ether. The product was isolated as a yellow oil (63 mg, 0.15 mmol, 51%).

Broadening in NMR spectra was observed due to the rotameric behavior of the N–COOMe groups. The peaks of all rotamers are listed. Fast decomposition of the product was observed during the measurement.

^{1}H NMR (400 MHz, C_6D_6) δ 7.24 – 7.19 (m, 6H), 7.16 – 7.13 (m, 2H), 7.11 – 7.08 (m, 2H), 6.84 (bs, 1H), 6.70 (bs, 1H), 6.30 (bs, 1H), 6.18 (bs, 1H), 4.08 (bs, 2H), 4.00 (bs, 4H), 3.74 (s, 2H), 3.52 (bs, 6H), 3.24 (d, *J* = 12.0 Hz, 6H), 2.68 – 2.44 (m, 4H), 1.78 – 1.64 (m, 2H), 1.31 – 1.11 (m, 16H), 0.83 (t, *J* = 7.0 Hz, 6H); **^{13}C NMR** (101 MHz, C_6D_6) δ 154.7, 151.7, 148.3, 144.7, 144.1, 140.7, 135.2, 134.4, 133.4, 133.1, 129.8, 129.2, 129.2, 123.7, 123.3, 117.6, 117.3, 117.0, 104.9, 104.3, 52.8, 52.7, 52.6, 51.3, 49.2, 37.7, 36.2, 32.1, 32.1, 30.3, 30.2, 30.2, 27.2, 27.1, 23.1, 23.0, 14.3.

HRMS (ESI) m/z: $[M+H]^+$ Calcd for $C_{24}H_{31}N_2O_4$: 411.2283, Found: 411.2272.

IR ν = 2972 (m), 1720 (s), 1444 (s), 1342 (m), 1200 (w), 1055 (s), 1031 (s), 1010 (s), 810 (m), 763 (w), 700 (w).

Dimethyl (*E*)-1-(1-(*m*-tolyl)heptylidene)-1,7a-dihydro-2*H*-pyrrolo[3,4-c]pyridine-2,5(3*H*)-dicarboxylate (5h)

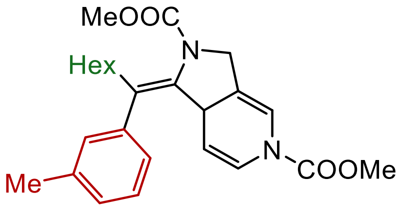

Following general procedure on a 0.3 mmol scale. Purification via column chromatography with 30% Et_2O in petroleum ether. The product was isolated as a yellow oil (45 mg, 0.1 mmol, 35%).

Broadening in NMR spectra was observed due to the rotameric behavior of the N–COOMe groups. The peaks of all rotamers are listed. Fast decomposition of the product was observed during the measurement.

^{1}H NMR (400 MHz, C_6D_6) δ 7.15 – 7.10 (m, 6H), 7.00 – 6.97 (m, 2H), 6.89 (bs, 1H), 6.76 (bs, 1H), 6.35 (bs, 1H), 6.24 (bs, 1H), 4.23 (bs, 1H), 4.14 (bs, 2H), 4.06 (bs, 2H), 3.80 (s, 2H), 3.56 (bs, 6H), 3.27 (d, J = 14.0 Hz, 6H), 2.75 – 2.47 (m, 4H), 2.16 (s, 6H), 1.81 (bs, 2H), 1.41 – 1.21 (m, 16H), 0.88 (t, J = 6.9 Hz, 6H); **^{13}C NMR** (101 MHz, C_6D_6) δ 154.7, 148.4, 144.7, 144.2, 141.3, 140.7, 138.9, 135.5, 134.3, 133.4, 133.1, 130.5, 129.9, 129.5, 117.6, 117.3, 117.1, 116.7, 116.1, 105.1, 104.8, 104.5, 52.8, 52.7, 52.6, 51.4, 50.5, 49.9, 39.5, 37.8, 36.3, 35.1, 32.2, 32.1, 32.1, 30.5, 30.4, 30.3, 30.2, 27.6, 27.3, 27.1, 23.1, 23.1, 23.0, 21.7, 21.4, 21.4, 21.4, 21.1, 14.3, 14.3.

HRMS (ESI) m/z: $[M+H]^+$ Calcd for $C_{25}H_{33}N_2O_4$: 425.2434, Found: 425.2405.

Dimethyl (*E*)-1-(1-(*p*-tolyl)octylidene)-1,7a-dihydro-2*H*-pyrrolo[3,4-c]pyridine-2,5(3*H*)-dicarboxylate (5i)

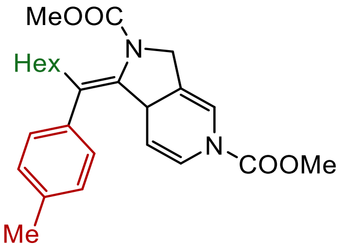

Following general procedure on a 0.3 mmol scale. Purification via column chromatography with 30% Et_2O in petroleum ether. The product was isolated as a yellow oil (56 mg, 0.13 mmol, 44%).

Broadening in NMR spectra was observed due to the rotameric behavior of the N–COOMe groups. The peaks of all rotamers are listed. Fast decomposition of the product was observed during the measurement.

^{1}H NMR (400 MHz, C_6D_6) δ 7.07– 7.01 (m, 4H), 6.89 (bs, 1H), 6.74 (bs, 1H), 6.34 (bs, 1H), 6.23 (bs, 1H), 4.27 (bs, 1H), 4.17 (d, *J* = 7.9 Hz, 1H), 4.09 (d, *J* = 10.4 Hz, 1H), 4.06 (s, 2H), 3.80 (s, 2H), 3.56 (s, 6H), 3.26 (d, *J* = 12.8 Hz, 6H), 2.74 – 2.47 (m, 4H), 2.18 (s, 6H), 1.78 (bs, 2H), 1.38 – 1.13 (m, 16H), 0.87 (t, *J* = 6.9 Hz, 6H); **^{13}C NMR** (101 MHz, C_6D_6) δ 156.3, 151.8, 148.4, 148.4, 141.9, 136.6, 129.6, 129.4, 129.3, 128.8, 125.6, 123.7, 121.7, 121.2, 117.6, 110.9, 110.4, 105.1, 104.6, 53.0, 52.8, 52.6, 49.9, 41.8, 41.7, 39.4, 36.4, 36.3, 32.2, 32.2, 32.0, 30.4, 29.9, 27.7, 27.3, 23.1, 23.0, 23.0, 22.8, 21.2, 21.1, 21.1, 14.3, 14.3.

HRMS (ESI) m/z: $[M+H]^+$ Calcd for $C_{25}H_{33}N_2O_4$: 425.2434, Found: 425.2416.

Dimethyl (*E*)-1-(1-(*p*-methoxyphenyl)heptylidene)-1,7a-dihydro-2*H*-pyrrolo[3,4-c]pyridine-2,5(3*H*)-dicarboxylate (5j)

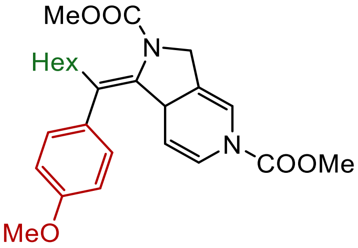

Following general procedure on a 0.3 mmol scale. Purification via column chromatography with 30% Et_2O in petroleum ether. The product was isolated as a yellow oil (50 mg, 0.11 mmol, 38%).

Broadening in NMR spectra was observed due to the rotameric behavior of the N–COOMe groups. The peaks of all rotamers are listed. Fast decomposition of the product was observed during the measurement.

^{1}H NMR (500 MHz, C_6D_6) δ 7.15 – 7.13 (m, 6H), 6.85 (bs, 1H), 6.85 (d, *J* = 9.1 Hz, 4H), 6.75 (bs, 1H), 6.33 (bs, 1H), 6.21 (bs, 1H), 4.25 (bs, 1H), 4.15 (bs, 1H), 4.03 (bs, 3H), 3.77 (s, 2H), 3.52 (s, 6H), 3.33 (s, 6H), 3.26 (s, 6H), 2.63 – 2.56 (m, 2H), 2.53 – 2.42 (m, 2H), 1.74 (bs, 2H), 1.41 – 1.05 (m, 16H), 0.85 (t, *J* = 7.01 Hz, 6H); **^{13}C NMR** (126 MHz, C_6D_6) δ 163.0, 159.8, 159.3, 154.9, 151.8, 148.5, 144.8, 144.3, 135.2, 134.2, 132.5, 130.8, 130.8, 130.4, 123.7, 123.3, 117.6, 117.4, 114.7, 114.3, 114.1, 113.8, 105.2, 104.6, 54.8, 54.8, 52.8, 52.8, 52.6, 49.9, 39.5, 36.3, 32.2, 30.4, 27.3, 23.1, 23.0, 14.3, 14.3.

HRMS (ESI) m/z: $[M+H]^+$ Calcd for $C_{25}H_{33}N_2O_5$: 441.2384, Found: 441.2390.

Dimethyl (*E*)-1-(1-(*p*-(dimethylamino)phenyl)heptylidene)-1,7a-dihydro-2*H*-pyrrolo[3,4-c]pyridine-2,5(3*H*)-dicarboxylate (5k)

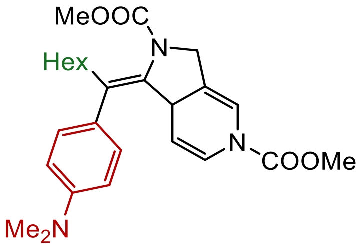

Following general procedure on a 0.3 mmol scale. Purification via column chromatography with 30% Et_2O in petroleum ether. The product was isolated as a yellow oil (40.6 mg, 0.1 mmol, 29%).

Broadening in NMR spectra was observed due to the rotameric behavior of the N–COOMe groups. The peaks of all rotamers are listed. Fast decomposition of the product was observed during the measurement.

^{1}H NMR (500 MHz, C_6D_6) δ 7.23 (bs, 4H), 6.86 (bs,1H), 6.72 (bs, 1H), 6.60 (d, *J* = 7.6 Hz, 4H), 6.31 (bs, 1H), 6.21 (bs, 1H), 4.44 (bs, 1H), 4.34 (bs, 1H), 4.11 (bs, 2H), 4.04 (bs, 2H), 3.88 (s, 2H), 3.53 (d, *J* = 13.1 Hz, 6H), 3.24 (d, *J* = 19.21 Hz, 6H), 2.69 – 2.55 (m, 3H), 2.52 (s, 12H), 1.84 (bs, 2H), 1.54 – 1.45 (m, 3H), 1.43 – 1.29 (m, 18H), 1.27 – 1.22 (m, 10H), 0.96 – 0.87 (m, 4H), 0.84 (t, *J* = 7.1 Hz, 6H); **^{13}C NMR** (126 MHz, C_6D_6) δ 154.9, 151.8, 149.9, 144.6, 133.5, 130.5, 123.6, 123.2, 117.5, 117.2, 112.9, 112.5, 105.5, 105.0, 52.7, 52.6, 49.8, 40.1, 36.4, 32.3, 30.5, 30.2, 27.5, 23.1, 14.4.

HRMS (ESI) m/z: $[M+H]^+$ Calcd for $C_{26}H_{36}N_3O_4$: 454.2700, Found: 454.2684.

Dimethyl (*Z*)-1-(heptan-3-ylidene)-1,7a-dihydro-2*H*-pyrrolo[3,4-c]pyridine-2,5(3*H*)-dicarboxylate (5l)

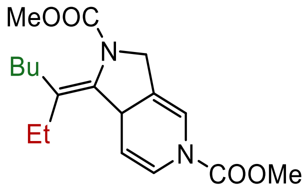

Following general procedure on a 0.3 mmol scale. Purification via column chromatography with 30% Et_2O in petroleum ether. The product was isolated as a yellow oil (62 mg, 0.19 mmol, 63%).

Broadening in NMR spectra was observed due to the rotameric behavior of the N–COOMe groups. The peaks of all rotamers are listed.

^{1}H NMR (400 MHz, C_6D_6) δ 7.05 (bs, 1H), 6.94 (bs, 1H), 6.57 (bs, 1H), 6.38 (bs, 1H), 4.99 (bs, 2H), 4.50 (bs, 2H), 3.56 (s, 2H), 3.52 (d, *J* = 12.9 Hz, 2H), 3.43 (s, 6H), 3.33 (s, 6H), 2.39 – 2.29 (m, 2H), 2.25 – 2.16 (m, 2H), 2.06 (s, 4H), 1.54 – 1.42 (m, 4H) 1.41 – 1.25 (m, 8H), 0.99 (t, *J* = 7.8 Hz, 6H), 0.92 (t, *J* = 7.3 Hz, 6H); **^{13}C NMR** (101 MHz, C_6D_6) δ 155.2, 152.1, 133.6, 130.8, 124.8, 124.4, 117.9, 106.9, 106.5, 52.9, 52.5, 50.5, 38.3, 31.4, 30.5, 30.2, 29.4, 23.9, 23.4, 14.3, 13.2.

HRMS (ESI) m/z: $[M+H]^+$ Calcd for $C_{18}H_{27}N_2O_4$: 335.1965, Found: 335.1958.

IR ν = 2988 (w), 2924 (w), 1680 (s), 1345 (m), 1254 (m), 1198 (w), 734 (s).

Dimethyl (*E*)-1-(undecan-5-ylidene)-1,7a-dihydro-2*H*-pyrrolo[3,4-c]pyridine-2,5(3*H*)-dicarboxylate (5m)

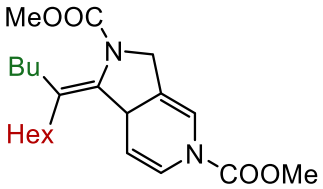

Following general procedure on a 0.3 mmol scale. Purification via column chromatography with 30% Et_2O in petroleum ether. The product was isolated as a yellow oil (64.3 mg, 0.16mmol, 55%).

Broadening in NMR spectra was observed due to the rotameric behavior of the N–COOMe groups. The peaks of all rotamers are listed.

^{1}H NMR (500 MHz, C_6D_6) δ 7.07 (bs, 1H), 6.94 (bs, 1H), 6.58 (bs, 1H), 6.39 (bs, 1H), 5.08 (bs, 2H), 4.49 (bs, 2H), 3.63 (s, 2H), 3.56 (d, *J* = 12.9 Hz, 2H), 3.43 (s, 6H), 3.33 (s, 6H), 2.36 – 2.30 (m, 2H), 2.24 – 2.20 (m, 2H), 2.19 – 2.05 (m, 4H), 1.53 – 1.43 (m, 8H), 1.36 – 1.13 (m, 24H), 0.92 (t, *J* = 7.4 Hz, 6H), 0.89 (t, *J* = 6.6 Hz, 6H); **^{13}C NMR** (126 MHz, C_6D_6) δ 155.0, 152.1, 148.9, 144.9, 144.1, 137.2, 134.0, 129.4, 124.9, 124.5, 118.2, 118.0, 117.4, 117.2, 106.9, 106.5, 52.9, 52.5, 50.5, 38.4, 32.2, 32.1, 31.8, 30.9, 29.8, 29.5, 28.9, 23.4, 23.1, 14.3, 14.3.

HRMS (ESI) m/z: $[M+H]^+$ Calcd for $C_{22}H_{35}N_2O_4$: 391.2591, Found: 391.2586.

Dimethyl (*Z*)-1-(decan-3-ylidene)-1,7a-dihydro-2*H*-pyrrolo[3,4-c]pyridine-2,5(3*H*)-dicarboxylate (5n)

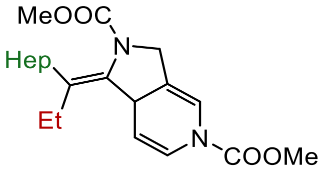

Following general procedure on a 0.3 mmol scale. Purification via column chromatography with 30% Et_2O in petroleum ether. The product was isolated as a yellow oil (66.5 mg, 0.18 mmol, 59%).

Broadening in NMR spectra was observed due to the rotameric behavior of the N–COOMe groups. The peaks of all rotamers are listed.

^{1}H NMR (400 MHz, C_6D_6) δ 7.13 (bs, 1H), 6.98 (bs, 1H), 6.62 (bs, 1H), 6.42 (bs, 1H), 5.05 (bs, 2H), 4.55 (bs, 2H), 3.62 (s,2H), 3.58 (d, *J* = 11.9 Hz, 2H), 3.48 (s, 6H), 3.37 (s, 6H), 2.42 (bs, 2H), 2.42 (bs, 2H), 2.28 (bs, 4H), 1.16 – 1.49 (m, 8H), 1.39 – 1.24 (m, 30H), 1.04 (t, *J* = 7.8 Hz, 8H), 0.95 (t, *J* = 6.8 Hz, 12H); **^{13}C NMR** (101 MHz, C_6D_6) δ 152.1, 133.6, 131.7, 118.2, 118.1, 118.0, 116.7, 106.9, 52.9, 52.6, 50.6, 40.4, 38.3, 32.3, 31.8, 30.5, 30.4, 30.2, 29.8, 27.3, 23.9, 23.1, 14.4, 13.3.

HRMS (ESI) m/z: $[M+H]^+$ Calcd for $C_{21}H_{33}N_2O_4$: 377.2440, Found: 377.2437.

IR ν = 2924 (w), 2854 (w), 1705 (s), 1442 (m), 1350 (m), 1237 (s), 1203 (w), 756 (s).

Dimethyl (*Z*)-1-(1-cyclohexylpropylidene)-1,7a-dihydro-2*H*-pyrrolo[3,4-c]pyridine-2,5(3*H*)-dicarboxylate (5o)

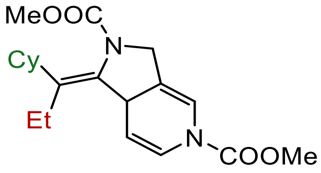

Following general procedure on a 0.3 mmol scale. Purification via column chromatography with 30% Et_2O in petroleum ether.The product was isolated as a yellow oil (51.8 mg, 0.14 mmol, 48%).

Broadening in NMR spectra was observed due to the rotameric behavior of the N–COOMe groups. The peaks of all rotamers are listed. Fast decomposition of the product was observed during the measurement.

^{1}H NMR (400 MHz, C_6D_6) δ 7.05 (bs, 1H), 6.92 (bs, 1H), 6.55 (bs, 1H), 6.36 (bs, 1H), 5.07 (bs, 2H), 4.38 (bs, 2H), 3.57 (s, 4H), 3.45 (s, 6H), 3.31 (s, 6H), 2.63 (bs, 2H), 2.27 – 2.12 (m, 2H), 2.05– 1.95 (m, 4H), 1.74 (s, 6H), 1.64 (d, J = 12.1 Hz, 2H), 1.37 – 1.24 (m, 12H), 1.11 – 0.99 (m, 8H); **^{13}C NMR** (101 MHz, C_6D_6) δ 155.3, 151.6, 148.6, 144.3, 135.6, 133.2, 124.5, 119.7, 117.0, 113.8, 108.9, 105.4, 52.5, 52.1, 50.0, 44.1, 43.0, 38.2, 37.6, 31.0, 30.1, 29.9, 26.9, 26.9, 26.6, 26.4, 26.2, 20.8, 20.1, 15.9.

HRMS (ESI) m/z: $[M+H]^+$ Calcd for $C_{20}H_{29}N_2O_4$: 361.2121, Found: 361.2113.

IR ν = 2925 (w), 2913 (w), 1700 (m), 1441 (m), 1339 (m), 1265 (m), 738 (s).

Dimethyl (*Z*)-1-(3-(pivaloyloxy)propylidene)-1,7a-dihydro-2*H*-pyrrolo[3,4-c]pyridine-2,5(3*H*)-dicarboxylate (5p)

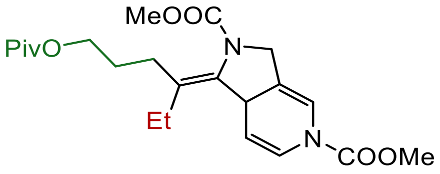

Following general procedure on a 0.3 mmol scale. Purification via column chromatography with 30% Et_2O in petroleum ether. The product was isolated as a yellow oil (63 mg, 0.14 mmol, 50%).

Broadening in NMR spectra was observed due to the rotameric behavior of the N–COOMe groups. The peaks of all rotamers are listed. Fast decomposition of the product was observed during the measurement.

^{1}H NMR (400 MHz, C_6D_6) δ 7.08 (bs, 1H), 6.93 (bs, 1H), 6.58 (bs, 1H), 6.30 (bs, 1H), 4.95 (bs, 2H), 4.45 (s, 2H), 4.06 (t, *J* = 6.6 Hz, 4H), 3.53 (bs,4H), 3.42 (bs, 6H), 3.33 (s, 6H), 2.34 – 2.27 (m, 2H), 2.25 – 2.13 (m, 2H), 2.03 – 1.98 (m, 4H), 1.77 (bs, 2H), 1.68 (bs, 2H), 1.21 (s, 18H) 0.93 (t, *J* = 7.14 Hz, 6H); **^{13}C NMR** (101 MHz, C_6D_6) δ 177.8, 152.1, 148.9, 144.9, 134.4, 124.9, 120.3, 118.0, 117.2, 112.2, 64.5, 53.3, 52.9, 52.6, 50.7, 50.5, 38.9, 38.3, 28.0, 27.4, 27.4, 26.6, 26.4, 24.4, 23.7, 22.8, 13.6, 13.1, 11.7.

HRMS (ESI) m/z: $[M+H]^+$ Calcd for$C_{22}H_{33}N_2O_6$: 421.2333, Found: 421.2317.

IR ν = 2973 (w), 2913 (w), 1718 (s), 1559 (m), 1507 (m), 1267 (m), 1065 (w), 751 (s).

Dimethyl (*Z*)-1-(1-((*tert*-butyldimethylsilyl)oxy)pentan-3-ylidene)-1,7a-dihydro-2*H*-pyrrolo[3,4-c]pyridine-2,5(3*H*)-dicarboxylate (5q)

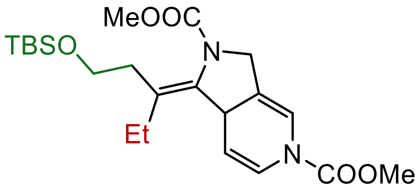

Following general procedure on a 0.3 mmol scale. Purification via column chromatography with 30% Et_2O in petroleum ether. The product was isolated as a yellow oil (50 mg, 0.11 mmol, 37%).

Broadening in NMR spectra was observed due to the rotameric behavior of the N–COOMe groups. The peaks of all rotamers are listed. Traces of grease were observed in ^{1}H and ^{13}C NMR spectra.

^{1}H NMR (500 MHz, C_6D_6) δ 7.04 (bs, 1H), 6.93 (bs, 1H), 6.55 (bs, 1H), 6.37 (bs, 1H), 4.98 (bs, 2H), 4.43 (bs, 2H), 3.86 (s, 4H), 3.56 (s, 2H), 3.15 (d, *J* = 12.1 Hz, 2H), 3.41 (s, 6H), 3.33 (s, 6H), 2.76 – 2.67 (m, 2H), 2.55 – 2.50 (m, 2H), 2.12 (s, 4H), 1.35 – 1.28 (m, 4H), 1.10 – 0.77 (m, 24H), 0.11 (s, 12H); **^{13}C NMR** (126 MHz, C_6D_6) δ 154.7, 152.1, 135.4, 134.9, 133.4, 124.9, 124.4, 118.2, 117.9, 117.2, 106.7, 106.3, 61.7, 61.6, 53.0, 52.6, 52.6, 50.5, 38.3, 35.4, 26.2, 26.2, 18.5, 18.5, 13.5, 13.3, -5.0, -5.1.

HRMS (ESI) m/z: $[M+H]^+$ Calcd for $C_{22}H_{37}N_2O_5Si$: 437.2466, Found: 437.2475.

Methyl (*Z*)-2-(methylsulfonyl)-1-(nonan-3-ylidene)-1,2,3,7a-tetrahydro-5*H*-pyrrolo[3,4-c]pyridine-5-carboxylate (5r)

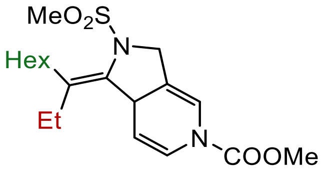

Following general procedure on a 0.3 mmol scale. Purification via column chromatography with 30% Et_2O in petroleum ether. The product was isolated as a yellow oil (49 mg, 0.13 mmol, 43%).

Broadening in NMR spectra was observed due to the rotameric behavior of the N–COOMe and N–SO_2Me groups. The peaks of all rotamers are listed.

^{1}H NMR (400 MHz, C_6D_6) δ 6.92 (bs, 1H), 6.81 (bs, 1H), 6.46 (bs, 1H), 6.27 (bs, 1H), 4.76 (d, *J* = 7.6 Hz, 2H), 4.21 (d, *J* = 12.5 Hz, 2H), 3.48 – 3.44 (m, 2H), 3.41 (dt, *J* = 14.3, 1.9 Hz, 2H), 3.37 (s, 6H), 2.96 – 2.82 (m, 2H), 2.63 – 2.51 (m, 2H), 2.21 (s, 6H), 1.64 – 1.50 (m, 2H), 1.97 (q, *J* = 6.9 Hz, 4H), 1.50 – 1.26 (m, 16H), 0.98 – 0.81 (m, 12H); **^{13}C NMR** (101 MHz, C_6D_6) δ 152.0, 139.9, 139.6, 133.5, 125.9, 124.1, 118.3, 108.4, 107.3, 53.1, 52.8, 38.7, 38.1, 32.3, 31.6, 30.5, 30.2, 29.9, 27.7, 24.2, 23.1, 14.4, 12.9.

HRMS (ESI) m/z: $[M+H]^+$ Calcd for $C_{19}H_{31}N_2O_4S$: 383.1842, Found: 383.1837.

IR ν = 2956 (w), 2860 (w), 1722 (s), 1684 (m), 1553 (s), 1441 (m), 1164 (m), 966 (w), 761 (w).

Dimethyl (*Z*)-7a-methyl-1-(nonan-3-ylidene)-1,7a-dihydro-2*H*-pyrrolo[3,4-c]pyridine-2,5(3*H*)-dicarboxylate (5s)

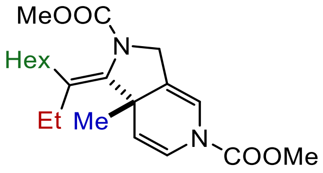

Following general procedure on a 0.3 mmol scale. Purification via column chromatography with 30% Et_2O in petroleum ether. The product was isolated as a yellow oil (35 mg, 0.1 mmol, 31%).

Broadening in NMR spectra was observed due to the rotameric behavior of the N–COOMe groups. The peaks of all rotamers are listed.

^{1}H NMR (500 MHz, C_6D_6) δ 7.08 (bs, 1H), 6.92 (bs, 1H), 6.64 (bs, 1H), 6.37 (bs, 1H), 5.30 (bs, 2H), 4.20 (s, 2H), 4.04 (bs, 2H), 3.52 (s, 6H), 3.35 (s, 6H), 2.37 – 2.12 (m, 8H), 1.66 – 1.56 (m, 2H), 1.54 – 1.45 (m, 2H), 1.37 – 1.28 (m, 12H), 1.19 (s, 6H), 1.14 – 1.03 (m, 6H), 0.96 – 0.86 (m, 6H); **^{13}C NMR** (126 MHz, C_6D_6) δ 155.5, 151.8, 136.7, 123.0, 122.5, 121.5, 116.3, 115.9, 110.8, 110.3, 53.0, 52.6, 48.6, 42.9, 33.2, 32.3, 32.3, 30.5, 30.2, 30.1, 28.2, 27.3, 23.1, 23.1, 14.4, 13.8.

HRMS (ESI) m/z: $[M+H]^+$ Calcd for $C_{21}H_{33}N_2O_4$: 377.2434, Found: 377.2422.

2D COSY and HMBC experiments were performed to aid in structural assignment by confirming key correlations (**Supplementary Fig. 3** and **4**).

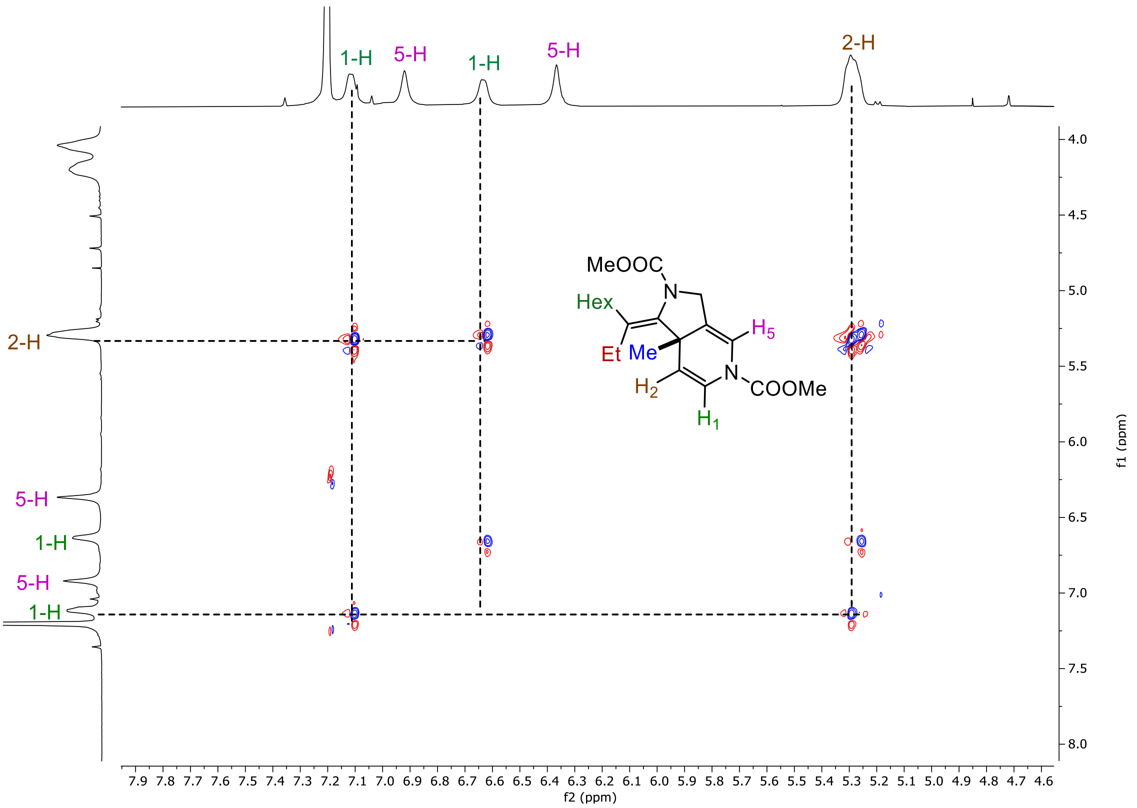

Supplementary Figure 3. 2D COSY experiment of **5s**. Correlation between (H1, H2) was observed.

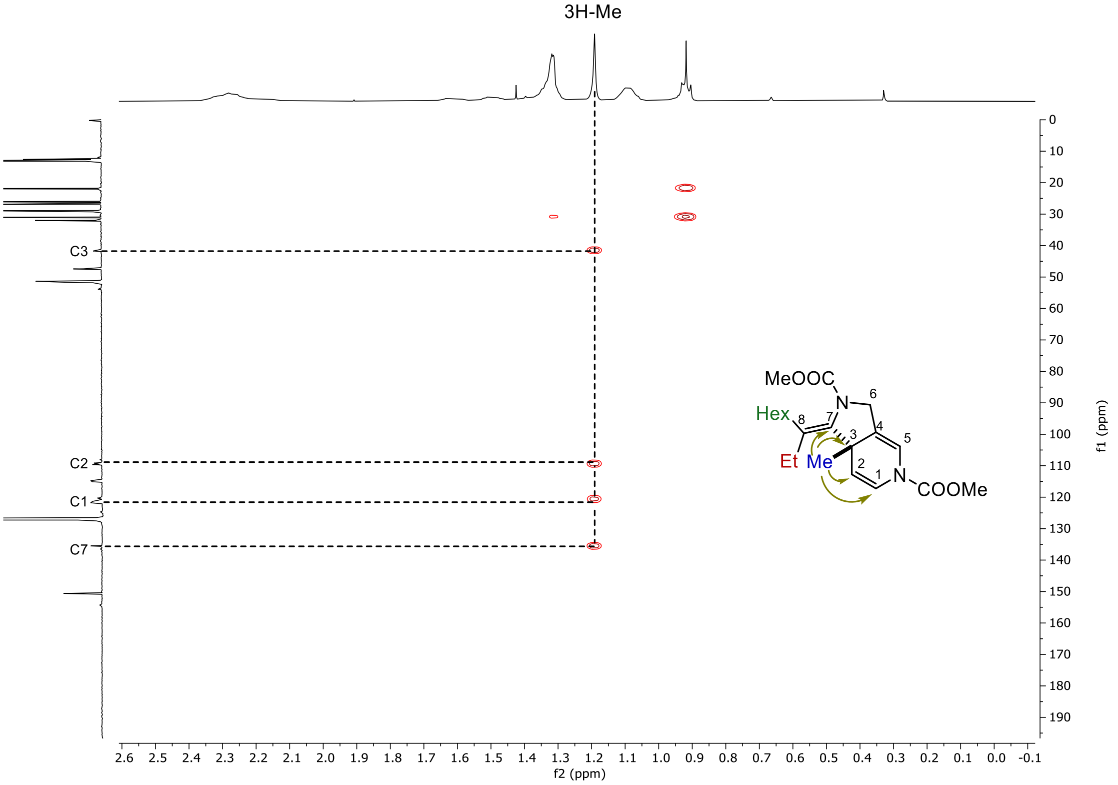

Supplementary Figure 4. 2D HMBC experiment of **5s**. Correlations between the protons of Me group and C1, C2, C3, and C7were observed.

5-Ethyl 2-methyl (*Z*)-1-(nonan-3-ylidene)-1,7a-dihydro-2*H*-pyrrolo[3,4-c]pyridine-2,5(3*H*)-dicarboxylate (5t)

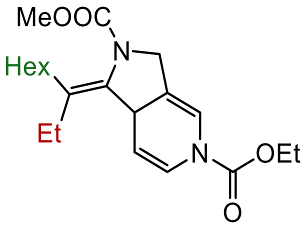

Following general procedure on 0.3 mmol scale. Purification via column chromatography with 30% Et_2O in petroleum ether. The product was isolated as a yellow oil (63 mg, 0.17 mmol, 56%).

Broadening in NMR spectra was observed due to the rotameric behavior of the N–COOMe and N–COOEt groups. The peaks of all rotamers are listed.

^{1}H NMR (400 MHz, C_6D_6) δ 7.11 (bs, 1H), 6.98 (bs,1H), 6.65 (bs, 1H), 6.51 (bs, 1H), 5.02 (bs, 2H), 4.51 (bs, 2H), 3.95 (q, *J* = 6.9 Hz, 4H), 3.60 (bs, 2H), 3.54 (d, *J* = 12.7 Hz, 2H), 3.43 (bs, 6H), 2.37 (bs, 2H), 2.29 – 2.16 (m, 2H), 2.08 (bs, 4H), 1.52 – 1.42 (m, 4H), 1.30 – 1.24 (m, 12H), 1.10 – 0.94 (m, 6H), 0.92 – 0.86 (m, 12H); **^{13}C NMR** (101 MHz, C_6D_6) δ 151.7, 133.7, 130.9, 128.3, 128.1, 127.8, 124.9, 118.7, 118.0, 112.5, 106.8, 62.5, 52.6, 50.6, 38.4, 32.3, 31.8, 30.5, 30.2, 30.1, 27.3, 23.9, 23.1, 23.1, 14.4, 14.4, 13.3.

HRMS (ESI) m/z: $[M+H]^+$ Calcd for $C_{21}H_{33}N_2O_4$: 377.2434, Found: 377.2436.

IR ν = 2929 (w), 2862 (w), 1715 (s), 1439 (m), 1371 (m), 1328 (m), 1119 (m), 765 (s).

5-((9*H*-Fluoren-9-yl)methyl) 2-methyl (*Z*)-1-(nonan-3-ylidene)-1,7a-dihydro-2*H*-pyrrolo[3,4-c]pyridine-2,5(3*H*)-dicarboxylate (5u)

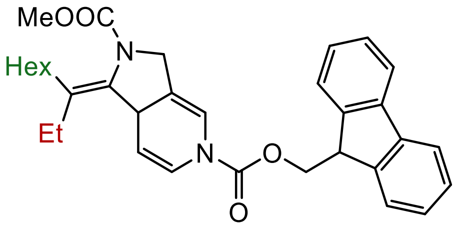

Following general procedure on 0.3 mmol scale. Purification via column chromatography with 30% Et_2O in petroleum ether. The product was isolated as a white solid (60 mg, 0.12 mmol, 40%).

Broadening in NMR spectra was observed due to the rotameric behavior of the N–COOMe and N–Fmoc groups. The peaks of all rotamers are listed.

^{1}H NMR (400 MHz, C_6D_6) 7.56 (d, *J* = 7.09 Hz, 4H), 7.37 (d, *J* = 7.5 Hz, 4H), 7.21 (t, *J* = 7.5Hz, 4H), 7.13 (td, *J* = 7.4, 1.2 Hz, 4H), 7.08 (bs, 1H), 6.92 (bs, 1H), 6.61 (bs, 1H), 6.48 (bs, 1H), 5.05 – 4.90 (m, 2H), 4.50 (bs, 2H), 4.33 (bs, 4H), 3.98 (s, 2H), 3.54 (s, 2H), 3.43 (s, 6H), 2.40 – 2.28 (m,2H), 2.27 – 2.17 (m, 2H), 2.11 – 2.01 (m, 4H), 1.58 – 1.44 (m, 4H), 1.33 – 1.23 (m, 12H), 1.00 (t, *J* = 7.5 Hz, 6H), 0.92 – 0.88 (m, 6H); **^{13}C NMR** (101 MHz, C_6D_6) δ 154.9, 151.6, 145.4, 144.8, 144.1, 141.8, 133.5, 130.9, 125.2, 124.6, 124.1, 120.4, 118.1, 107.1, 68.4, 52.6, 50.5, 47.4, 38.4, 32.3, 31.8, 30.1, 27.3, 23.1, 14.4, 13.3.

HRMS (ESI) m/z: $[M+H]^+$ Calcd for $C_{33}H_{39}N_2O_4$: 527.2904, Found: 527.2903.

IR ν = 2931 (w), 2877 (w), 1712 (m), 1446 (w), 1334 (w), 1273 (s), 1198 (s), 1118 (w) 756 (s).

2-Methyl 5-phenyl (*Z*)-1-(nonan-3-ylidene)-1,7a-dihydro-2*H*-pyrrolo[3,4-c]pyridine-2,5(3*H)*-dicarboxylate (5v)

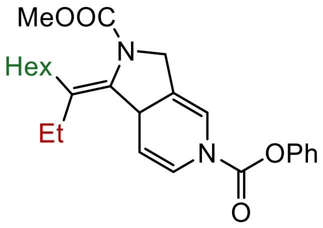

Following general procedure on 0.3 mmol scale. Purification via column chromatography with 30% Et_2O in petroleum ether. The product was isolated as a yellow oil (70 mg, 0.16 mmol, 55%).

Broadening in NMR spectra was observed due to the rotameric behavior of the N–COOMe and N– COOPh groups. The peaks of all rotamers are listed.

^{1}H NMR (400 MHz, C_6D_6) δ 7.14 – 6.96 (m, 12H), 6.95 – 6.81 (m, 6H), 6.81 – 6.75 (m, 4H), 6.75 – 6.68 (m, 3H), 6.59 (bs, 1H), 5.71 (bs, 1H), 5.08 (bs, 1H), 4.47 (d, *J* = 19.6 Hz, 1H), 3.58 (s, 1H), 3.51 (d, *J* = 15.4 Hz, 1H), 3.44 (d, *J* = 10.9 Hz, 3H), 2.34 (s, 1H), 2.24 (s, 1H), 2.16 – 2.01 (m, 2H), 1.54 – 1.40 (m, 2H), 1.40 – 1.22 (m, 8H), 1.11 – 0.95 (m, 4H), 0.95 – 0.82 (m, 6H); **^{13}C NMR** (101 MHz, C_6D_6) δ 158.9, 157.4, 154.0, 152.4, 152.0, 151.7, 151.4, 149.3, 133.2, 131.4, 129.8, 129.7, 129.6, 127.5, 127.1, 126.3, 126.0, 125.9, 125.9, 121.9, 121.4, 121.4, 121.3, 120.5, 120.3, 118.1, 117.9, 115.9, 108.2, 107.6, 64.5, 54.7, 52.7, 52.6, 50.6, 38.4, 32.3, 31.8, 30.1, 27.3, 23.9, 23.1, 23.0, 14.4, 14.3, 13.2.

HRMS (ESI) m/z: $[M+H]^+$ Calcd for $C_{25}H_{33}N_2O_4$: 425.2434, Found: 425.2421.

IR ν = 2930 (w), 2859 (w), 1784 (s), 1736 (w), 1593 (m), 1492 (m), 1353 (m), 1236 (s), 1180 (s), 1119 (s), 854 (w), 743 (m).

Dimethyl (*Z*)-1-(nonan-3-ylidene)-1,3,4,8a-tetrahydro-2,6-naphthyridine-2,6-dicarboxylate (7a)

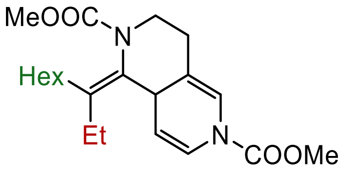

Following general procedure on a 0.3 mmol scale. Purification via column chromatography with 30% Et_2O in petroleum ether. The product was isolated as a yellow oil (72 mg, 0.2 mmol, 64%).

Broadening in NMR spectra was observed due to the rotameric behavior of the N–COOMe groups. The peaks of all rotamers are listed. Fast decomposition of the product was observed during the measurement.

^{1}H NMR (400 MHz, C_6D_6) δ 7.18 (bs, 1H), 7.08 – 6.95 (m, 1H), 6.70 (bs, 0.61H), 6.47 (bs, 0.54H), 4.91 – 4.72 (m, 1H), 4.25 – 4.10 (m, 1H), 3.82 (s, 1H), 3.43 (d, *J* = 8.1 Hz, 3H), 3.40 (d, *J* = 3.5 Hz, 1H), 3.35 (s, 3H), 3.35 (s, 2H), 3.33 – 3.27 (m, 1H), 3.18 (d, *J* = 9.1 Hz, 1H), 2.14 – 2.04 (m, 2H), 2.00 (dd, *J* = 9.3, 5.1 Hz, 2H), 1.95 – 1.90 (m, 2H), 1.86 – 1.77 (m, 2H), 1.33 – 1.17 (m, 16H), 0.89 (t, *J* = 6.8Hz, 12H); **^{13}C NMR** (101 MHz, C_6D_6) δ 56.5, 151.9, 139.2, 131.3, 124.3, 119.4, 116.4, 116.0, 108.4, 107.8, 52.9, 52.8, 52.3, 47.5, 46.9, 38.8, 32.2, 32.1, 32.1, 31.0, 30.5, 30.2, 30.1, 30.0, 27.7, 27.6, 27.3, 23.3, 23.1, 23.0, 14.3, 13.2, 13.0.

HRMS (ESI) m/z: $[M+H]^+$ Calcd for $C_{21}H_{33}N_2O_4$: 377.2434, Found: 377.2424.

Dimethyl (*Z*)-1-(undecan-5-ylidene)-1,3,4,8a-tetrahydro-2,6-naphthyridine-2,6-dicarboxylate (7b)

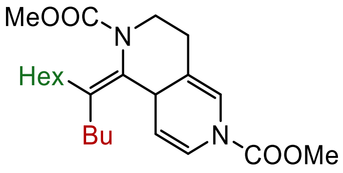

Following general procedure on a 0.3 mmol scale. Purification via column chromatography with 30% Et_2O in petroleum ether. The product was isolated as a yellow oil (70 mg, 0.17 mmol, 58%).

Broadening in NMR spectra was observed due to the rotameric behavior of the N–COOMe groups. The peaks of all rotamers are listed. Fast decomposition of the product was observed during the measurement.

^{1}H NMR (400 MHz, C_6D_6) δ 7.20 (bs, 1H), 7.08 – 6.95 (m, 1H), 6.70 – 6.62 (m, 1H), 6.55 – 6.47 (m, 1H), 5.03 (bs, 1H), 4.91 – 4.72 (m, 2H), 4.25 – 4.10 (m, 2H), 3.97 (s, 1H) 3.82 (s, 2H), 3.43 (d, *J* = 8.1 Hz, 6H), 3.40 (bs, 2H), 3.35 (s, 6H), 3.31 (d, *J* = 9.3 Hz, 2H), 3.33 – 3.27 (m, 2H), 3.18 (d, *J* = 9.1 Hz, 4H), 2.14 – 1.90 (m, 6H), 1.86 – 1.77 (m, 4H), 1.33 – 1.17 (m, 36H), 0.89 (t, *J* = 6.8Hz, 18H); **^{13}C NMR** (101 MHz, C_6D_6) δ156.5, 155.2, 151.9, 138.6, 138.0, 131.7, 124.5, 124.2, 119.8, 119.4, 116.8, 116.1, 108.3, 107.8, 52.9, 52.8, 52.3, 47.5, 47.0, 38.9, 32.2, 32.1, 30.8, 30.7, 30.1, 30.0, 30.0, 27.7, 27.7, 23.3, 23.2, 23.1, 14.4, 14.3.

HRMS (ESI) m/z: $[M+H]^+$ Calcd for $C_{23}H_{37}N_2O_4$: 405.2747, Found: 405.2735.

Dimethyl 1-(tridecan-7-ylidene)-1,3,4,8a-tetrahydro-2,6-naphthyridine-2,6-dicarboxylate (7c)

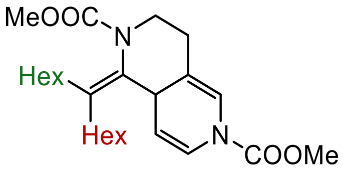

Following general procedure on a 0.3 mmol scale. Purification via column chromatography with 30% Et_2O in petroleum ether. The product was isolated as a yellow oil (70 mg, 0.18 mmol, 52%).

Broadening in NMR spectra was observed due to the rotameric behavior of the N–COOMe groups. The peaks of all rotamers are listed. Fast decomposition of the product was observed during the measurement.

^{1}H NMR (500 MHz, C_6D_6) δ 7.23 – 7.14 (m, 1H) 7.05 – 6.90 (bs, 1H), 7.00 – 6.61 (bs, 0.75H), 6.56 – 6.41 (m, 0.78H), 4.94 – 4.83 (m, 1H), 4.24 – 4.11 (m, 1H), 3.93 (s, 4H), 3.54 – 3.46 (m, 1H), 3.44 (bs, 4H), 3.34 (s, 1H), 2.20 – 2.07 (m, 3H), 2.06 – 1.98 (m, 3H), 1.88 – 1.83 (m, 3H), 1.43 – 1.10 (m, 36H), 0.92 – 0.81 (m, 10H); **^{13}C NMR** (126 MHz, C_6D_6) δ 156.6, 151.9, 138.0, 131.7, 124.5, 124.2, 119.8, 119.4, 108.3, 107.8, 62.7, 52.9, 52.8, 52.3, 47.1, 47.0, 38.9, 33.2, 32.4, 32.2, 32.2, 32.1, 32.0, 30.8, 30.5, 30.2, 30.1, 30.0, 29.9, 29.8, 28.5, 27.7, 27.7, 23.1, 23.1, 23.0, 14.3, 14.3.

HRMS (ESI) m/z: $[M+H]^+$ Calcd for $C_{25}H_{41}N_2O_4$: 433.2737, Found: 433.2731.

Dimethyl (*E*)-1-(1-phenylheptylidene)-1,3,4,8a-tetrahydro-2,6-naphthyridine-2,6-dicarboxylate (7d)

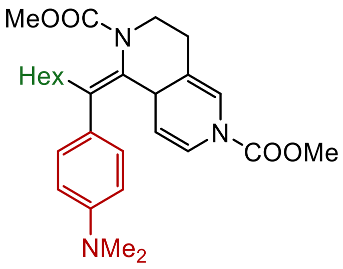

Following general procedure on a 0.3 mmol scale. Purification via column chromatography with 30% Et_2O in petroleum ether. The product was isolated as a yellow oil (41 mg, 0.1 mmol, 29%).

Broadening in NMR spectra was observed due to the rotameric behavior of the N–COOMe groups. The peaks of all rotamers are listed. Fast decomposition of the product was observed during the measurement.

^{1}H NMR (500 MHz, C_6D_6) δ 7.13 (d, *J* = 8.9 Hz, 5H), 6.98 (bs, 1H), 6.92 (bs, 1H), 6.60 (d, *J* = 8.9 Hz, 5H), 6.58 (bs, 1H), 6.40 (bs, 1H), 5.06 (d, *J* = 7.0 Hz, 1H), 4.99 (d, *J* = 7.6 Hz, 1H), 4.20 (m, 2H), 4.03 (s, 1H), 3.95 (s, 2H), 3.64 – 3.59 (m, 6H), 3.52 (s, 2H), 3.39 (s, 3H), 3.34 – 3.29 (m, 6H), 3.26 (d, *J* = 8.25 Hz, 2H), 3.23 (s, 1H), 2.51 (s, 12H), 2.48 – 2.41 (m, 4H), 1.96 (t, *J* = 6.95 Hz,2H), 1.85 (dd, *J* = 12.6, 7.6 Hz, 2H), 1.34 – 0.96 (m, 24H), 0.86 – 0.83 (m, 12H); **^{13}C NMR** (126 MHz, C_6D_6) δ 162.7, 156.5, 155.4, 149.9, 143.2, 141.1, 132.9, 129.6, 129.4, 129.4, 124.1, 121.8, 119.7, 119.3, 117.5, 117.1, 116.8, 113.3, 113.1, 112.8, 112.7, 54.6, 52.8, 52.8, 52.4, 47.5, 42.9, 40.5, 40.3, 40.2, 40.1, 40.0, 34.3, 32.2, 32.1, 31.6, 30.5, 30.2, 29.9, 28.8, 28.6, 27.6, 27.4, 27.3, 23.0, 22.8, 19.4, 14.3.

HRMS (ESI) m/z: $[M+H]^+$ Calcd for $C_{27}H_{38}N_3O_4$: 468.2857, Found: 468.2861.

.

Dimethyl (*Z*)-1-(1-((*tert*-butyldimethylsilyl)oxy)pentan-3-ylidene)-1,3,4,8a-tetrahydro-2,6-naphthyridine-2,6-dicarboxylate (7e)

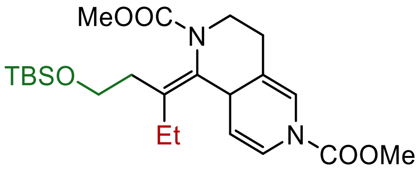

Following general procedure on a 0.3 mmol scale. Purification via column chromatography with 30% Et_2O in petroleum ether. The product was isolated as a yellow oil (39 mg, 0.1 mmol, 34%).

Broadening in NMR spectra was observed due to the rotameric behavior of the N–COOMe groups. The peaks of all rotamers are listed. Fast decomposition of the product was observed during the measurement.

^{1}H NMR (400 MHz, C_6D_6) δ 7.21 – 7.16 (m, 1H), 7.03 – 7.96 (m, 1H), 6.70– 6.60 (m, 0.8H), 6.52 – 6.40 (m, 0.7H), 5.02 – 4.88 (m, 0.5H), 4.83 – 4.71 (m, 1H), 4.23 – 4.14 (m, 1H), 3.81 (s, 1H), 3.72 – 3.57 (m, 3H), 3.54 – 3.37 (m, 3H), 3.34 (s, 2H), 3.23 – 3.12 (m, 3H), 3.29 (d, *J* = 5.1 Hz, 1H), 3.17 (s, 1H), 2.60 – 2.50 (m, 0.8H), 2.51 – 2.33 (m, 2H), 2.28 – 2.18 (m, 2H), 2.05 – 1.91 (m, 4H), 1.82 (d, *J* = 7.8 Hz, 4H), 0.98 (s, 12H), 0.91 (t, *J* = 7.81 Hz, 6H), 0.11 (s, 3H), 0.08 (s, 6H); **^{13}C NMR** (126 MHz, C_6D_6) δ 154.8, 152.2, 135.4, 135.0, 133.5, 125.0, 123.9, 118.0, 117.5, 116.5, 106.8, 105.8, 62.8, 61.7, 61.6, 53.0, 52.7, 50.9, 50.6, 39.1, 38.4, 35.5, 30.6, 30.3, 26.3, 26.2, 26.2, 25.3, 24.8, 18.6, 18.6, 14.5, 13.6, 13.4, -4.9, -5.0.

HRMS (ESI) m/z: $[M+H]^+$ Calcd for $C_{23}H_{39}N_2O_5Si$: 451.2623, Found: 451.2617.

5. Application

5.1 Hydrogenation

General procedure:

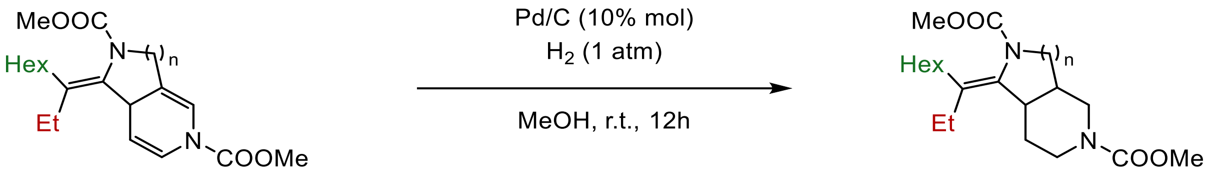

To a solution of 3,4-fused *N*-heterocycle (0.2 mmol, 1.0 equiv.) in MeOH (2.0 mL) was added 10% Pd/C (21.2 mg, 10.0 mol%). The reaction mixture was vigorously stirred for 12h under H_2 atmosphere (1 atm). Upon the completion of the reaction, the solution was filtrated through celite. Filter cake was washed with EtOAc, and the solvent were removed on a rotary evaporator. The crude was purified by column chromatography (silica gel, ethyl acetate in hexane) to afford the final product as a yellow oil.

The following products were obtained:

Dimethyl (*Z*)-1-(nonan-3-ylidene)hexahydro-2*H*-pyrrolo[3,4-c]pyridine-2,5(3*H*)-dicarboxylate (8a)

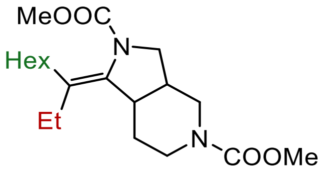

Compound **8a** was prepared according to the general procedure starting from compound **5a** (72.4 mg, 0.2 mmol). The product was isolated as a yellow oil (71.7 mg, 0.19 mmol, 98%).

Broadening in NMR spectra was observed due to the rotameric behavior of the N–COOMe groups which significantly affected ^{1}H and ^{13}C NMR spectra.

^{1}H NMR (500 MHz, $CDCl_3$) δ 4.18 – 3.90 (m, 2H), 3.69 (s, 3H), 3.68 (s, 3H), 3.65 (q, *J* = 1.9 Hz, 1H), 3.49 (dd, *J* = 11.1, 9.2 Hz, 1H), 3.44 – 3.34 (m, 1H), 3.06 (d, *J* = 13.0 Hz, 1H), 2.69 (dt, *J* = 11.9, 6.0 Hz, 1H), 2.11 – 2.24 (m, 1H), 2.07 – 1.96 (m, 2H), 1.91 (ddd, *J* = 14.4, 9.7, 4.8 Hz, 1H), 1.68 – 1.59 (m, 1H), 1.33 – 1.14 (m, 10H), 1.03 – 0.92 (m, 3H), 0.89 – 0.80 (m, 6H); **^{13}C NMR** (126 MHz, $CDCl_3$) δ 155.9, 153.1, 135.3, 127.3, 52.8, 52.2, 50.9, 50.4, 44.9, 42.6, 38.7, 34.1, 32.0, 29.6, 28.3, 27.0, 26.2, 22.8, 14.2, 12.9.

HRMS (ESI) m/z: $[M+H]^+$ Calcd for $C_{20}H_{35}N_2O_4$: 367.2591, Found: 367.2589.

IR ν = 2956 (w), 2920 (w), 2850 (w), 1689 (s), 1450 (m), 1348 (w), 1219 (m), 1116 (w), 763 (s).

Dimethyl (*Z*)-1-(nonan-3-ylidene)octahydro-2,6-naphthyridine-2,6-dicarboxylate (9a)

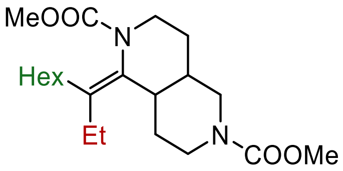

Compound **9a** was prepared according to the general procedure starting from compound **7a** (75.4 mg, 0.2 mmol). The product was isolated as a yellow oil (73.2 mg, 0.19 mmol, 96%).

Broadening in NMR spectra was observed due to the rotameric behavior of the N–COOMe groups which significantly affected ^{1}H and ^{13}C NMR spectra.

^{1}H NMR (500 MHz, $CDCl_3$) δ 4.42 – 4.17 (m, 1H), 4.15 – 3.83 (m, 1H), 3.67 (s, 3H), 3.60 (s, 2H), 3.48 (s, 1H), 3.37 – 3.12 (m, 1H), 3.05 – 2.80 (m, 1H), 2.80 – 2.57 (m, 2H), 2.32 – 2.21 (m, 1H), 2.13 – 2.02 (m, 1H), 2.01 – 1.93 (m, 1H), 1.91 – 1.78 (m, 2H), 1.35 – 1.18 (m, 21H), 1.02 –0.99 (m, 2H), 0.87 (td, J = 7.0, 2.9 Hz, 6H); **^{13}C NMR** (126 MHz, $CDCl_3$) δ 156.4, 156.2, 136.3, 132.8, 54.1, 52.7, 52.5, 51.0, 46.4, 44.1, 40.7, 37.5, 36.9, 32.0, 29.8, 29.5, 23.4, 22.83, 22.7, 14.2, 14.0.

HRMS (ESI) m/z: $[M+H]^+$ Calcd for $C_{21}H_{37}N_2O_4$: 381.2747, Found: 381.2740.

IR ν = 2960 (w), 2922 (w), 2858 (w), 1695 (s), 1450 (m), 1351 (w), 1214 (m), 1120 (w), 766 (s).

6. References

1. Orchin, M. The Grignard reagent: Preparation, Structure, and Some Reactions. *J. Chem. Educ*. **1989**, *66*, 586.
2. Tammiku, J.; Burk P.; Tuulmets, A 1,10-Phenanthroline and Its Complexes with Magnesium Compounds. Disproportionation Equilibria. *J. Phys. Chem. A.* **2001**, *105*, 8554–8561.
3. Tundo, P.; Bressanello, S.; Loris, A.; Sathicq, G. Direct synthesis of *N*-Methylurethanes from Primary Amines with Dimethyl Carbonate. *Pure Appl. Chem*. **2005**, *77*, 1719–1725.
4. Andna, L.; Miesch, L. Metal-free Synthesis of Activated Ynesulfonamides and Tertiary enesulfonamides. *Org. Biomol. Chem.*, **2019**,*17*, 5688–5692.
5. Zhang, Y.-Q.; Chen, Y.-B.; Liu, J.-R.; Wu, S.-Q.; Fan, X.-Y.; Zhang, X.-Y.; Hong, X.; Ye, L.-W. Asymmetric Dearomatization Catalysed by Chiral Brønsted Acids via Activation of Ynamides. *Nat. Chem*. **2021**, *13*, 1093–1100.
6. Agbaria, M.; Egbaria, N.; Nairoukh, Z. Dearomative Spirocyclization of Ynamides. *Chem. Sci*. **2024**, *15*, 19136–19141.

7. NMR Spectra

^{1}H NMR (400 MHz, $CDCl_3$) for **3a**

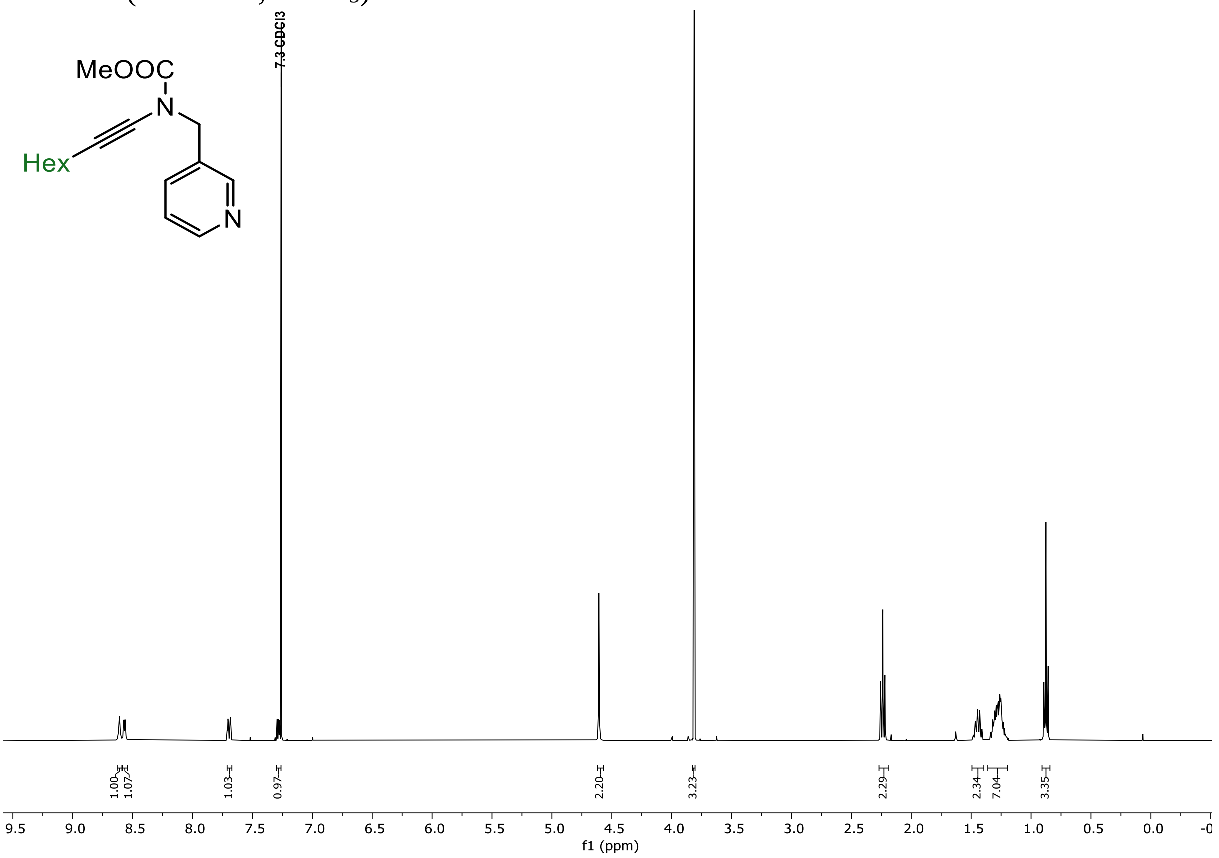

^{13}C NMR (126 MHz, $CDCl_3$) for **3a**

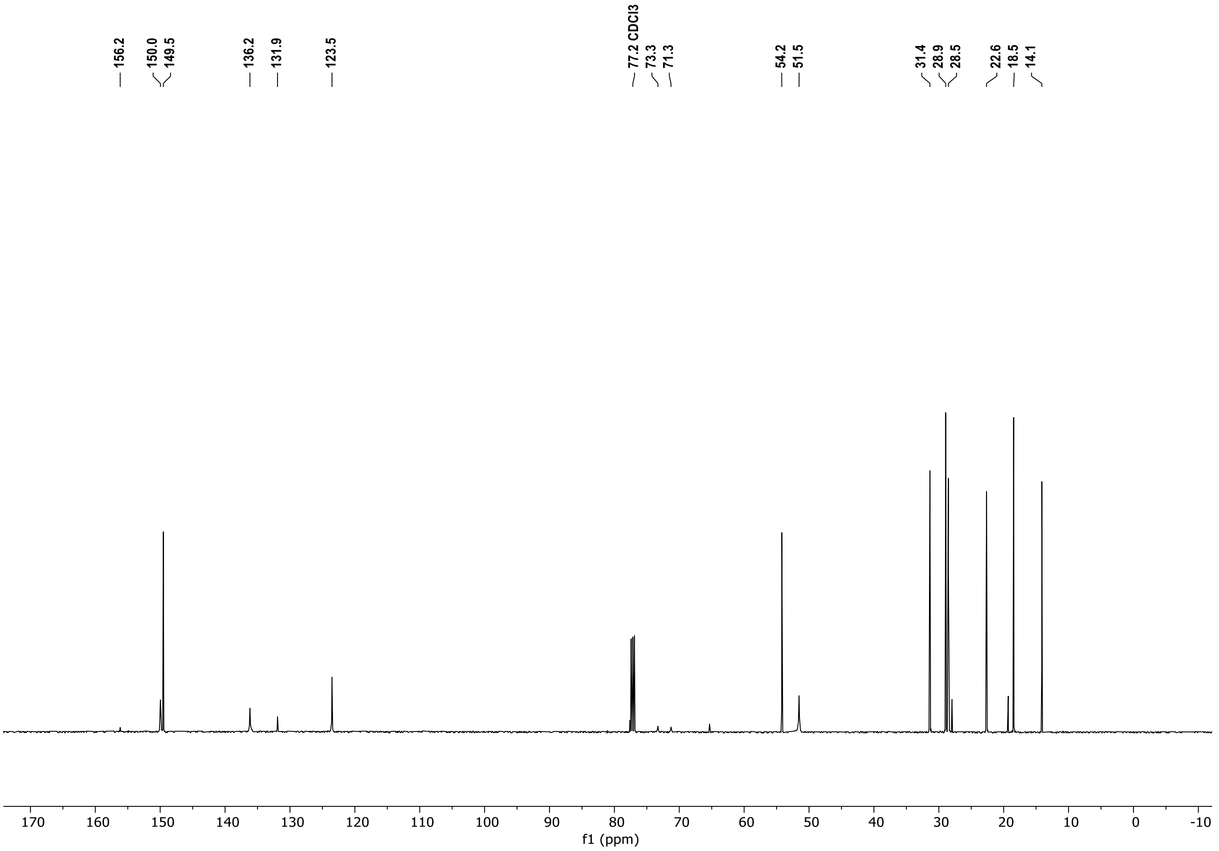

^{1}H NMR (400 MHz, $CDCl_3$) for **3l**

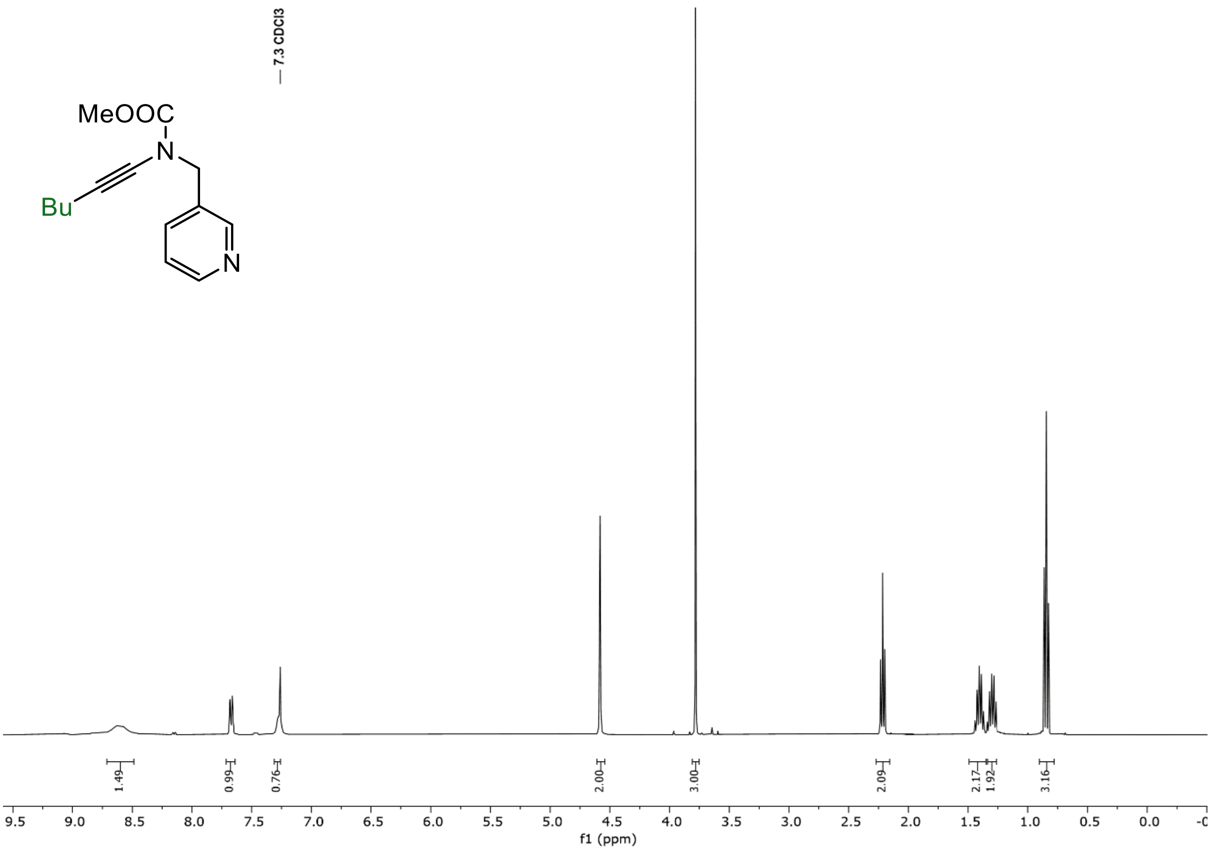

^{13}C NMR (101 MHz, $CDCl_3$) for **3l**

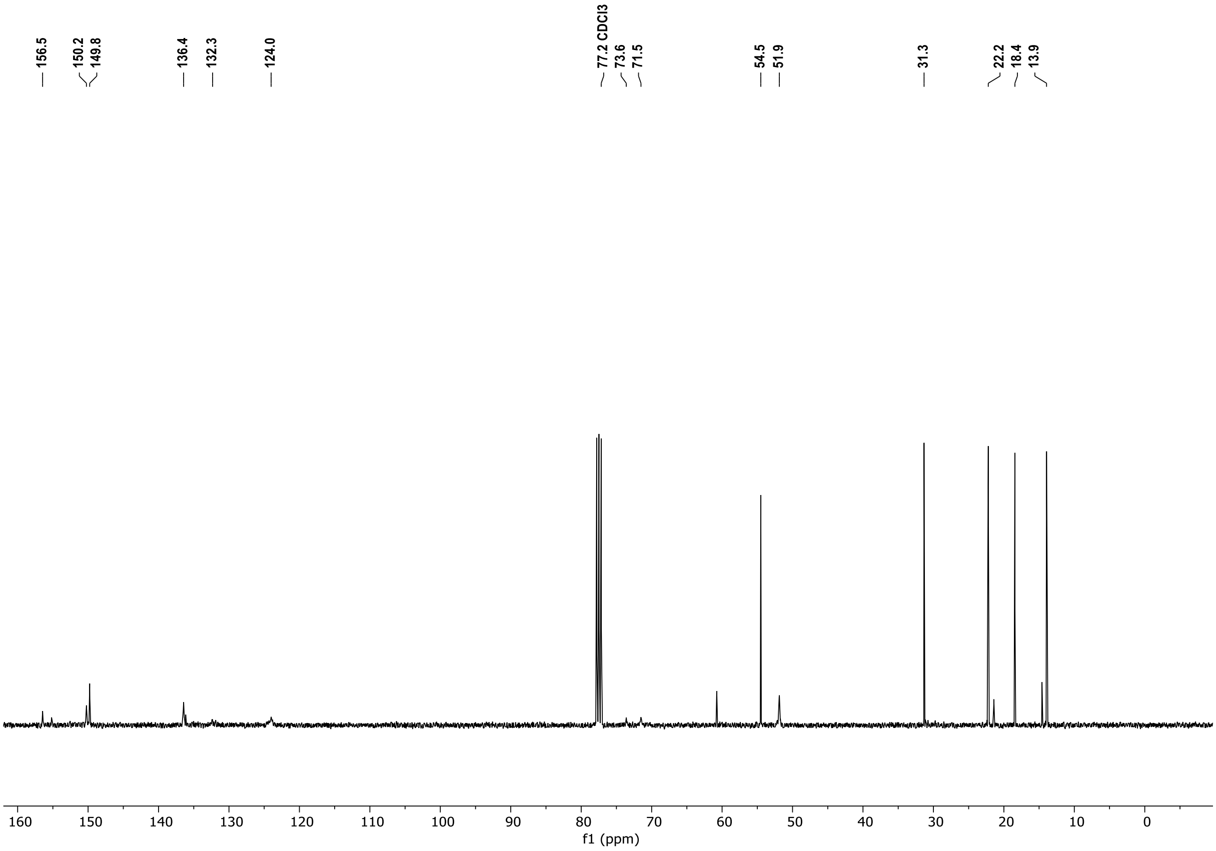

^{1}H NMR (400 MHz, $CDCl_3$) for **3n**

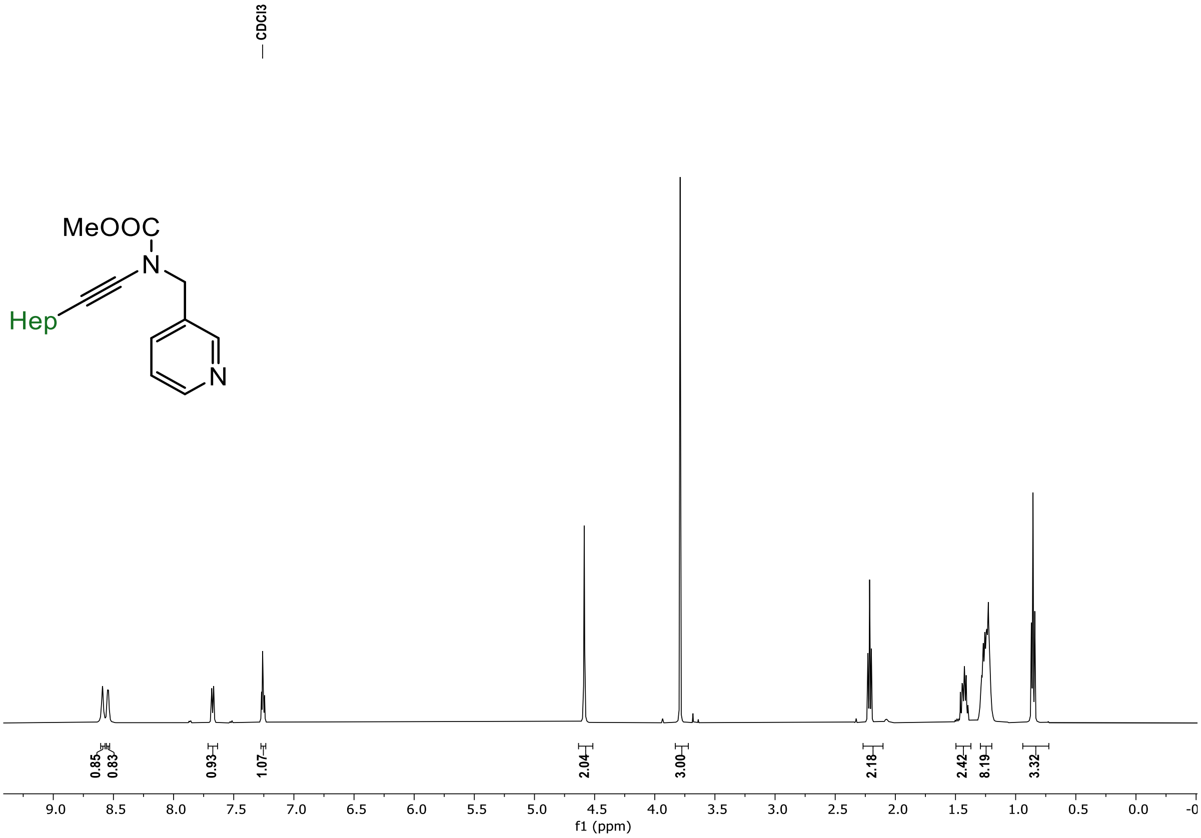

^{13}C NMR (126 MHz, $CDCl_3$) for **3n**

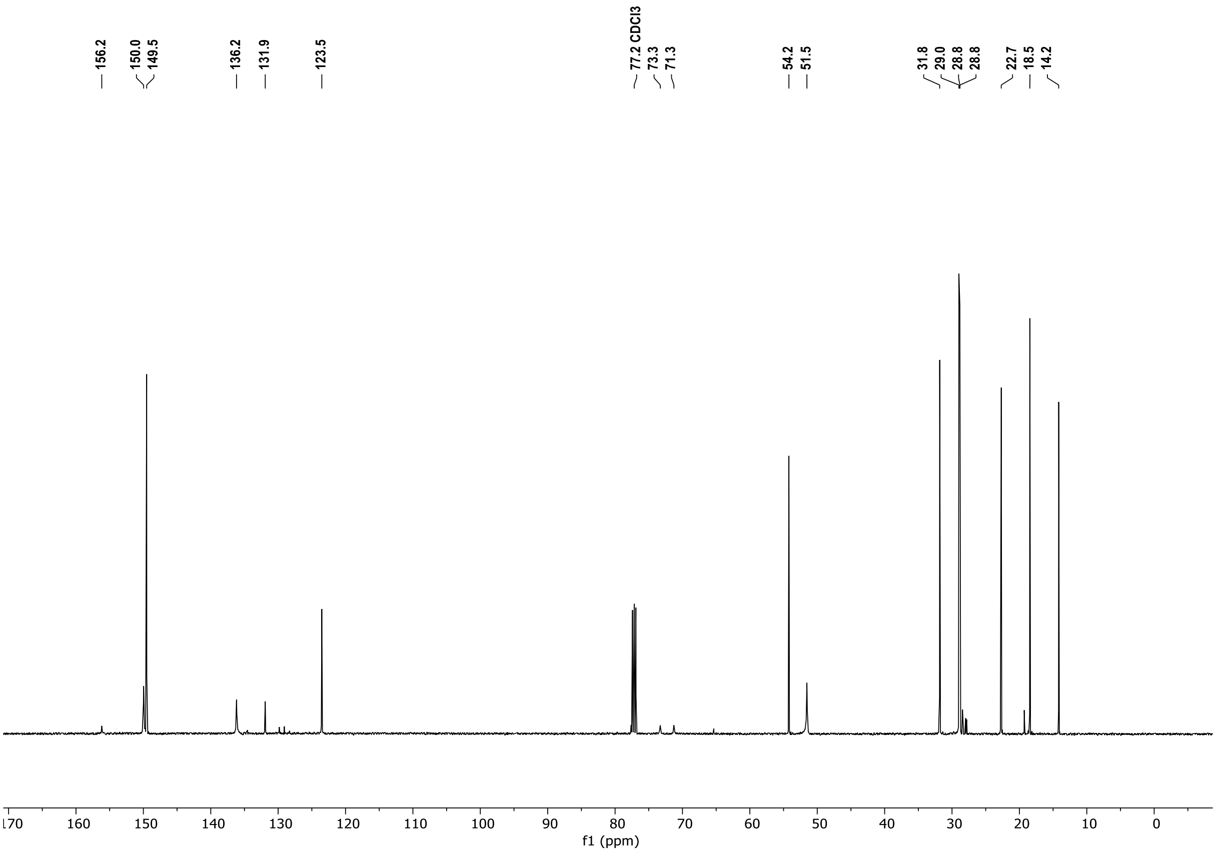

^{1}H NMR (400 MHz, $CDCl_3$) for **3o**

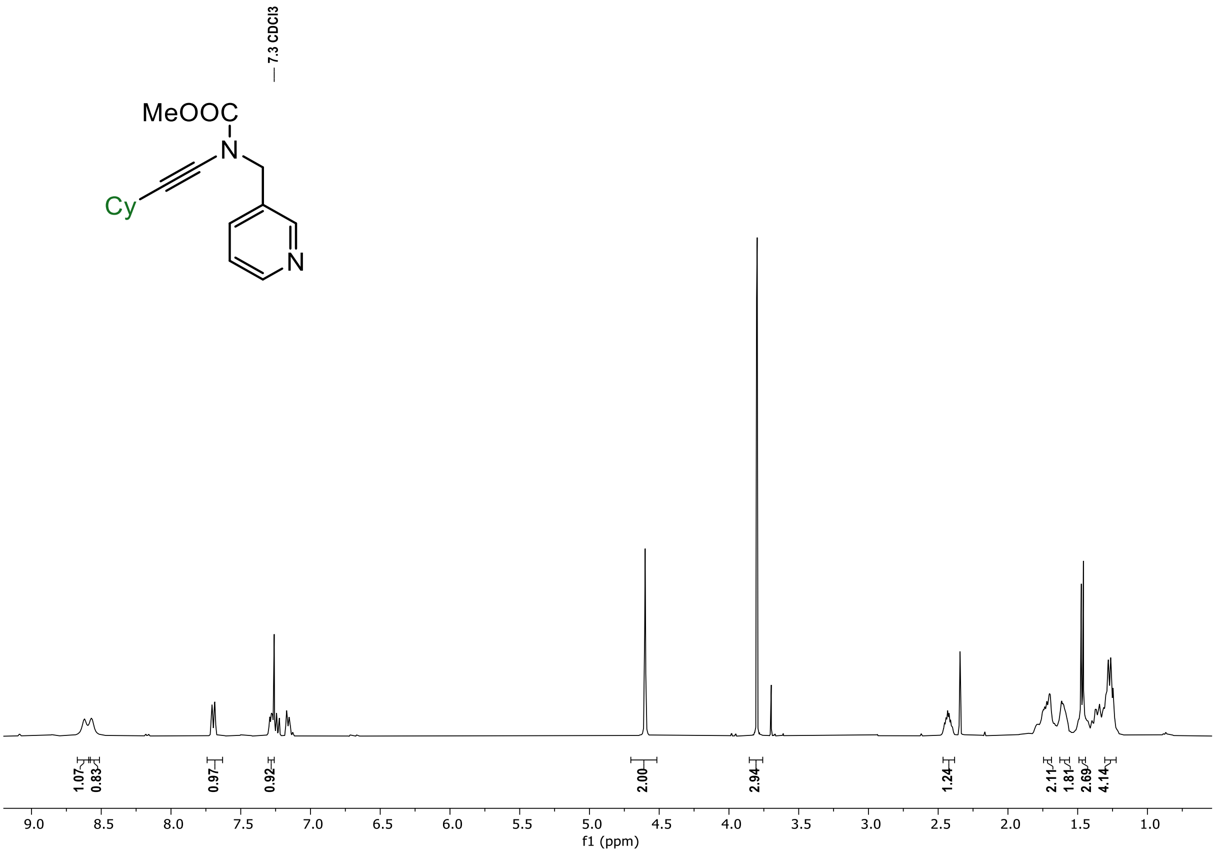

^{13}C NMR (101 MHz, $CDCl_3$) for **3o**

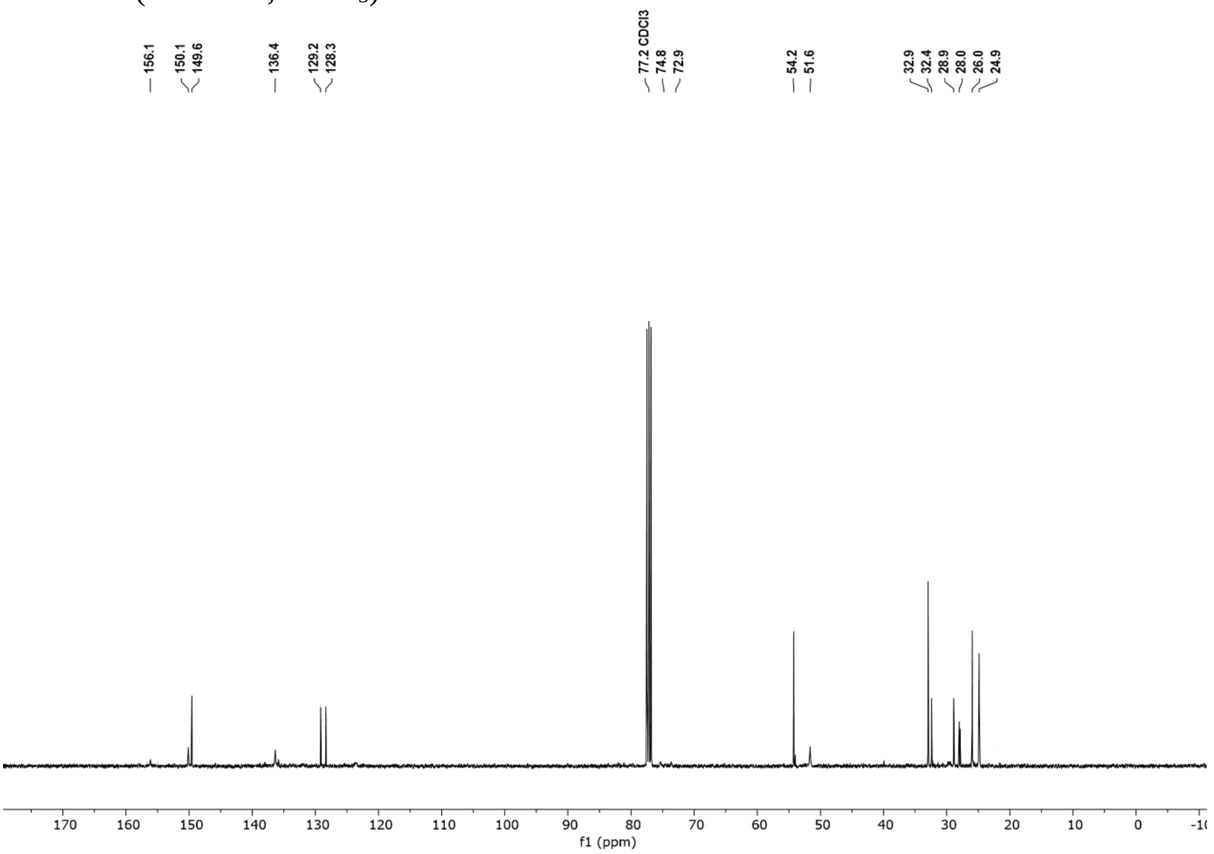

^{1}H NMR (400 MHz, $CDCl_3$) for **3p**

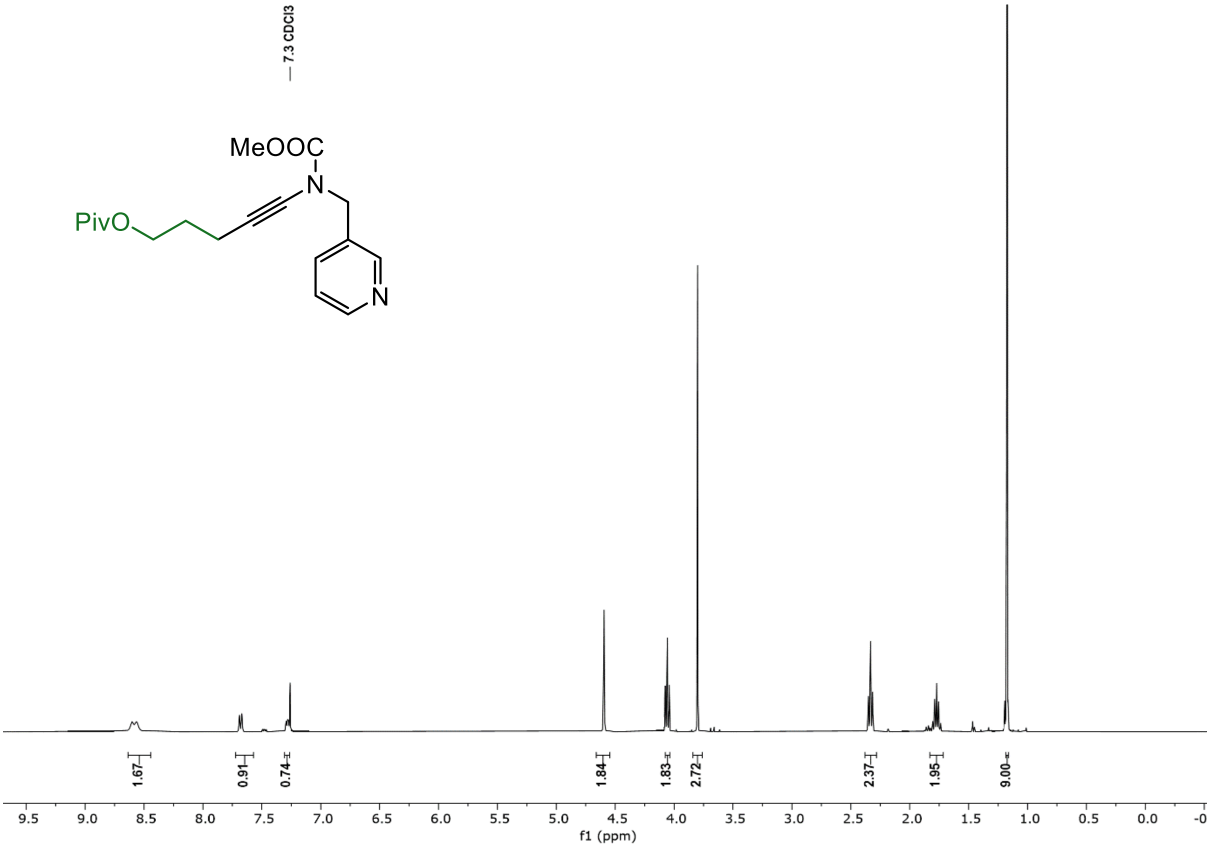

^{13}C NMR (101 MHz, $CDCl_3$) for **3p**

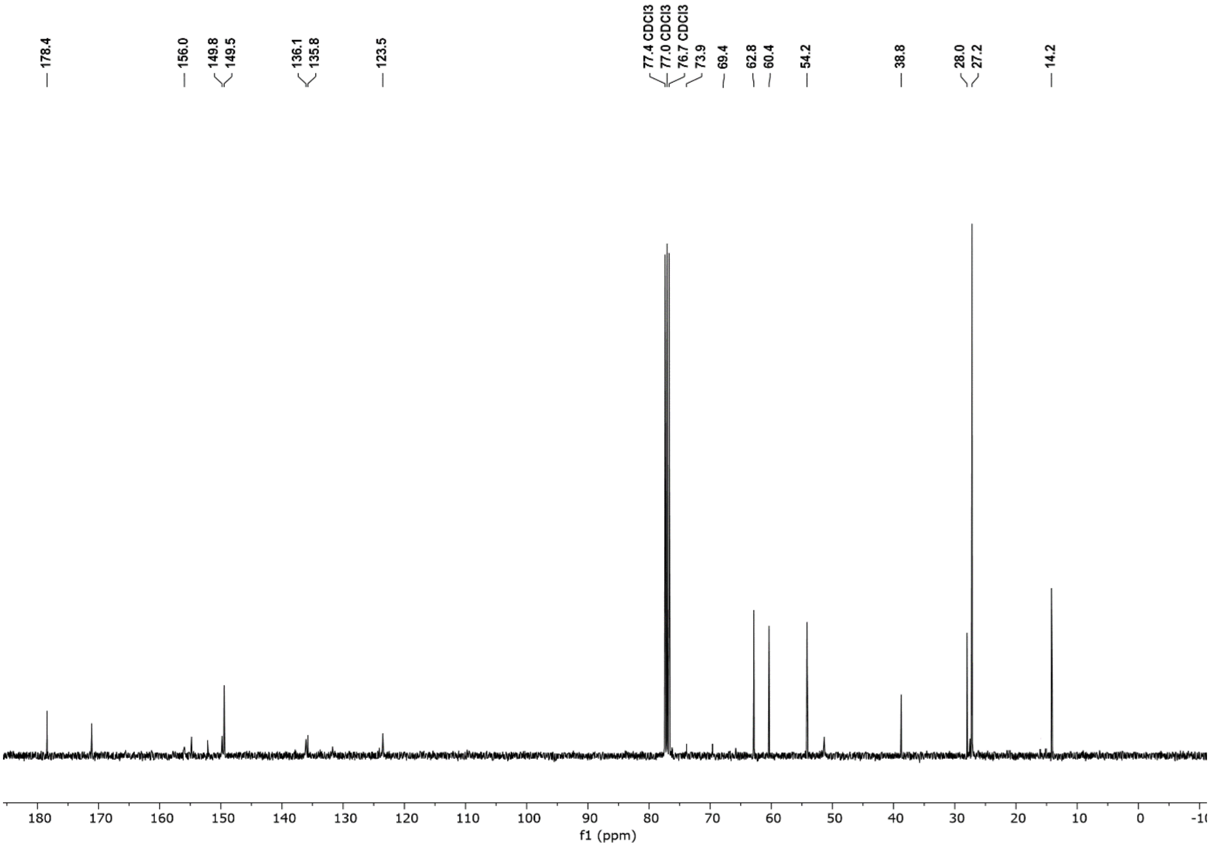

^{1}H NMR (400 MHz, $CDCl_3$) for **3q**

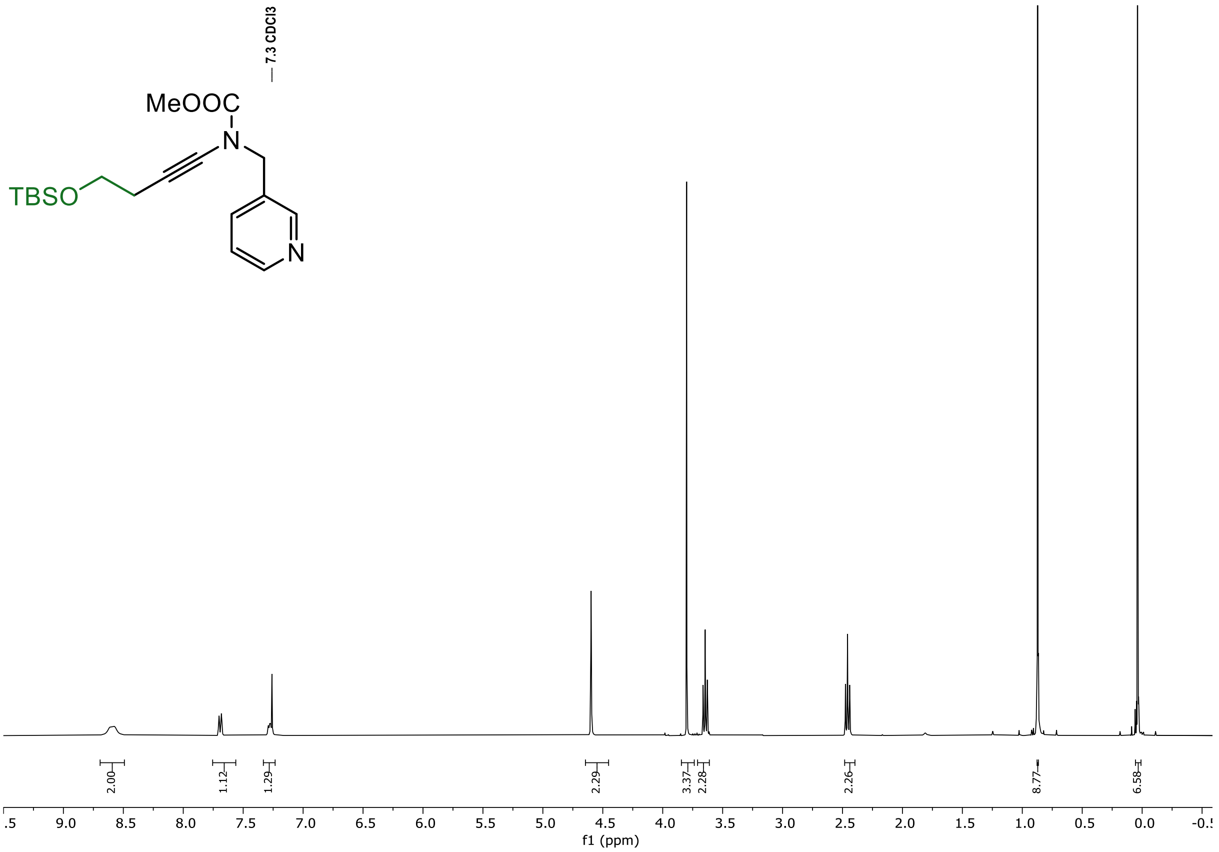

^{13}C NMR (101 MHz, $CDCl_3$) for **3q**

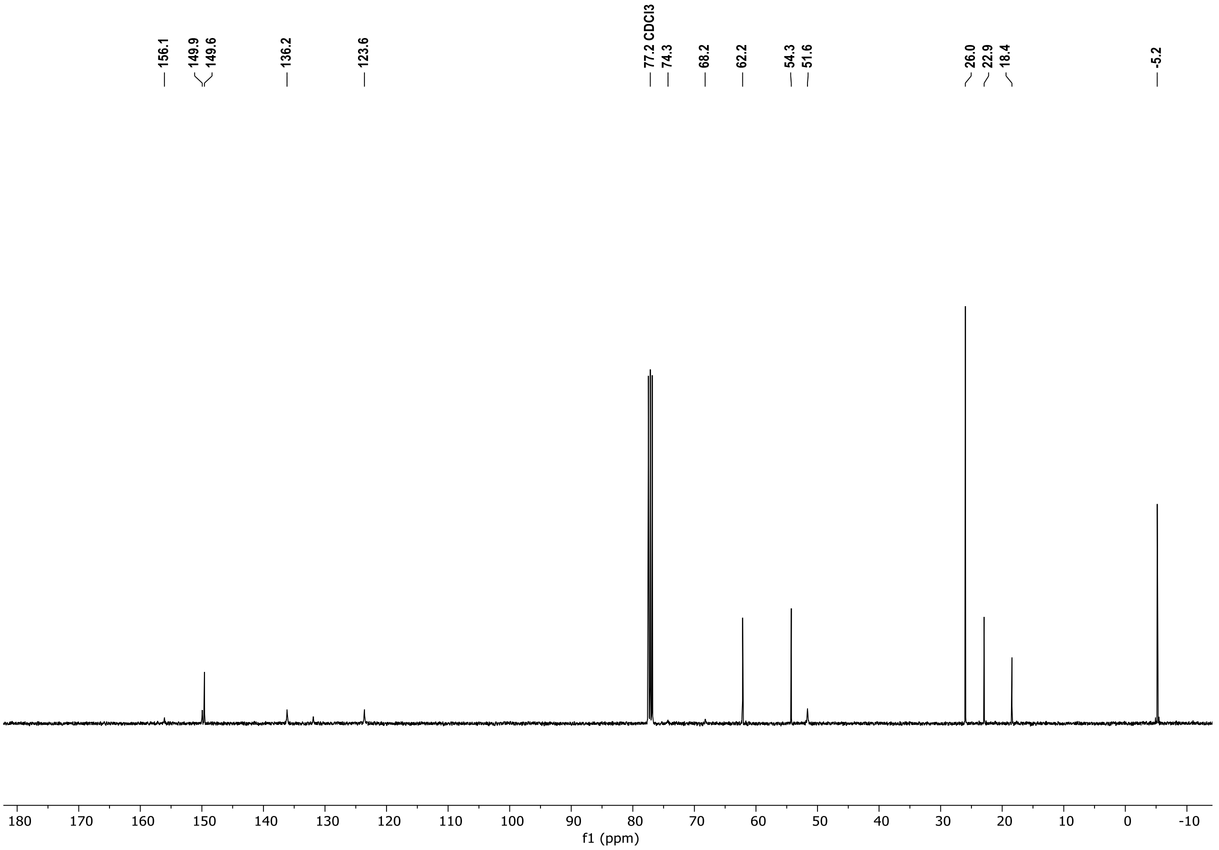

^{1}H NMR (400 MHz, $CDCl_3$) for **3r**

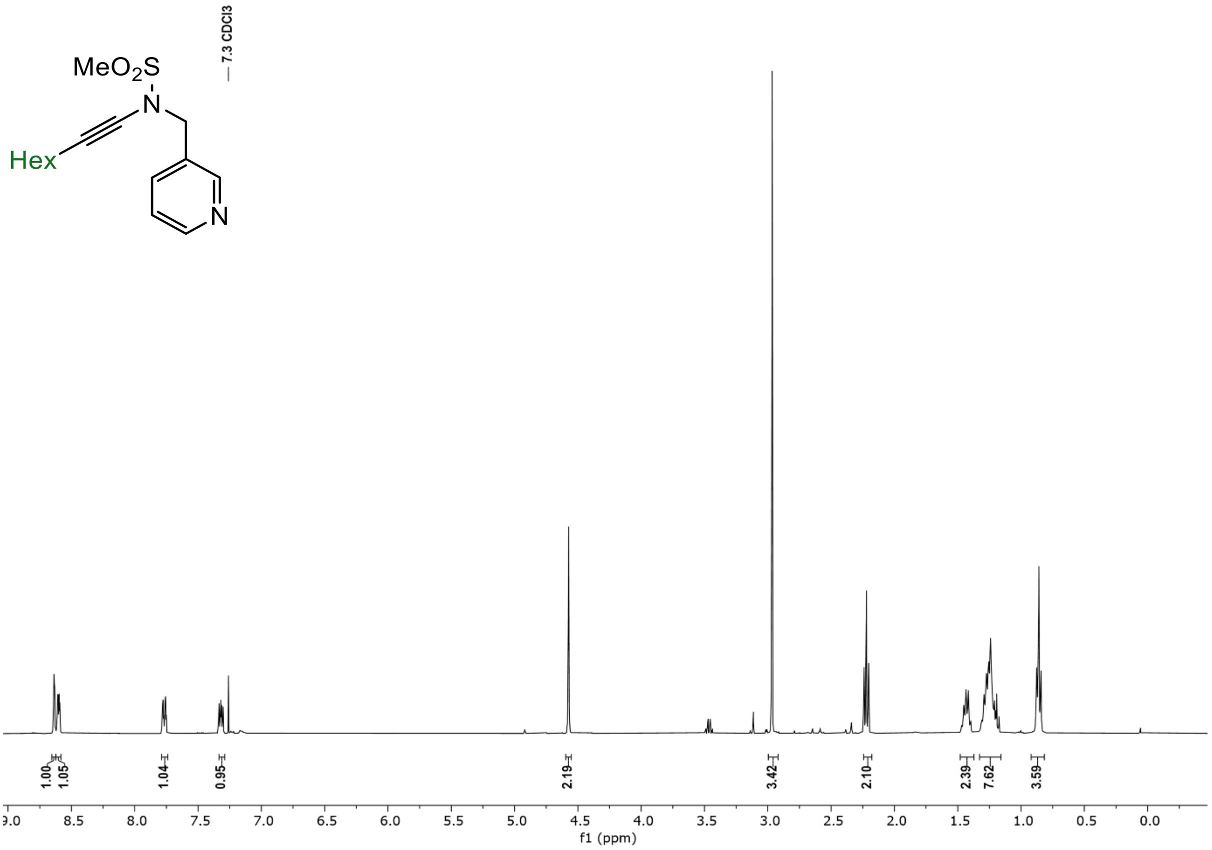

^{13}C NMR (101 MHz, $CDCl_3$) for **3r**

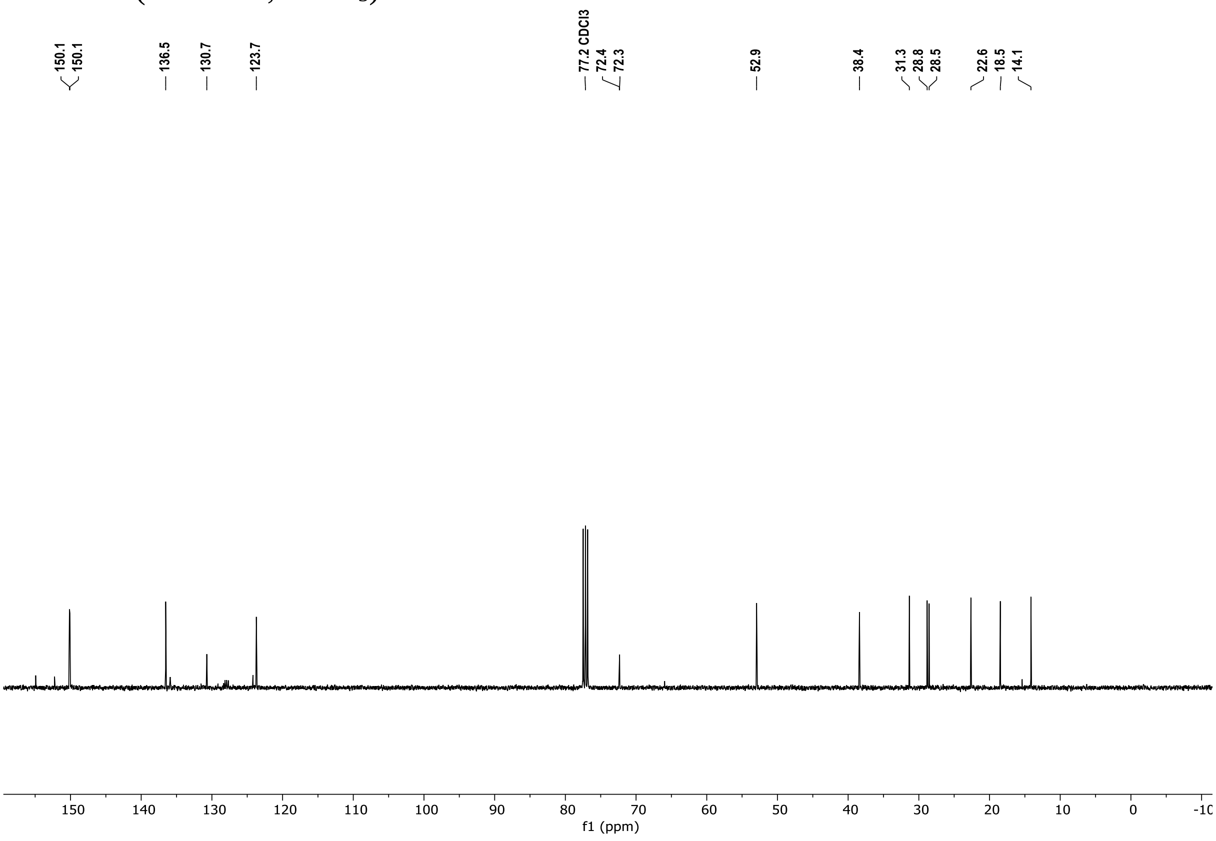

^{1}H NMR (400 MHz, $CDCl_3$) for **3s**

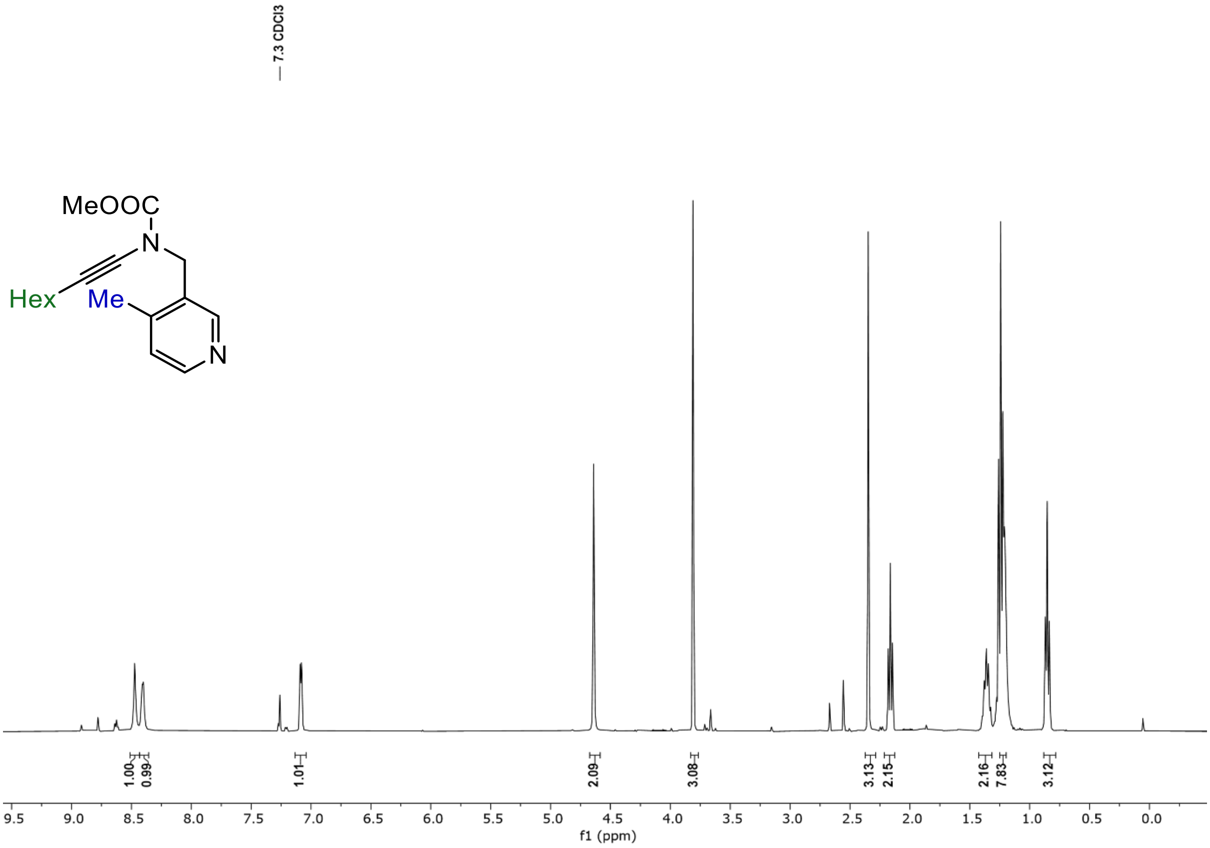

^{13}C NMR (101 MHz, $CDCl_3$) for **3s**

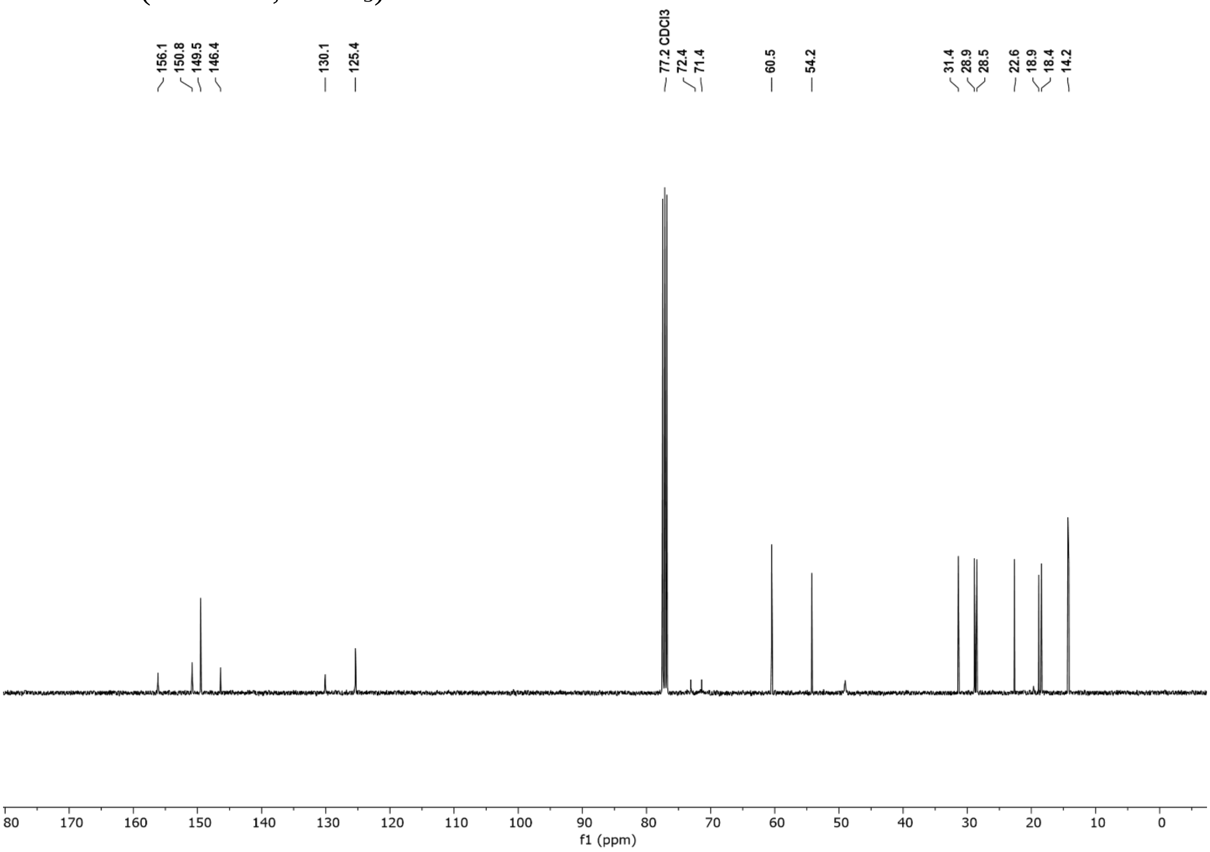

^{1}H NMR (400 MHz, $CDCl_3$) for **6a**

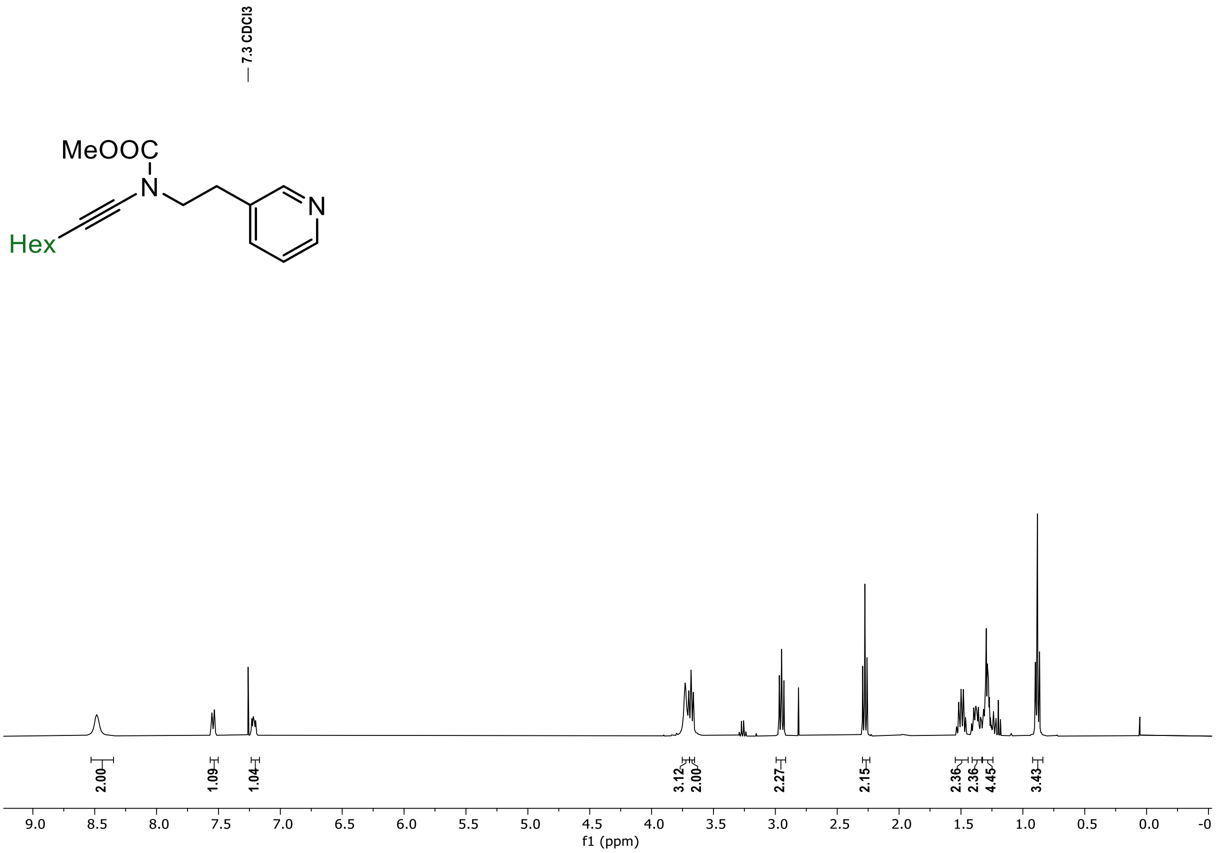

^{13}C NMR (101 MHz, $CDCl_3$) for **6a**

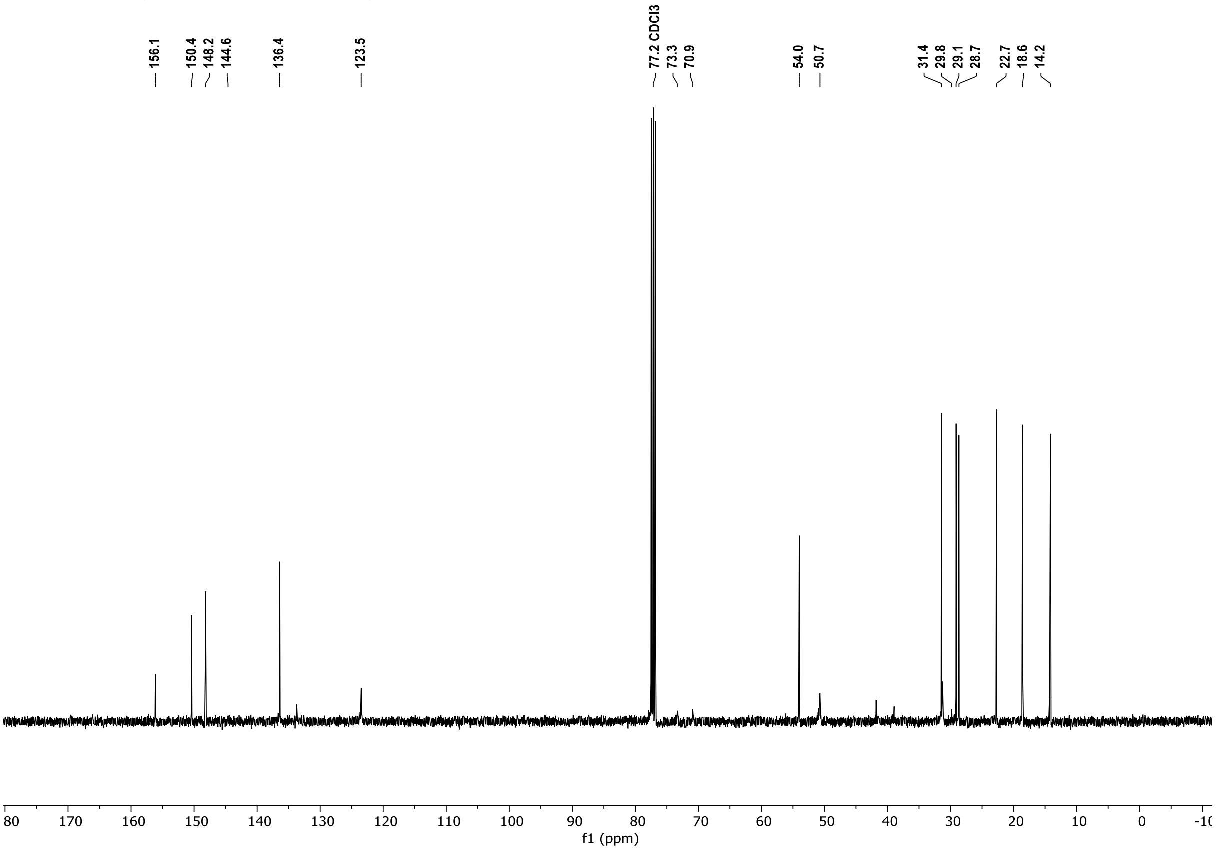

^{1}H NMR (400 MHz, $CDCl_3$) for **6e**

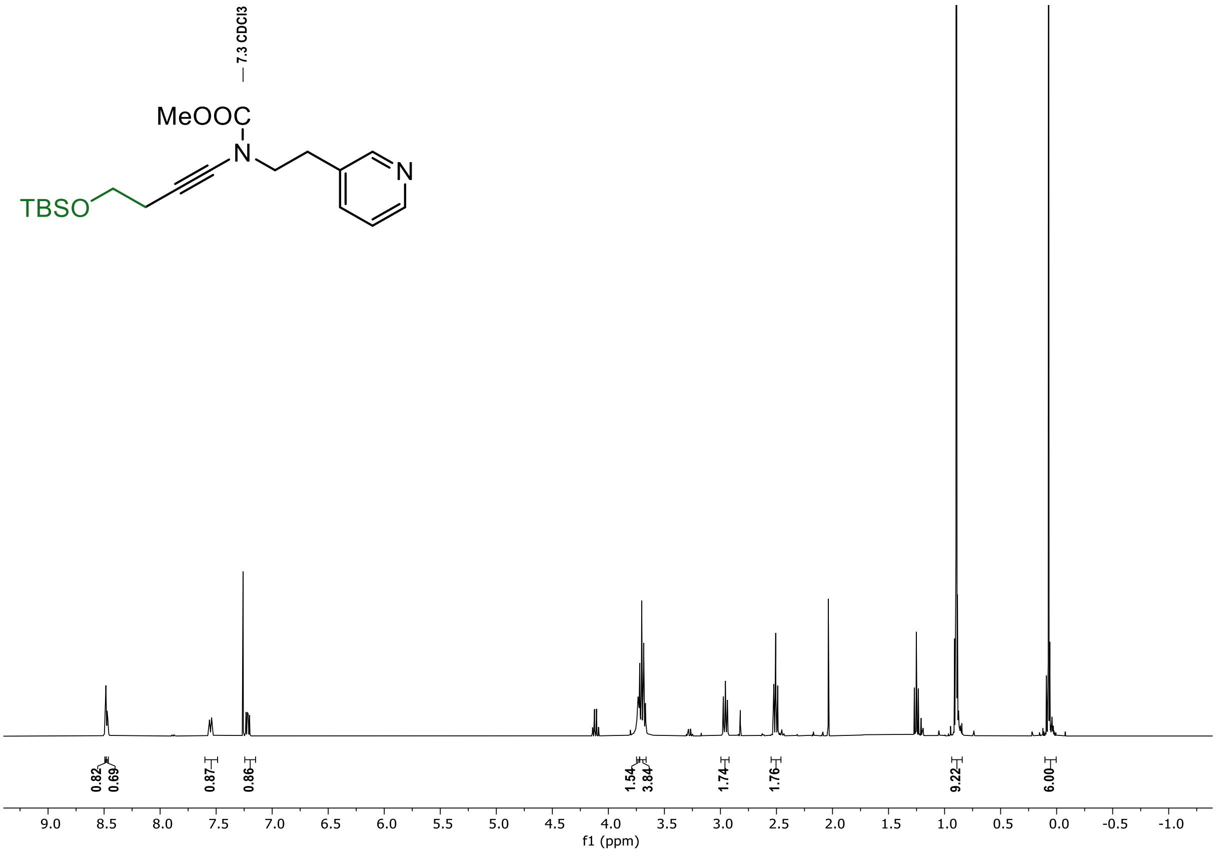

^{13}C NMR (101 MHz, $CDCl_3$) for **6e**

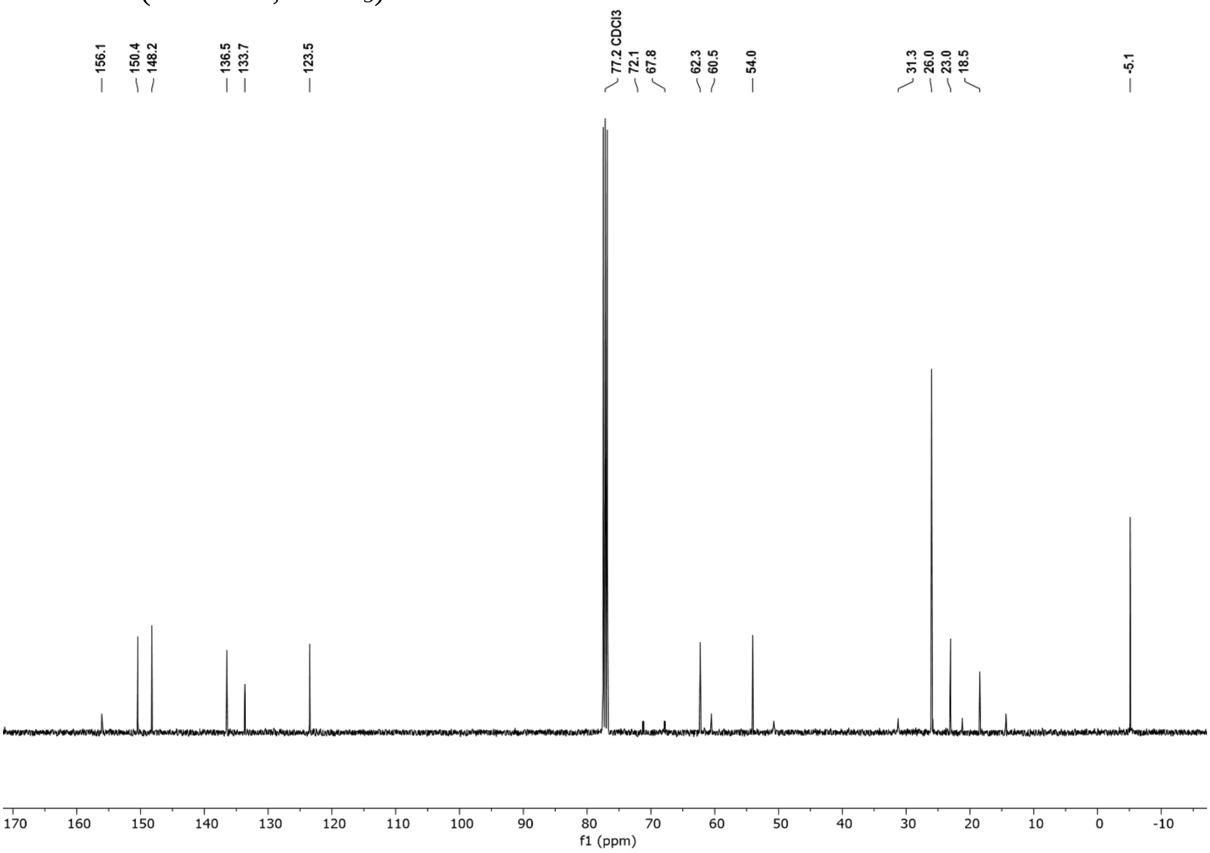

^{1}H NMR (400 MHz, $CDCl_3$) for **4a**

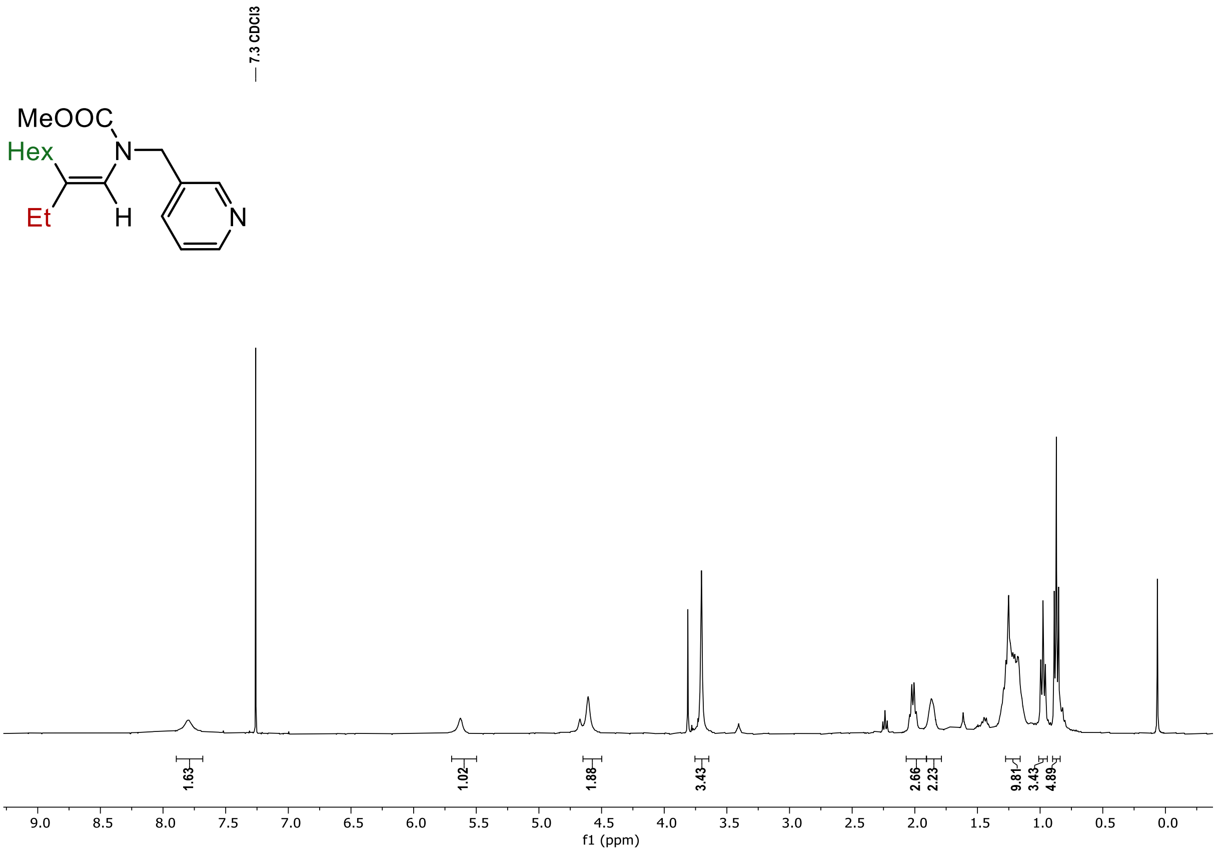

^{13}C NMR (126 MHz, $CDCl_3$) for **4a**

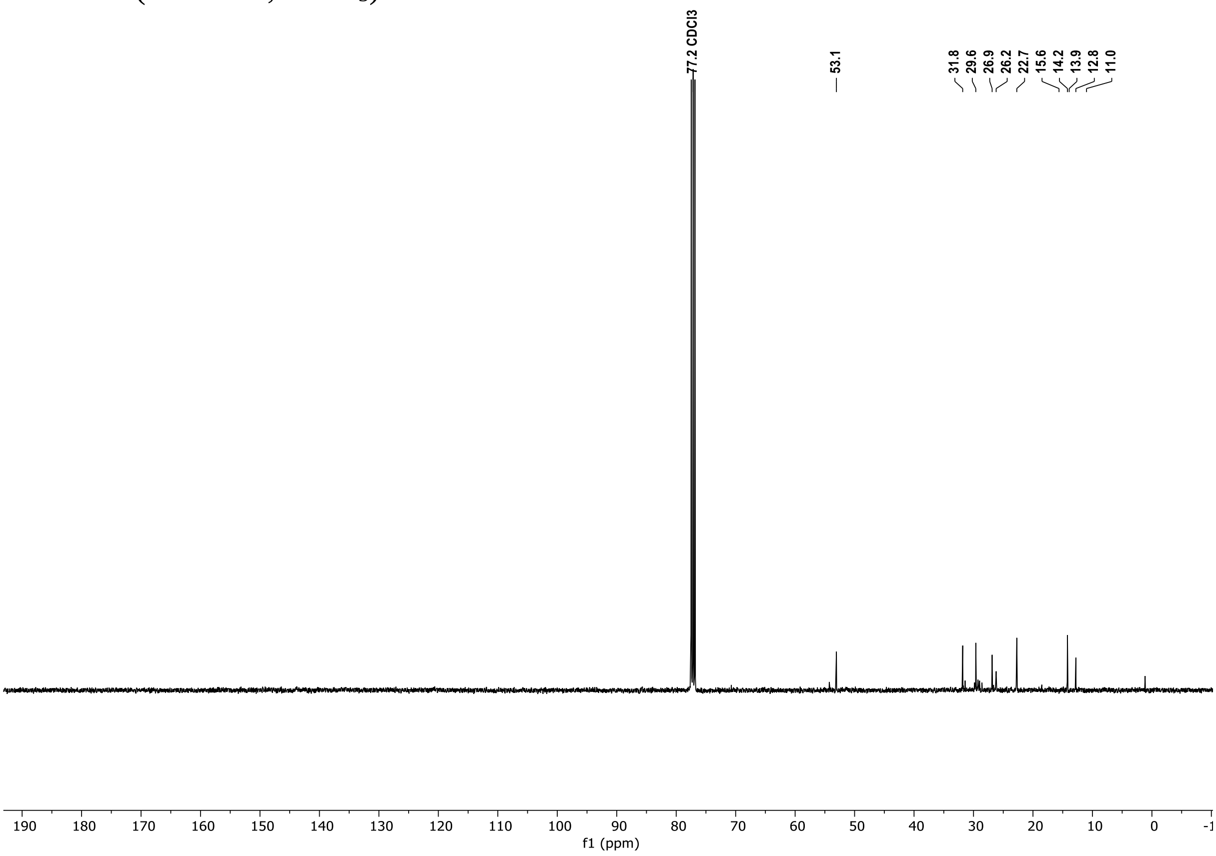

^{1}H NMR (400 MHz, $CDCl_3$) for **4f**

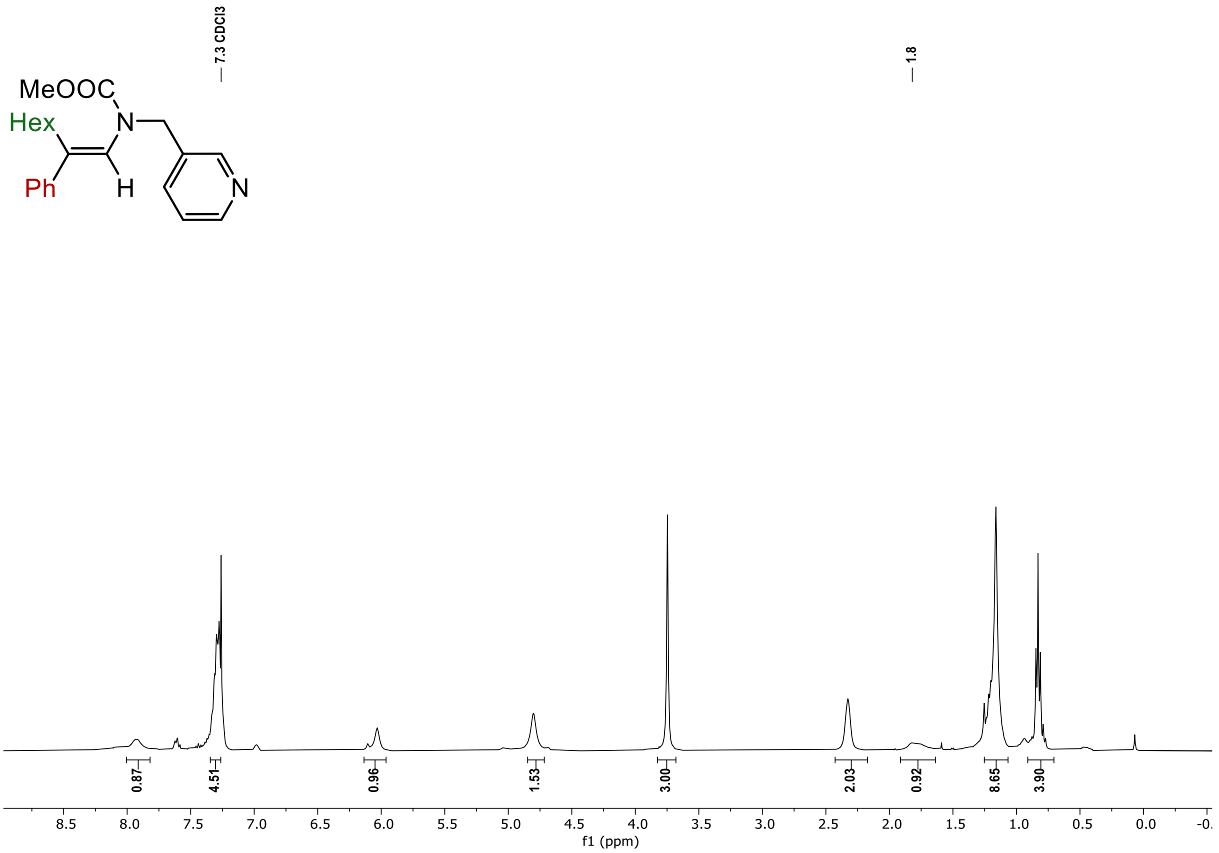

^{13}C NMR (126 MHz, $CDCl_3$) for **4f**

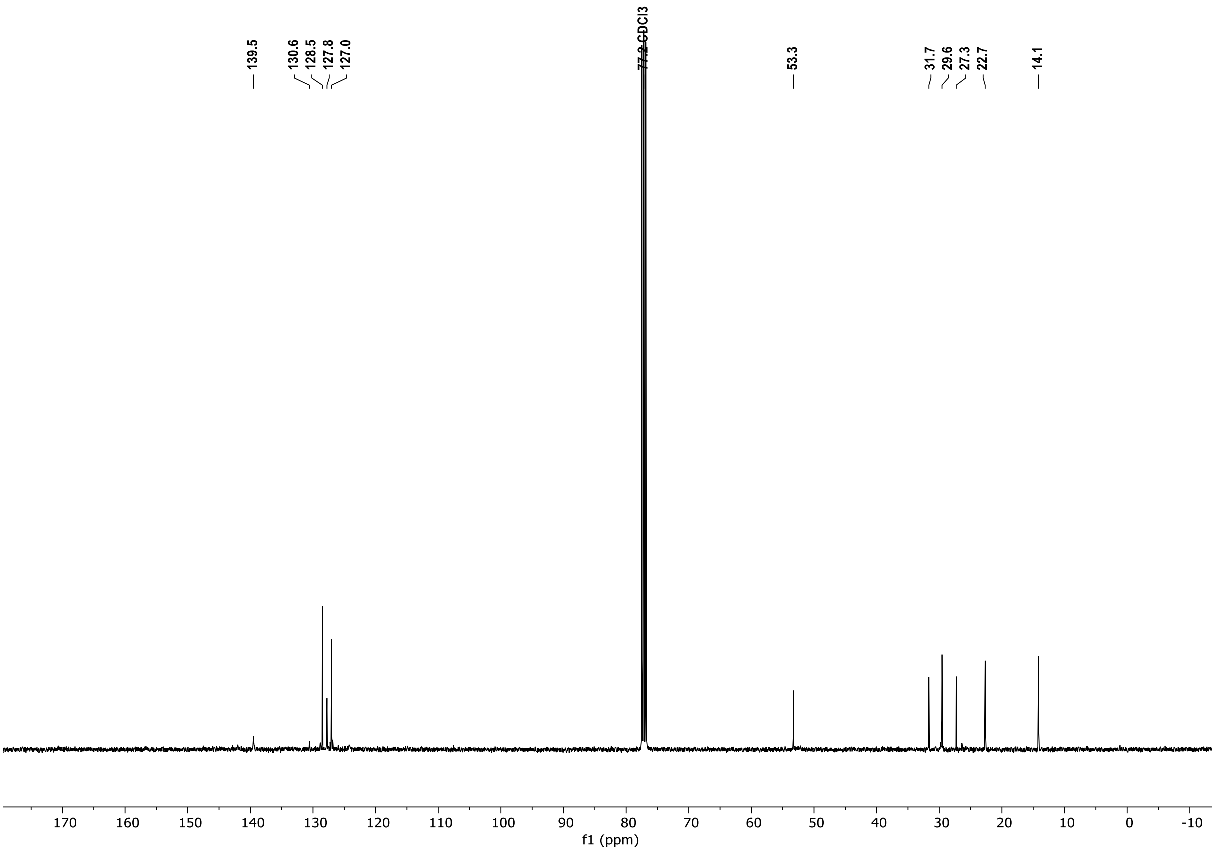

^{1}H NMR (400 MHz, C_6D_6) for **5a**

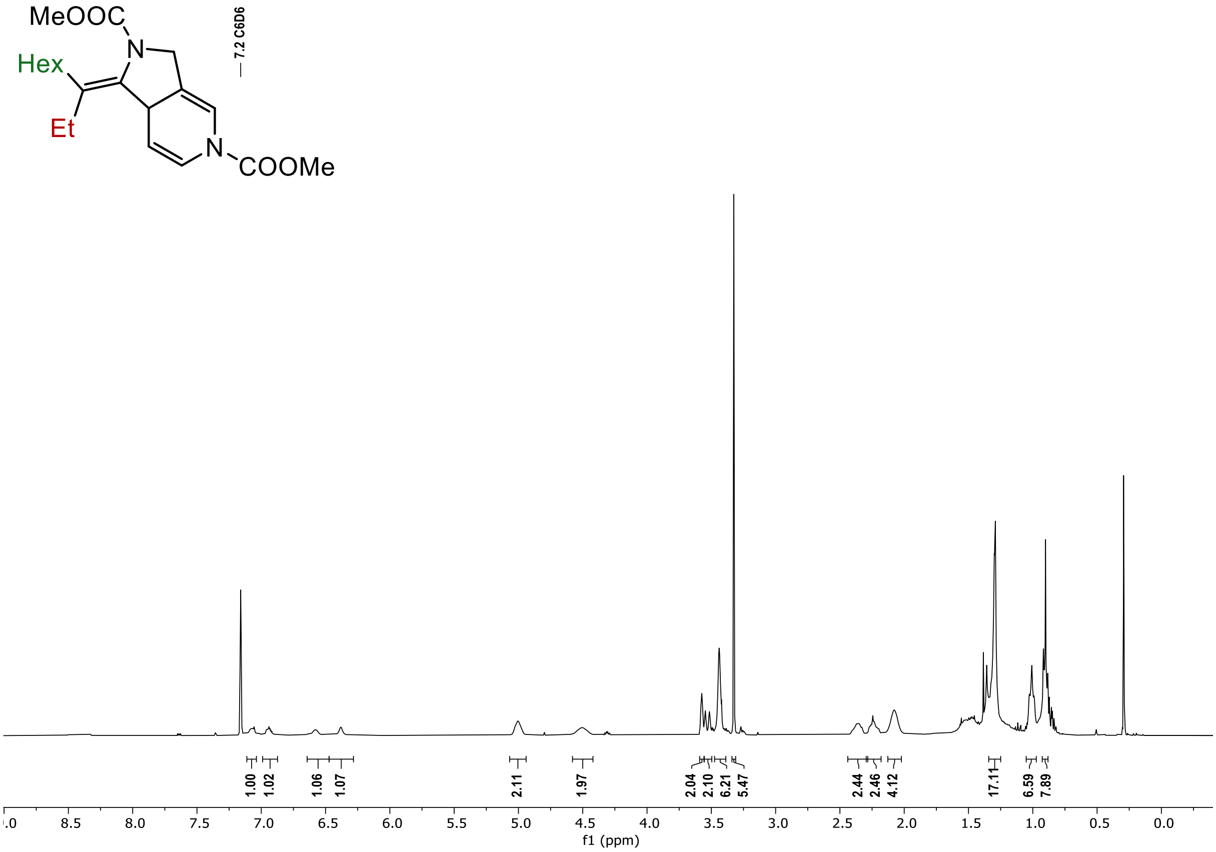

^{13}C NMR (126 MHz, C_6D_6) for **5a**

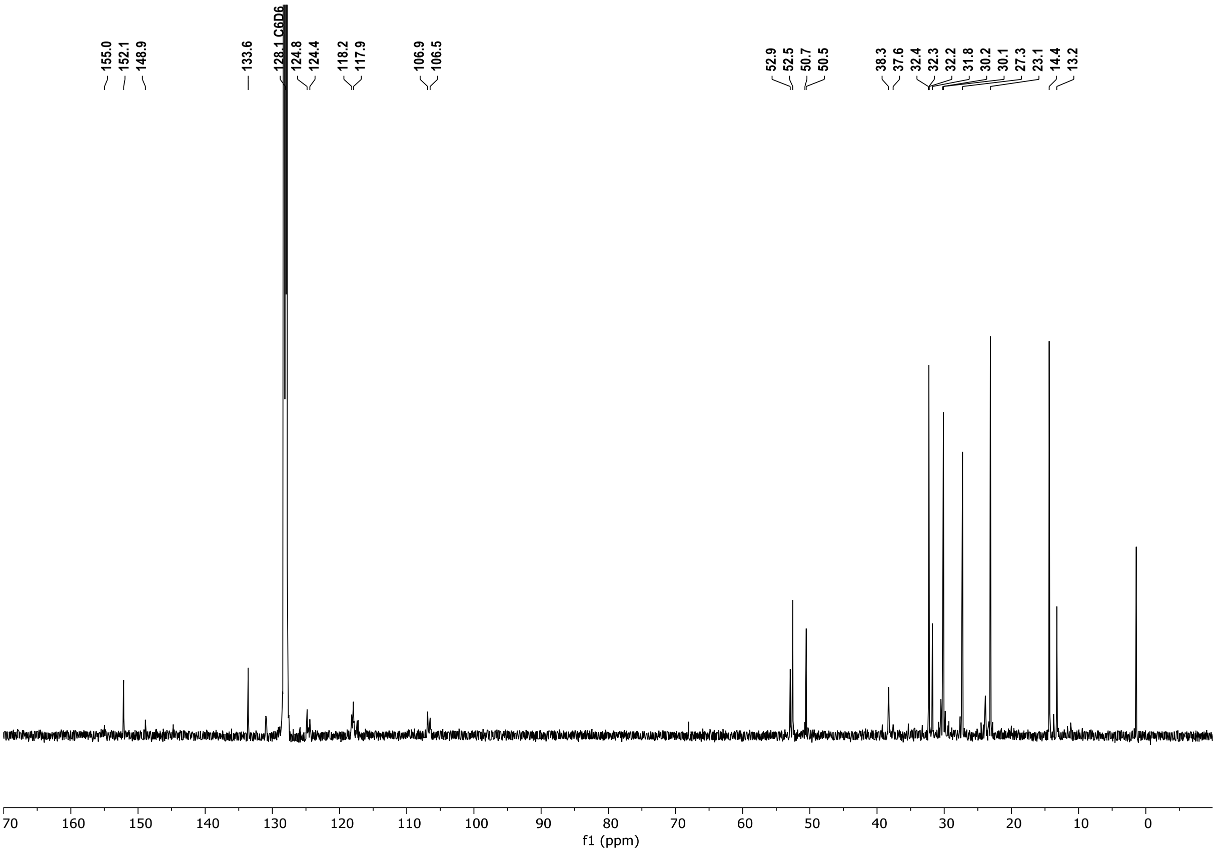

^{1}H NMR (500 MHz, C_6D_6) for **5b**

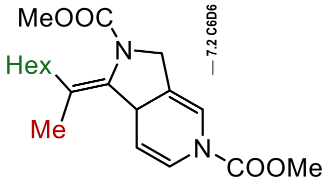

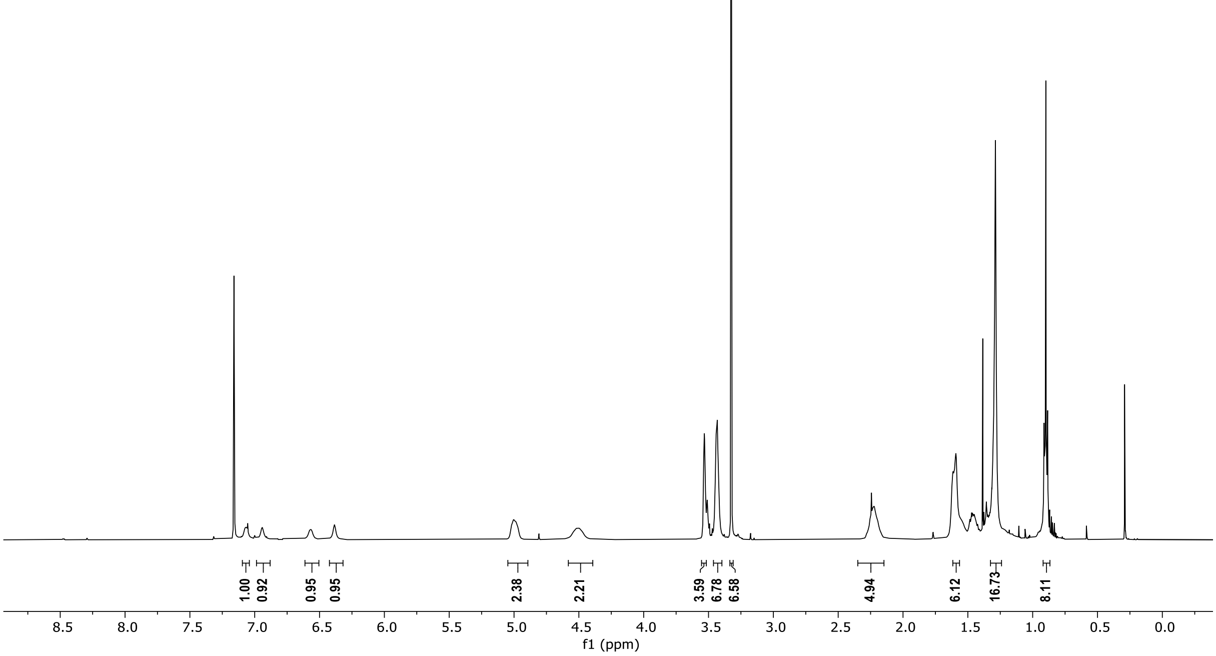

^{13}C NMR (126 MHz, C_6D_6) for **5b**

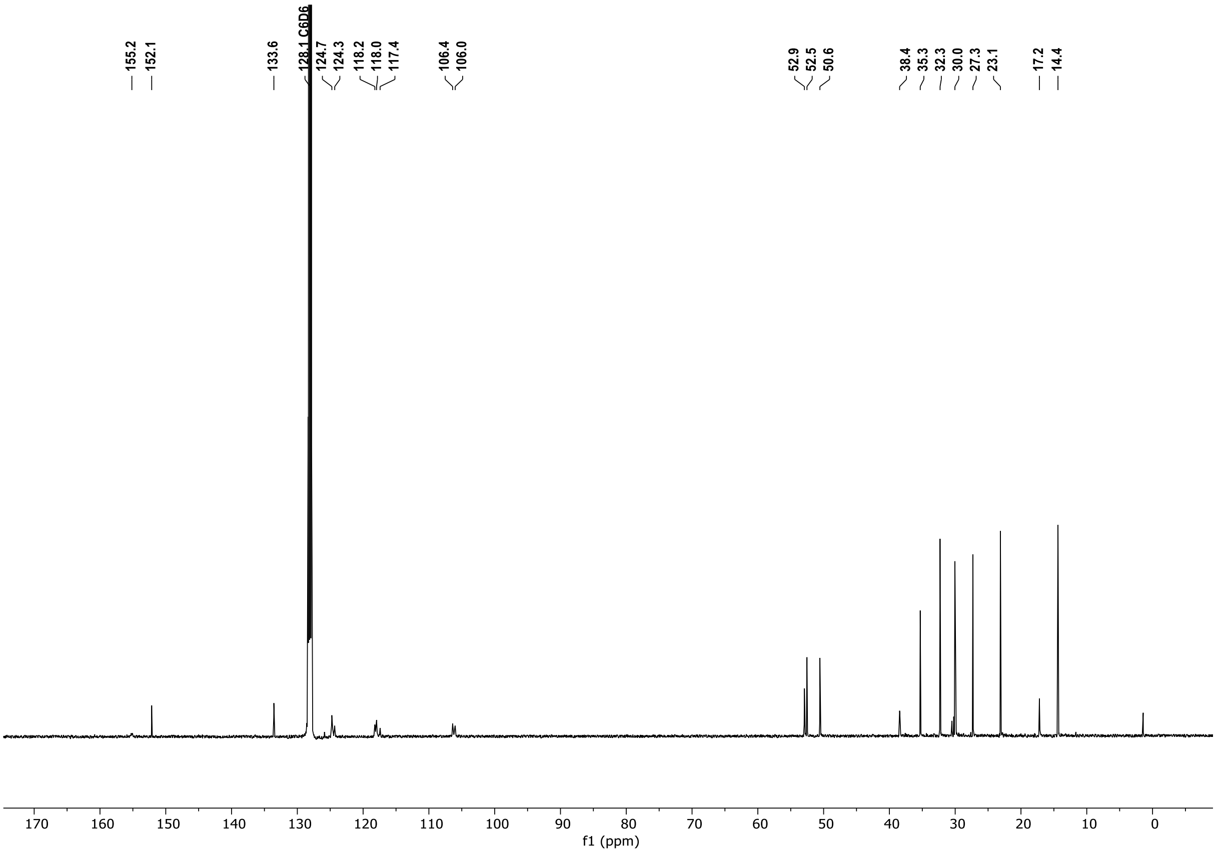

^{1}H NMR (500 MHz, C_6D_6) for **5c**

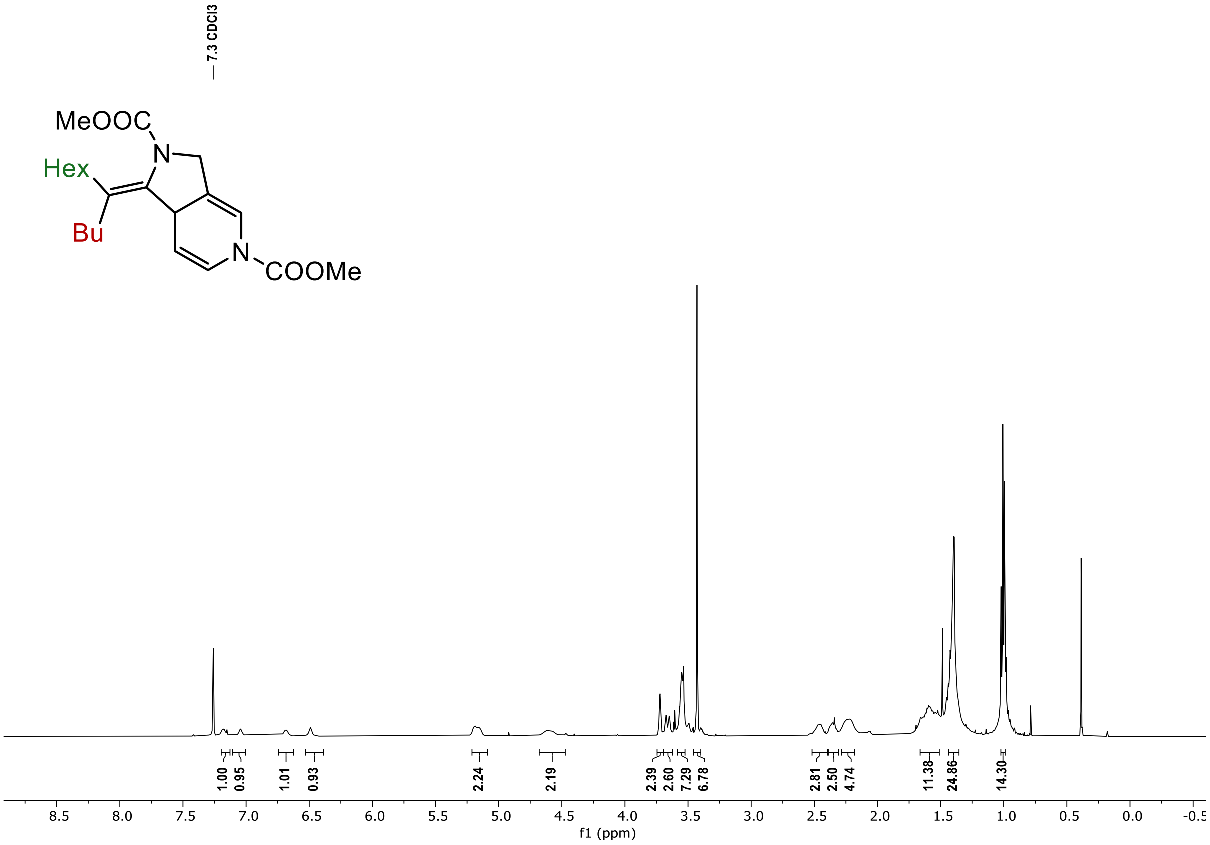

^{13}C NMR (126 MHz, C_6D_6) for **5c**

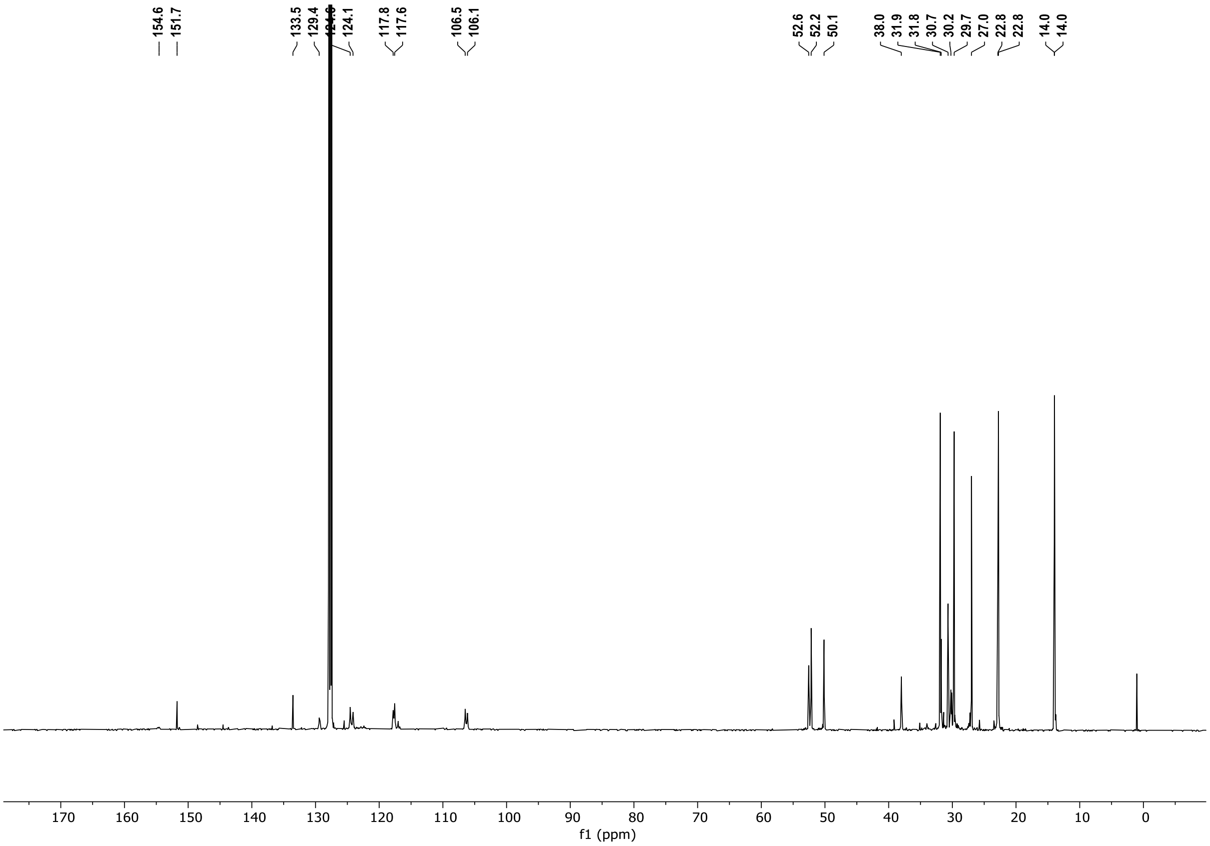

^{1}H NMR (400 MHz, C_6D_6) for **5d**

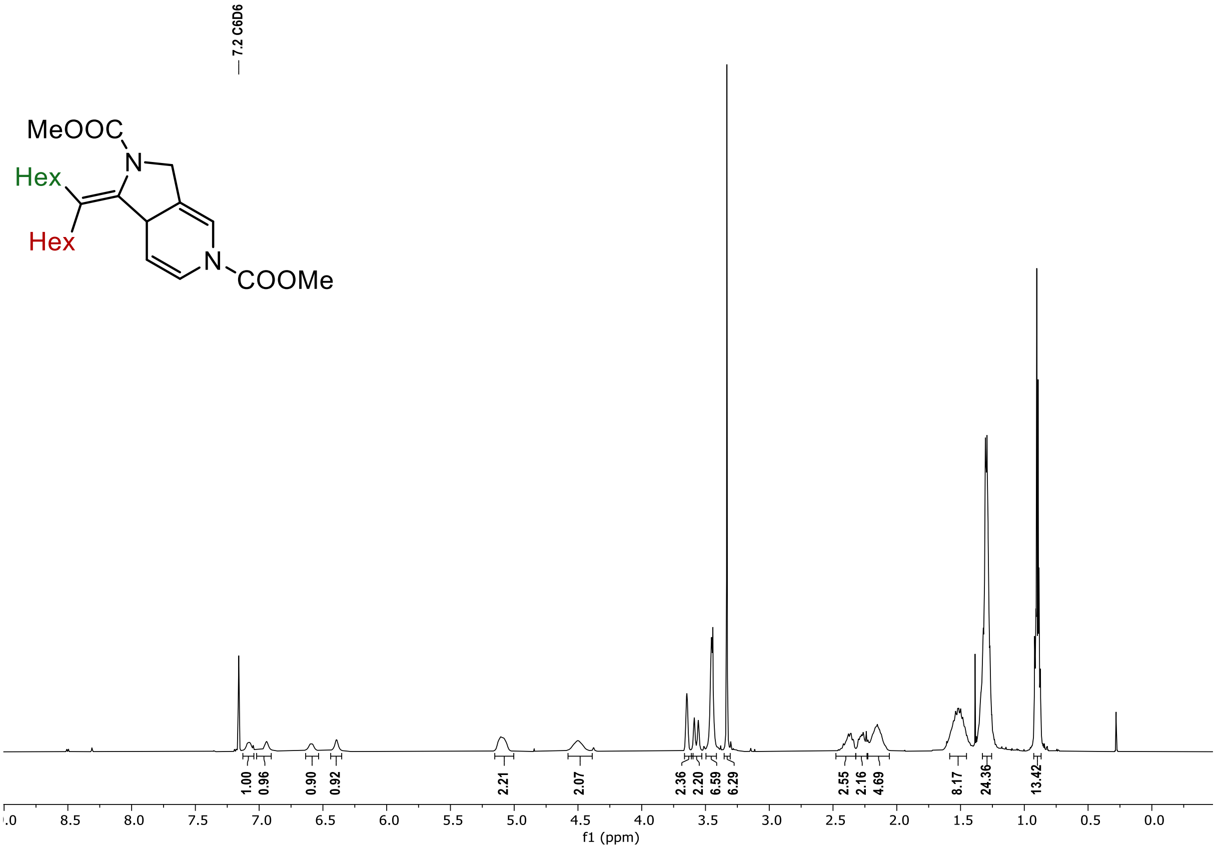

^{13}C NMR (101 MHz, C_6D_6) for **5d**

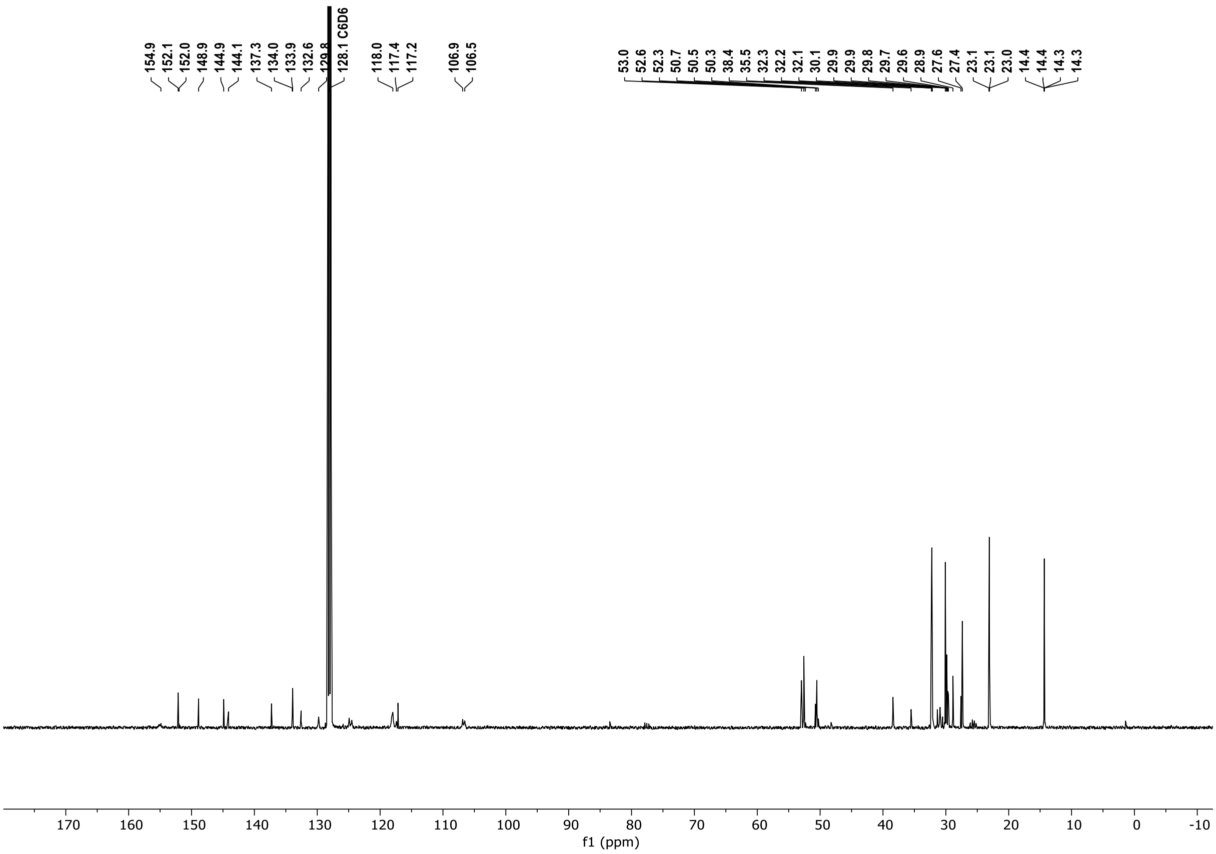

^{1}H NMR (500 MHz, C_6D_6) for **5e**

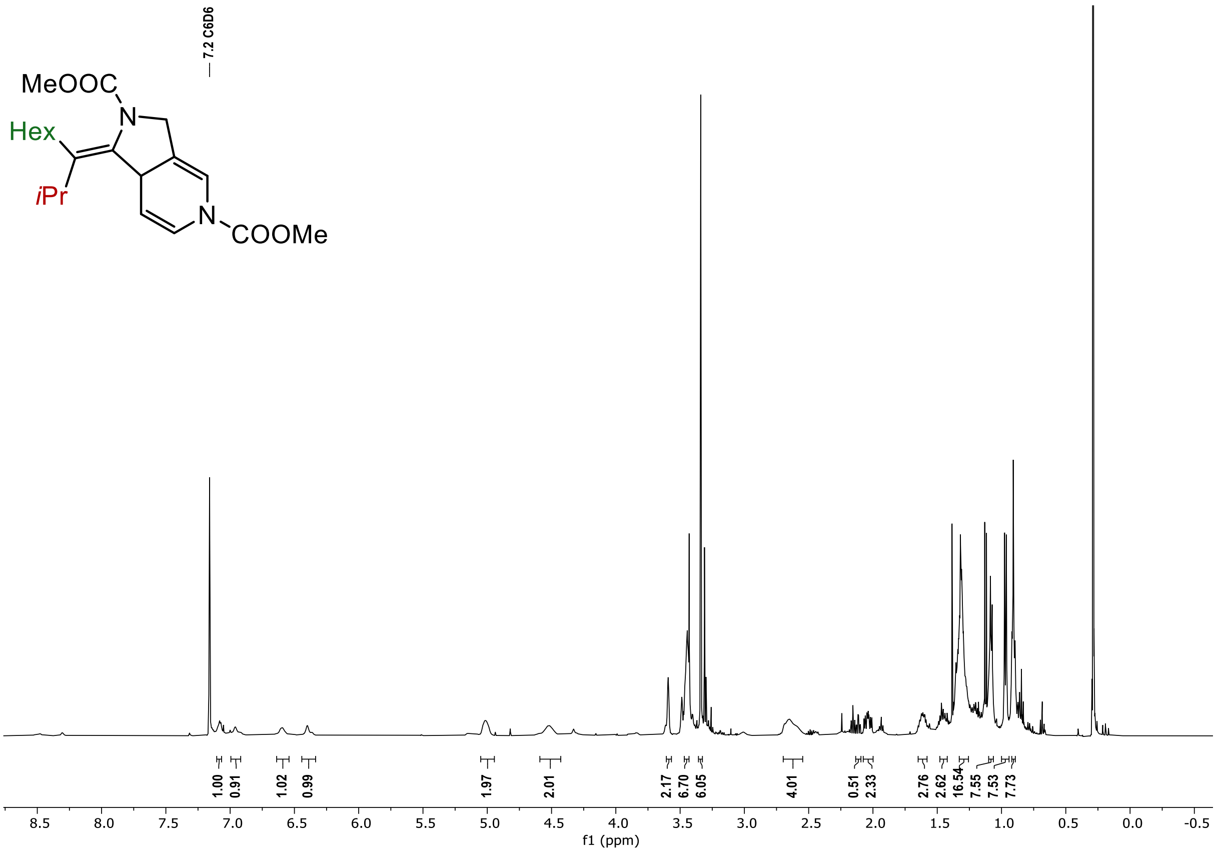

^{13}C NMR (126 MHz, C_6D_6) for **5e**

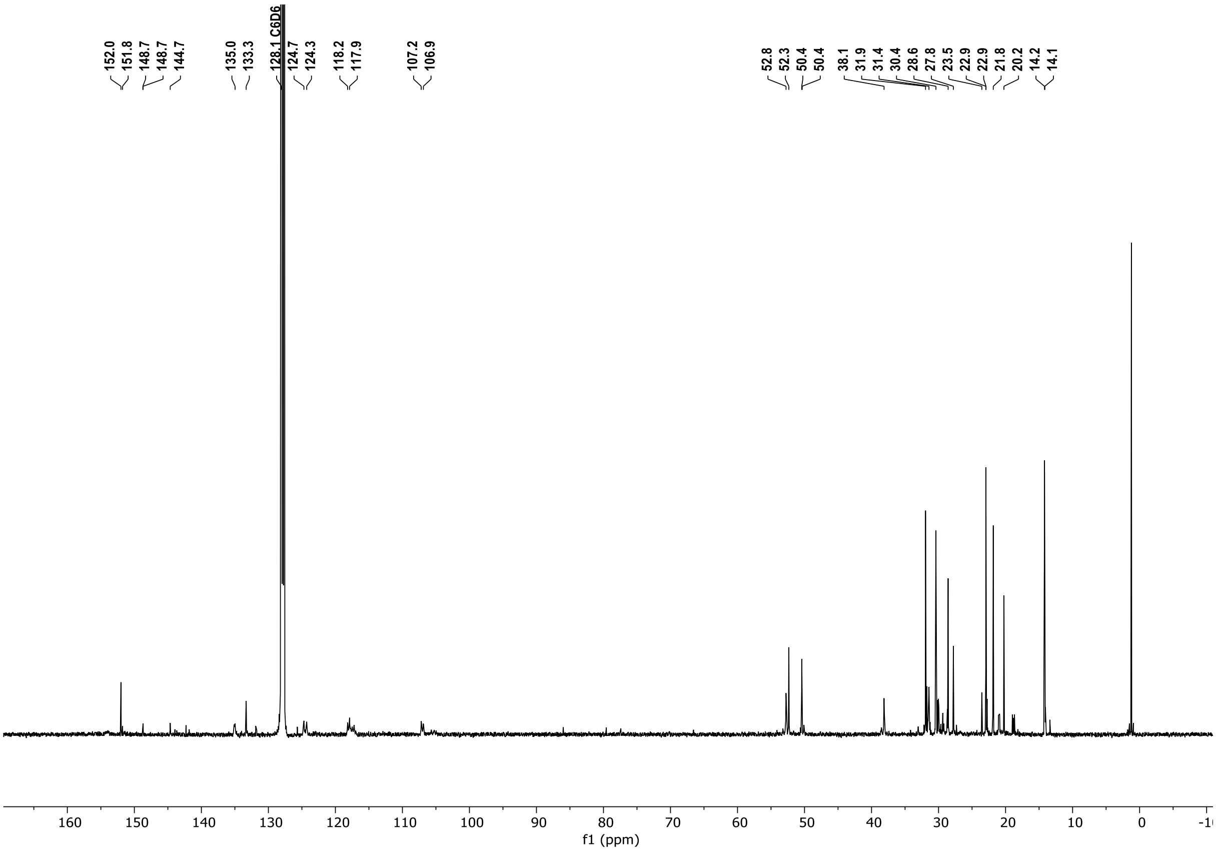

^{1}H NMR (400 MHz, C_6D_6) for **5f**

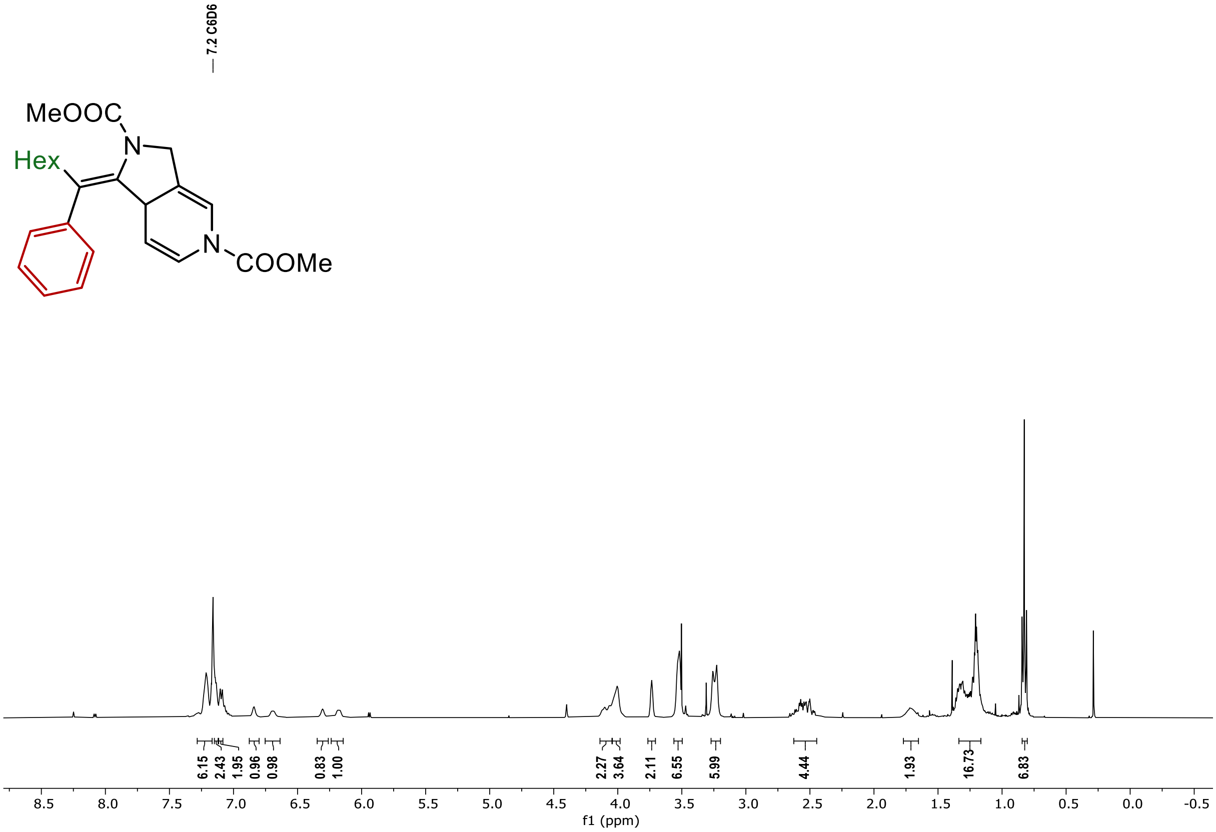

^{13}C NMR (101 MHz, C_6D_6) for **5f**

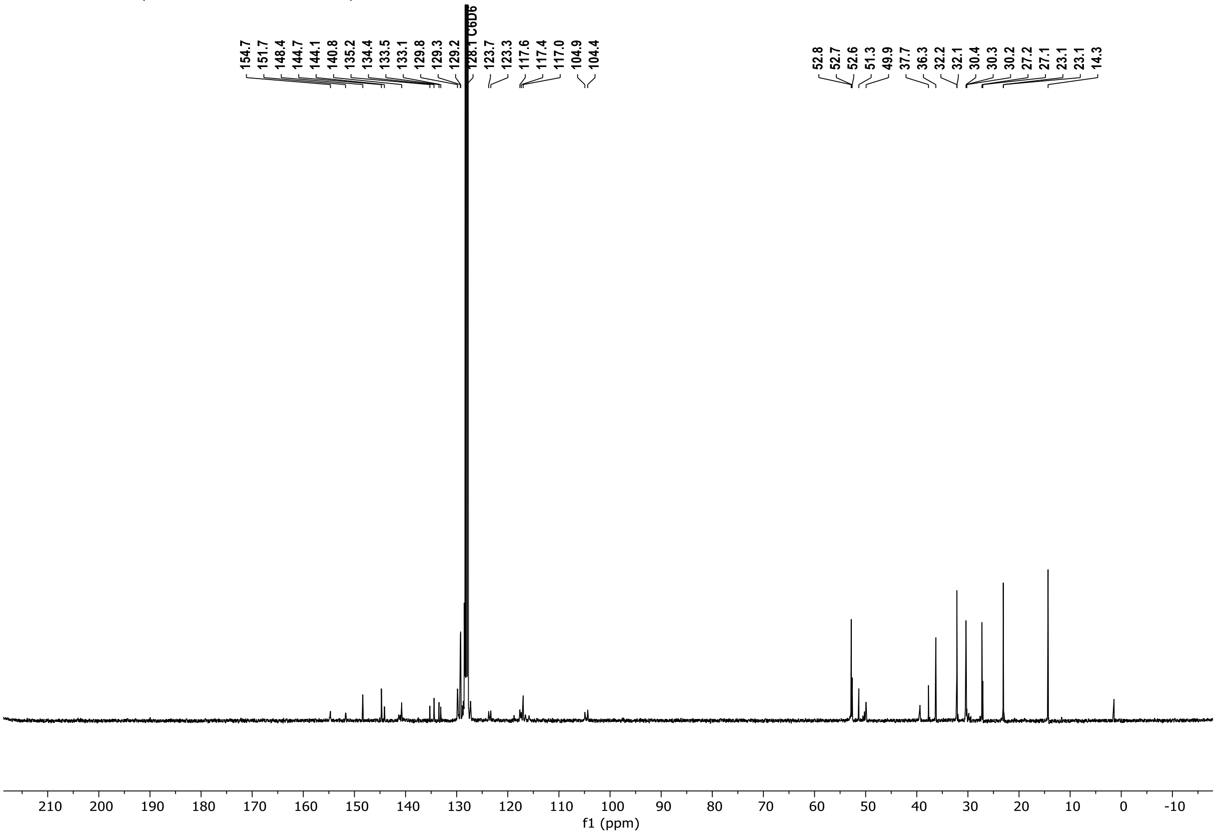

^{1}H NMR (400 MHz, C_6D_6) for **5h**

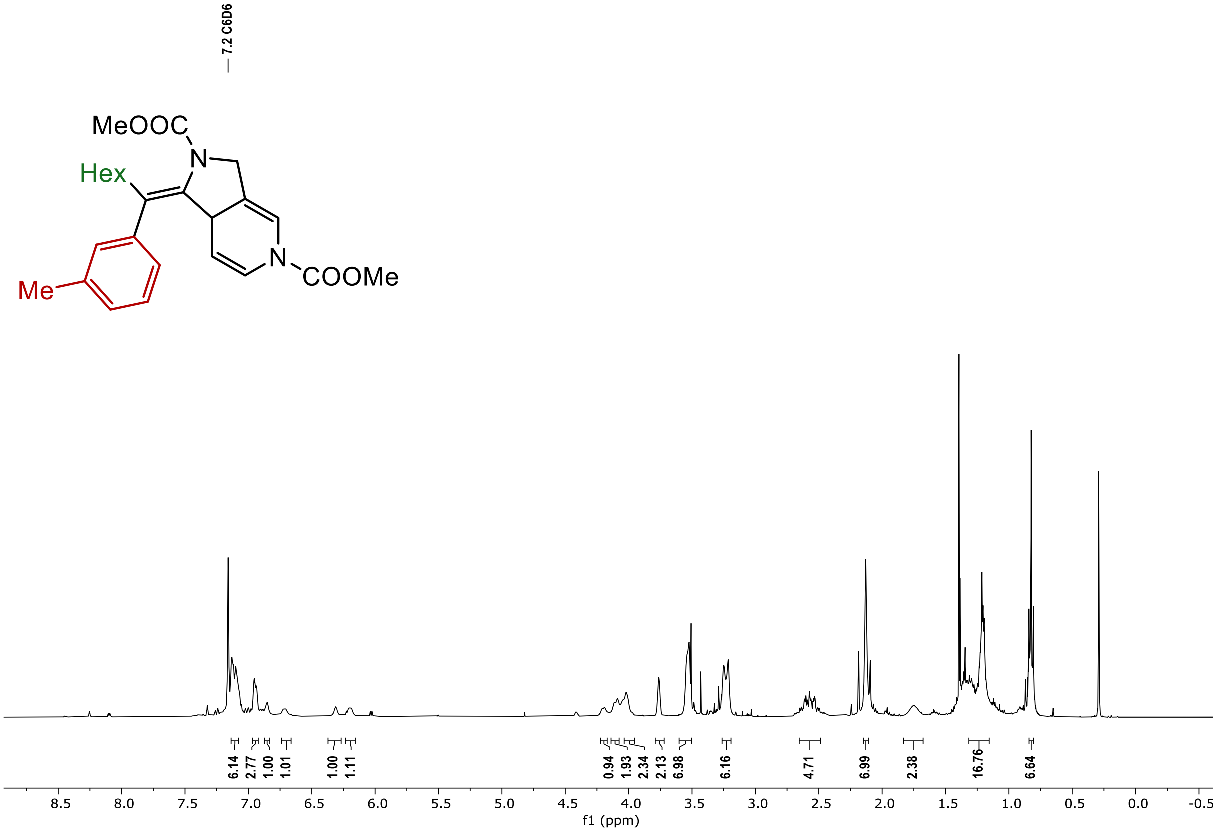

^{13}C NMR (101 MHz, C_6D_6) for **5h**

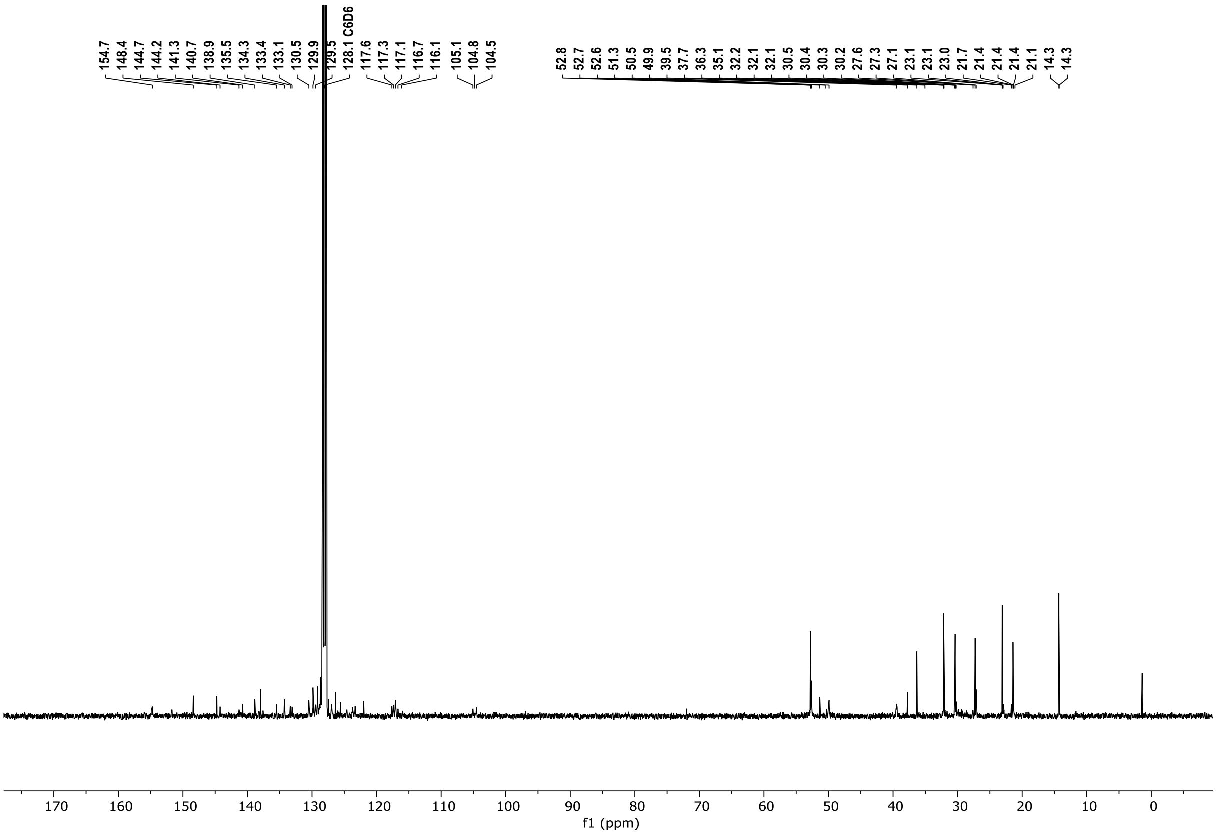

^{1}H NMR (400 MHz, C_6D_6) for **5i**

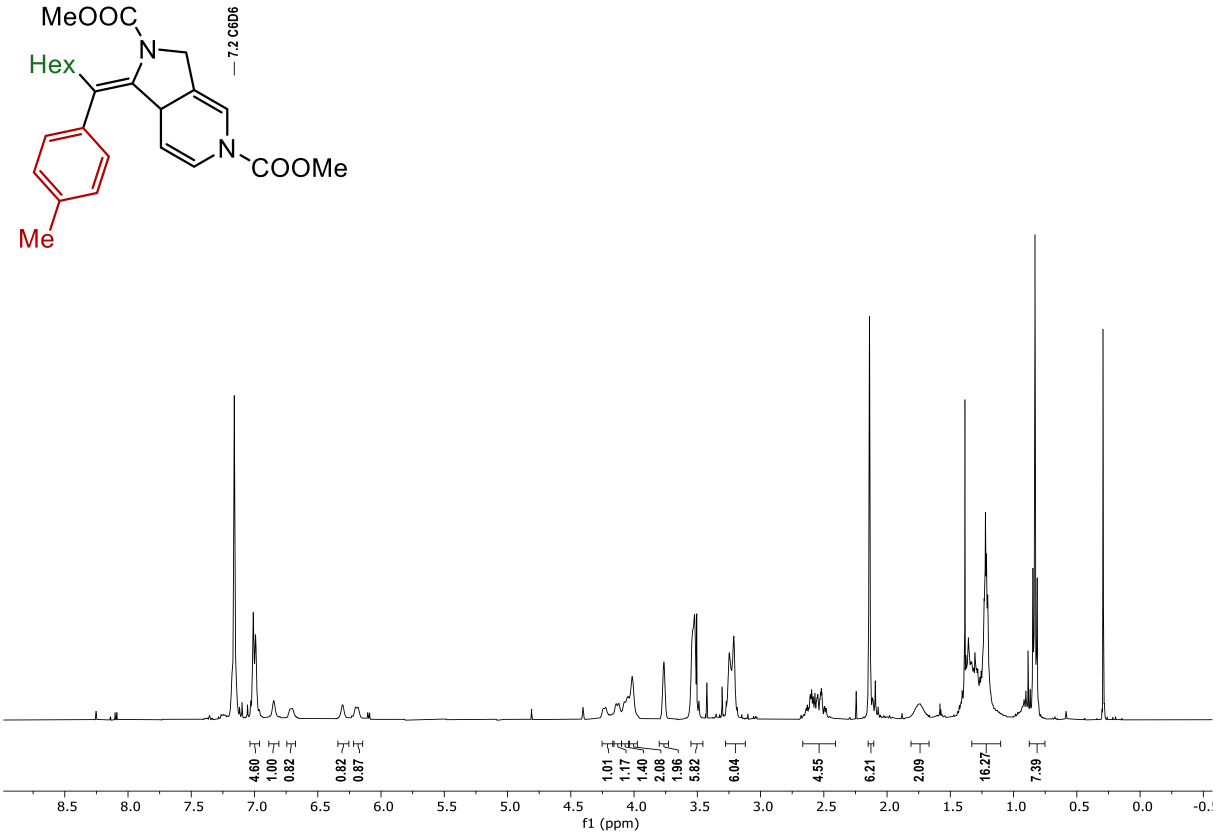

^{13}C NMR (101 MHz, C_6D_6) for **5i**

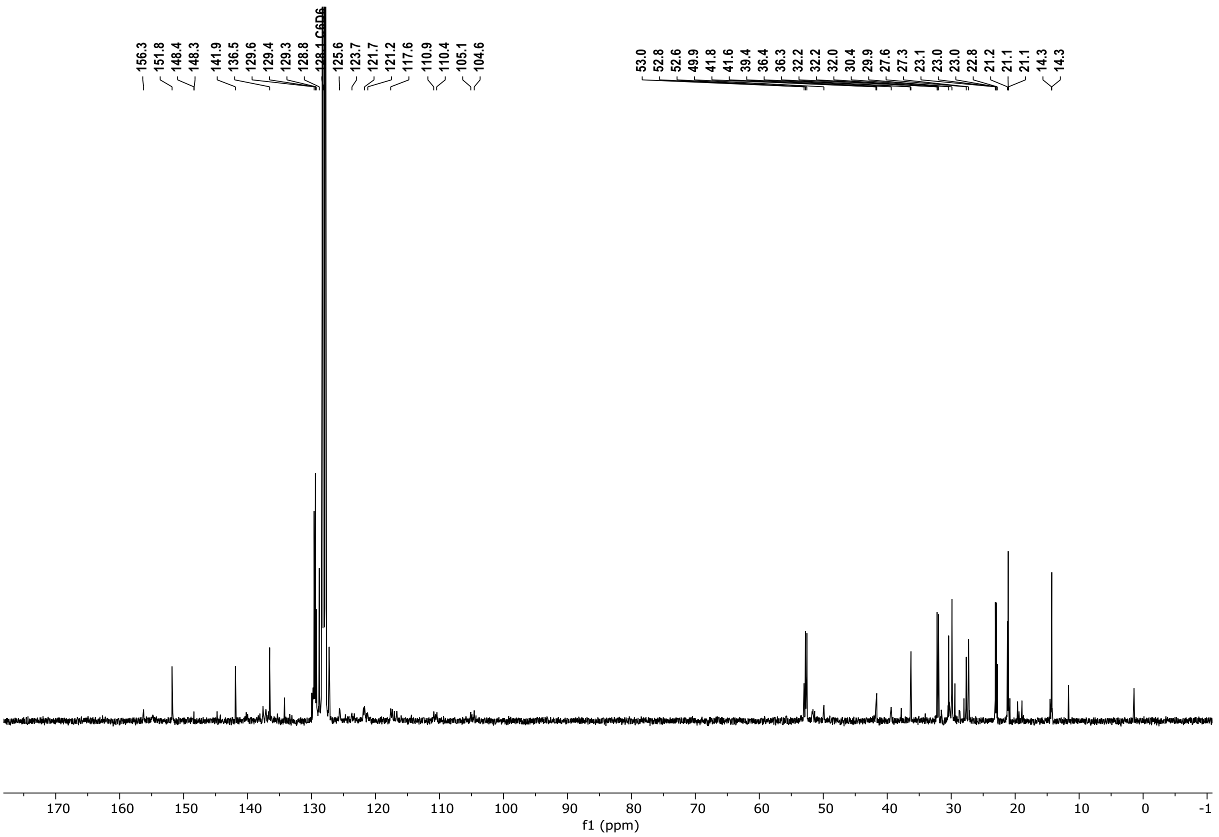

^{1}H NMR (500 MHz, C_6D_6) for **5j**

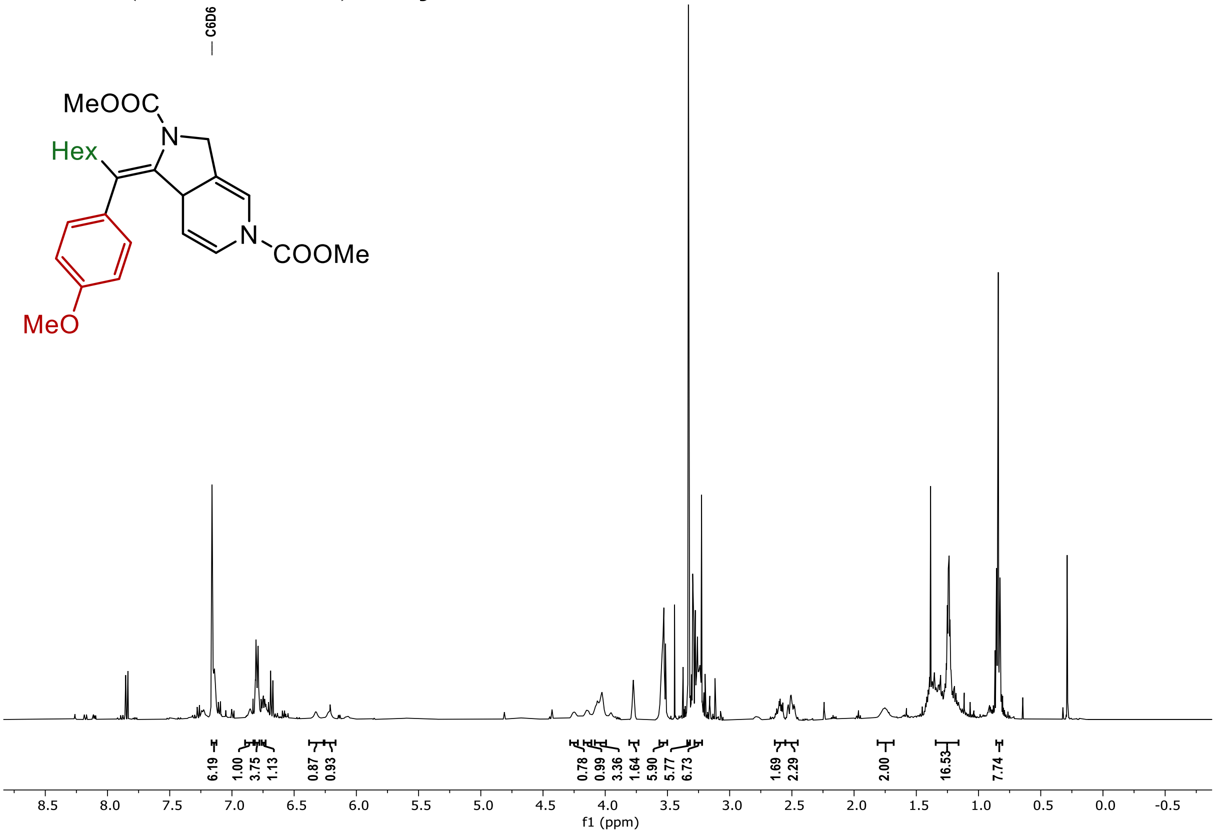

^{13}C NMR (126 MHz, C_6D_6) for **5j**

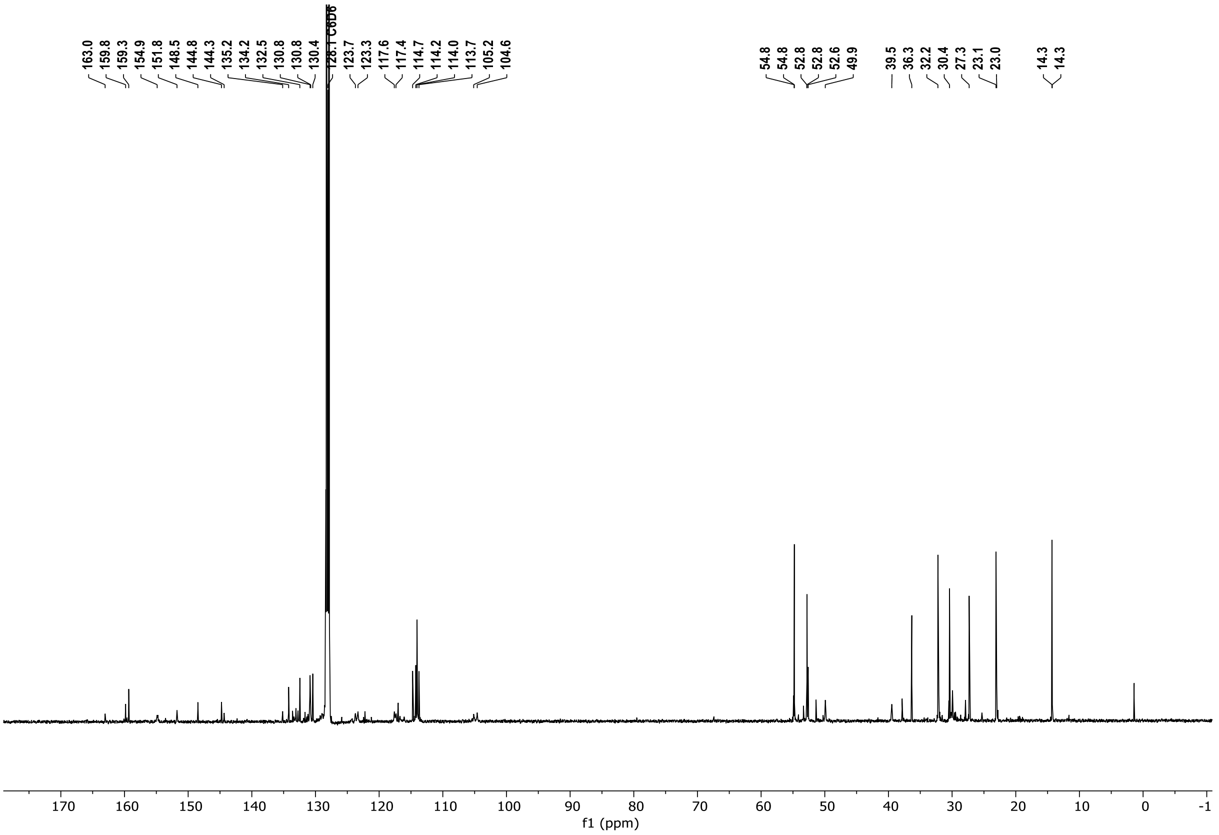

^{1}H NMR (500 MHz, C_6D_6) for **5k**

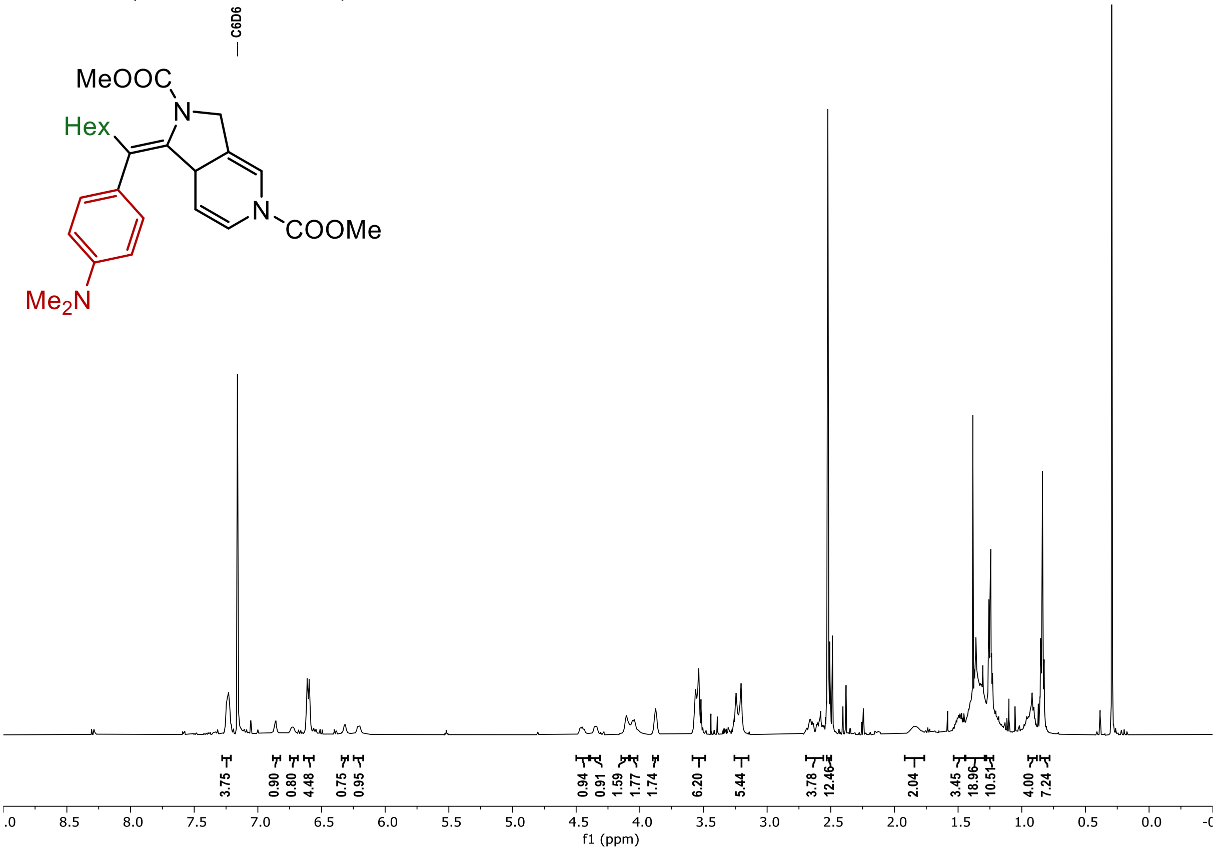

^{13}C NMR (126 MHz, C_6D_6) for **5k**

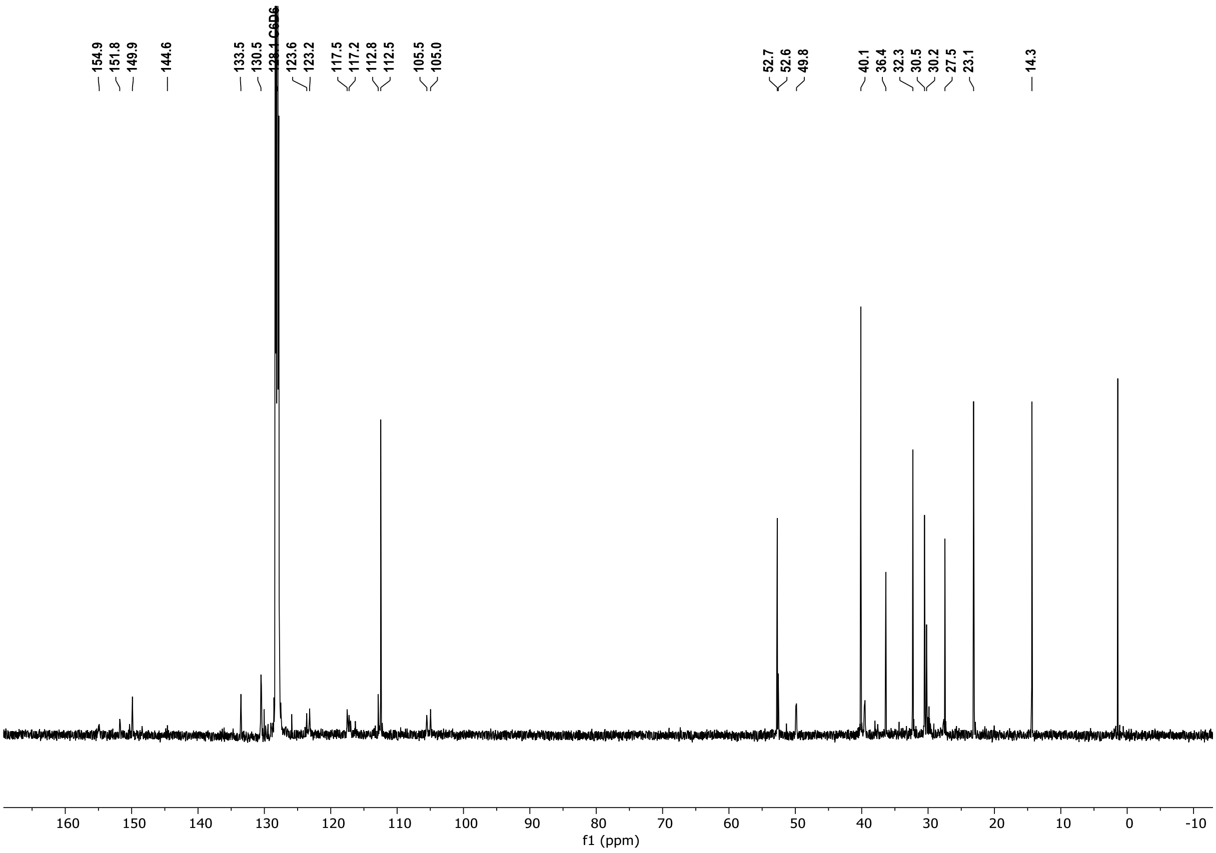

^{1}H NMR (400 MHz, C_6D_6) for **5l**

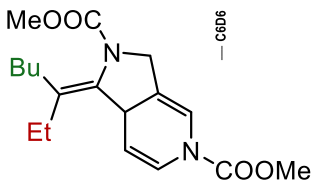

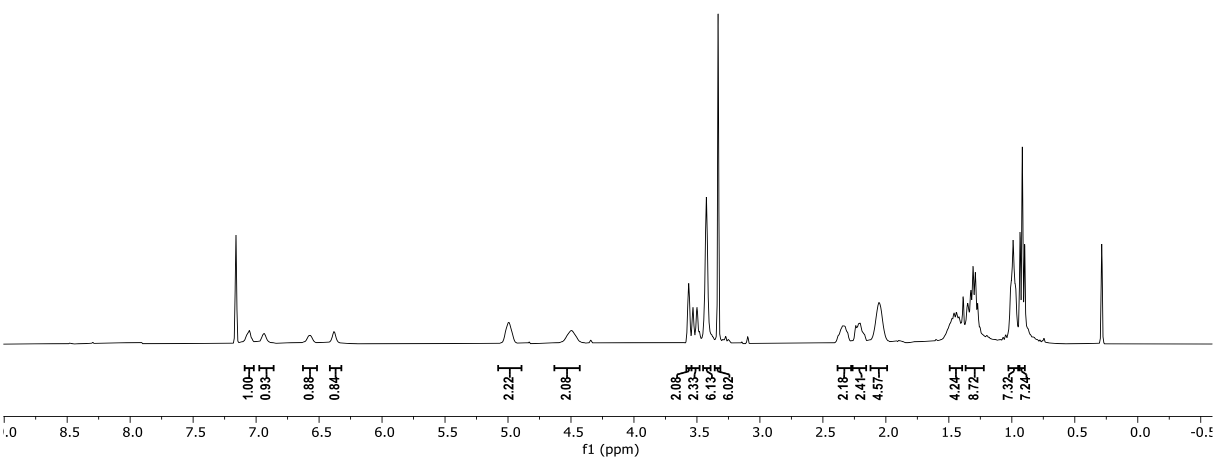

^{13}C NMR (101 MHz, C_6D_6) for **5l**

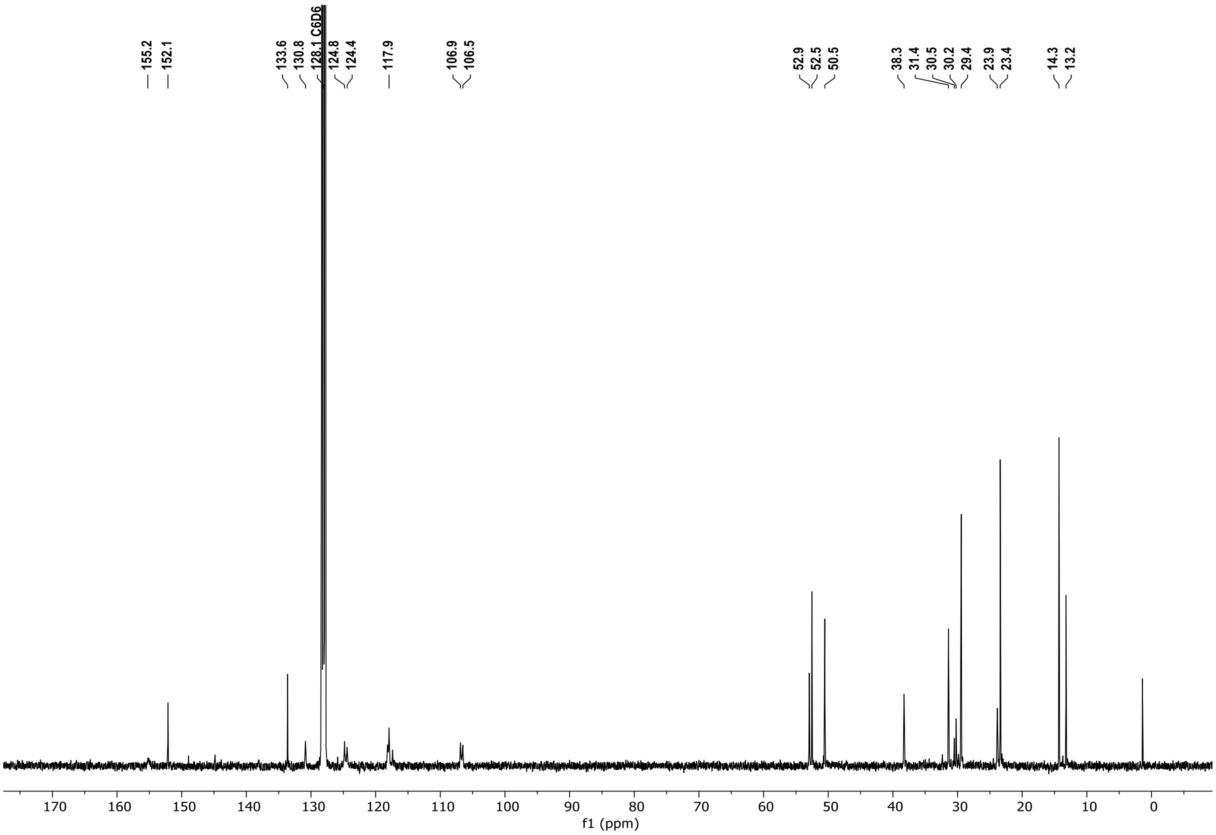

^{1}H NMR (500 MHz, C6D6) for **5m**

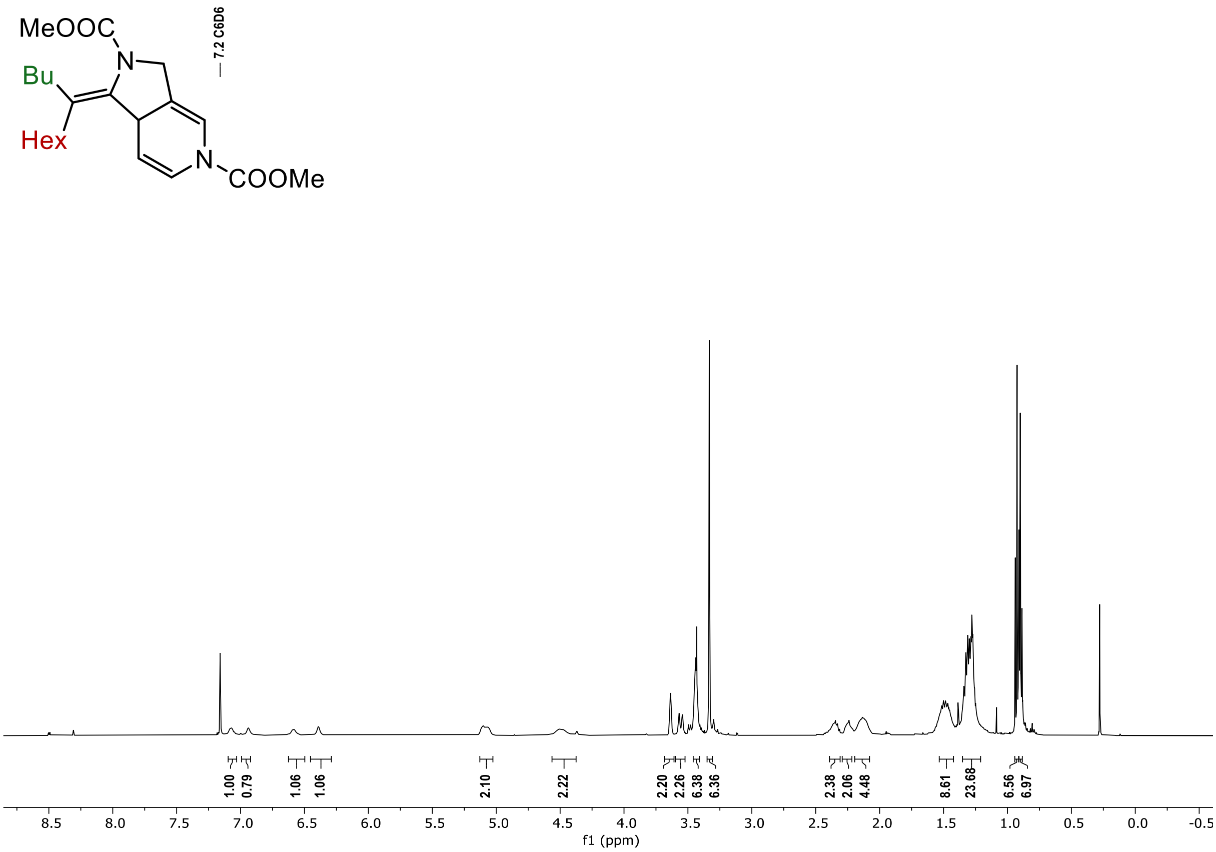

^{13}C NMR (126 MHz, C_6D_6) for **5m**

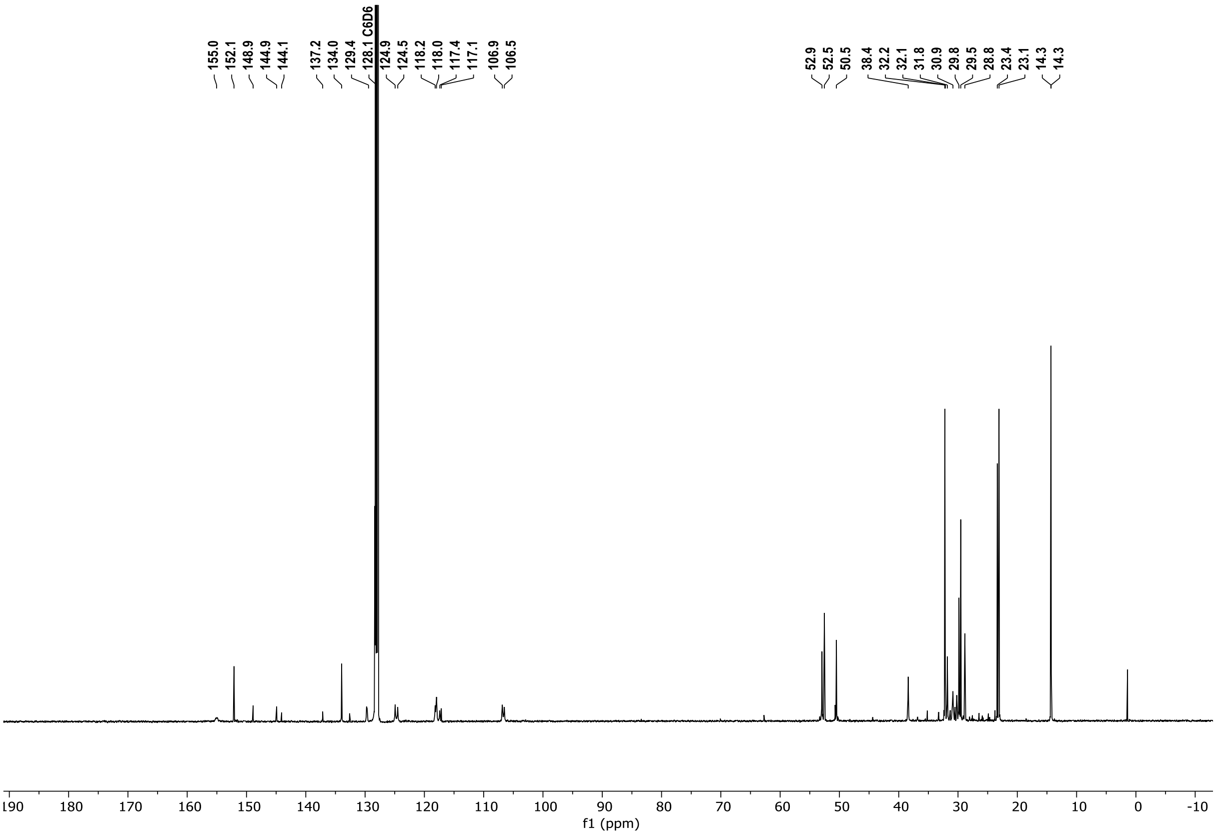

^{1}H NMR (400 MHz, C_6D_6) for **5n**

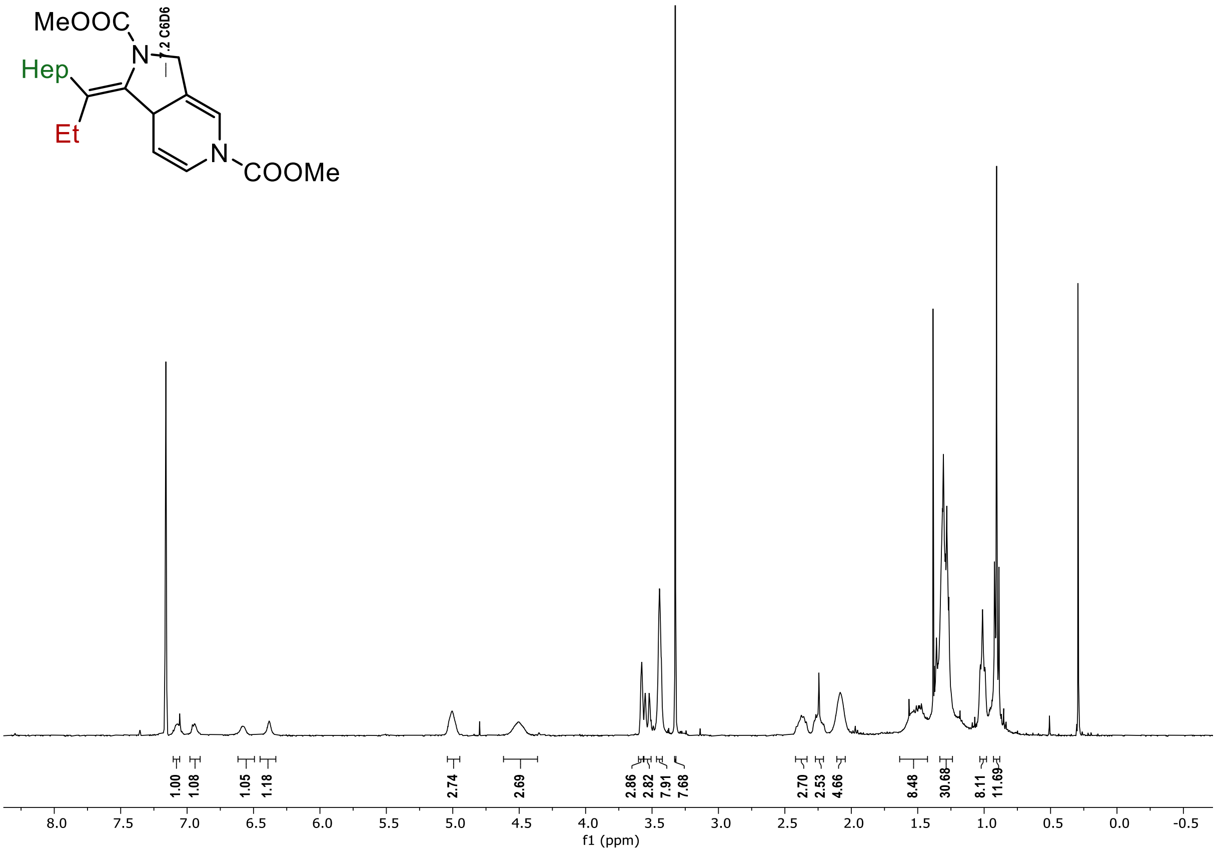

^{13}C NMR (101 MHz, C_6D_6) for **5n**

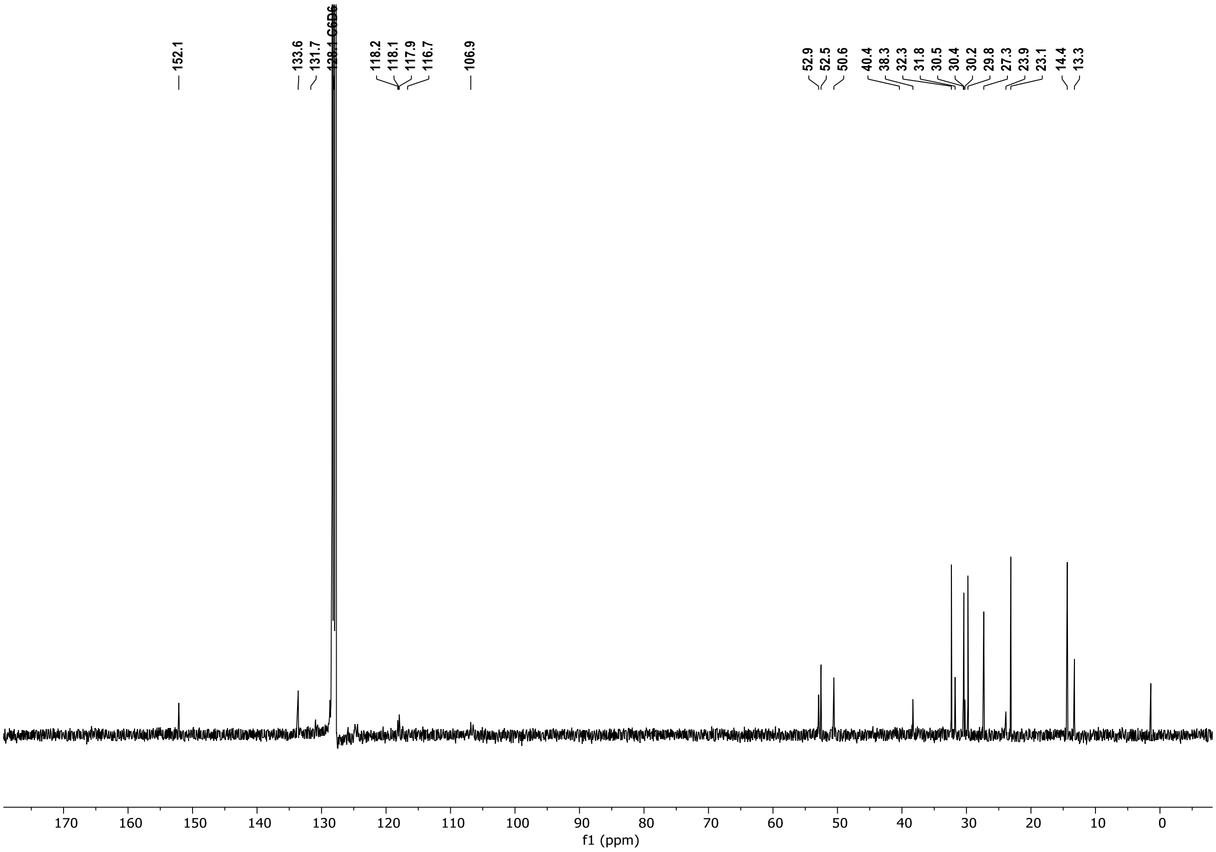

^{1}H NMR (400 MHz, C_6D_6) for **5o**

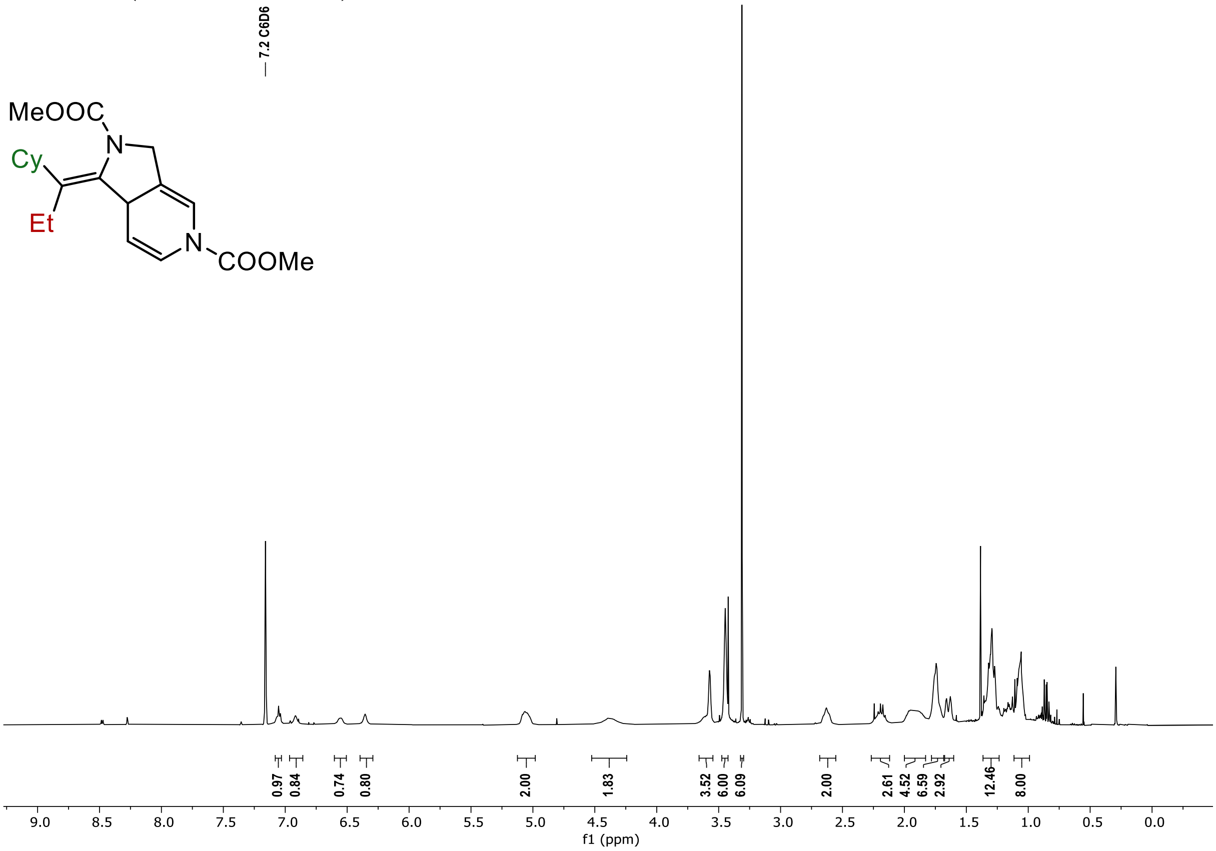

^{13}C NMR (101 MHz, C_6D_6) for **5o**

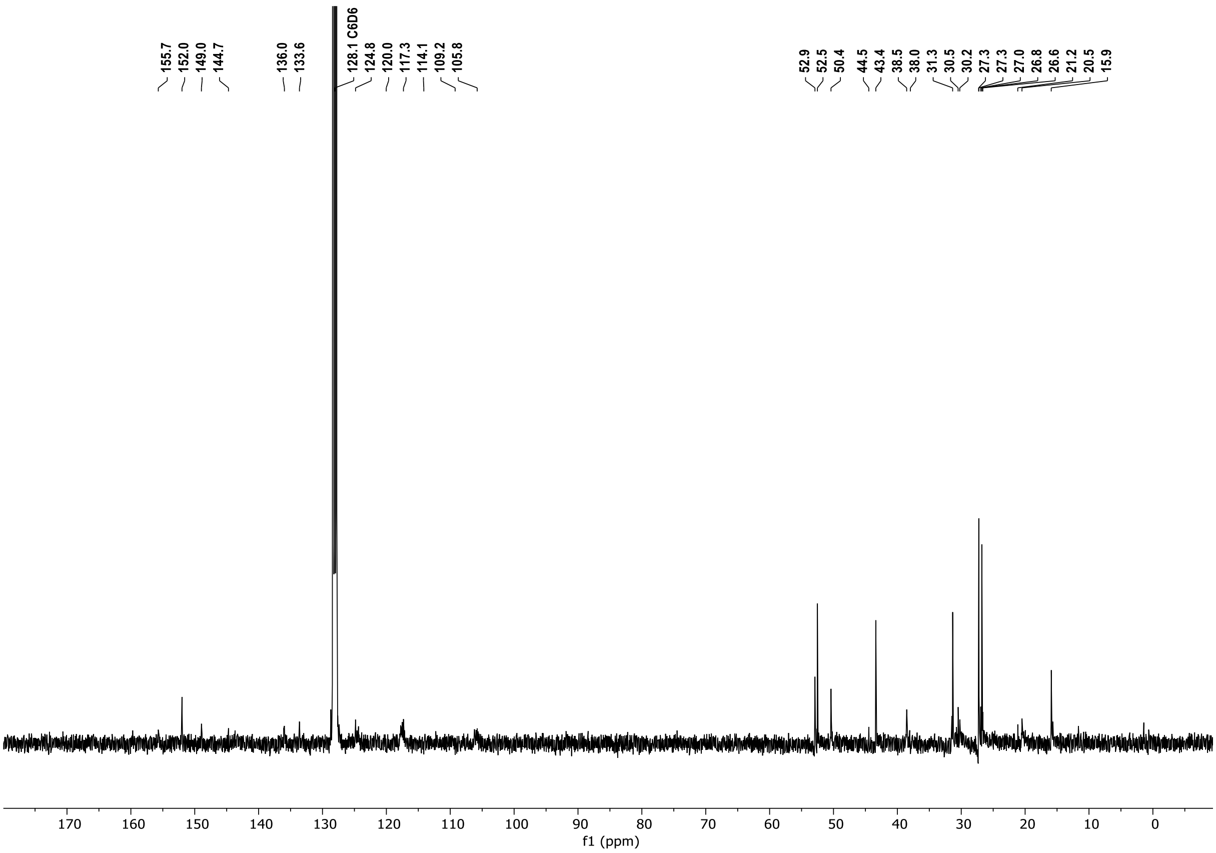

^{1}H NMR (400 MHz, C_6D_6) for **5p**

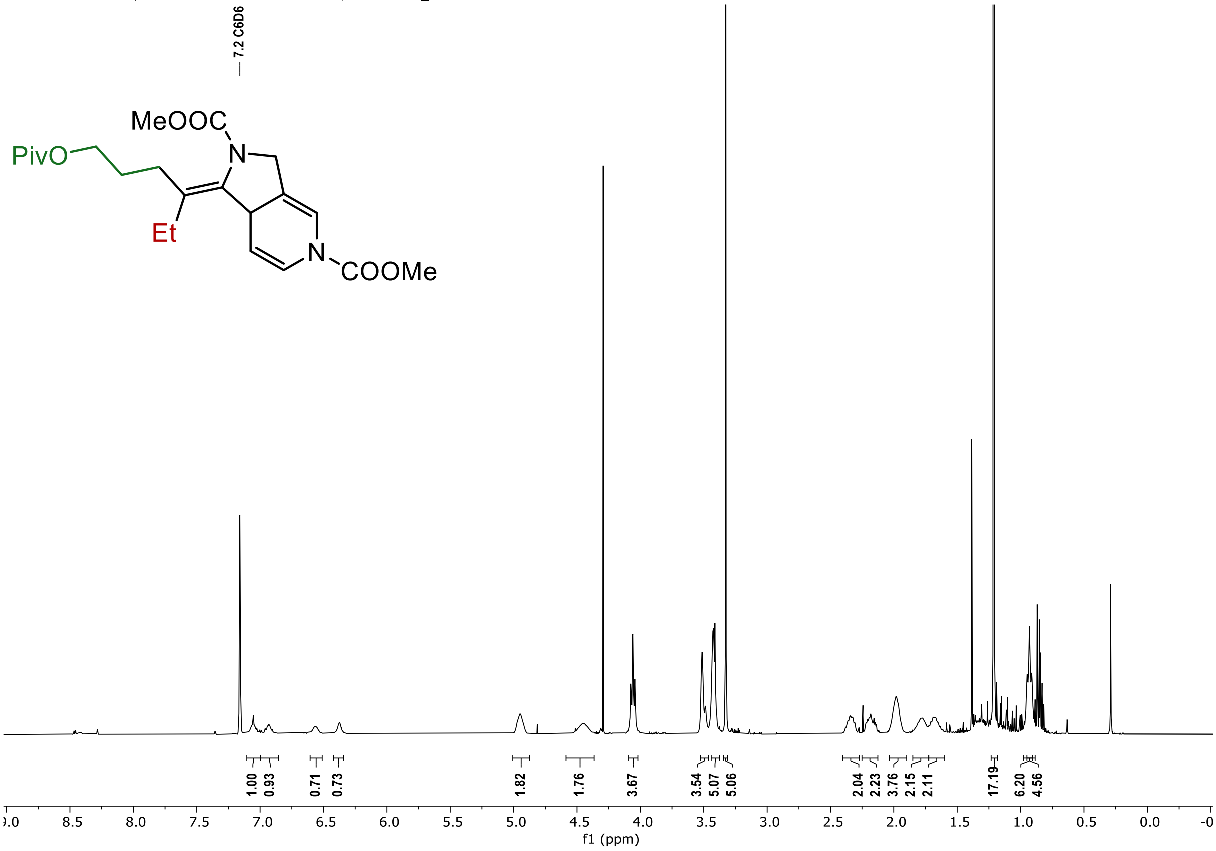

^{13}C NMR (101 MHz, C_6D_6) for **5p**

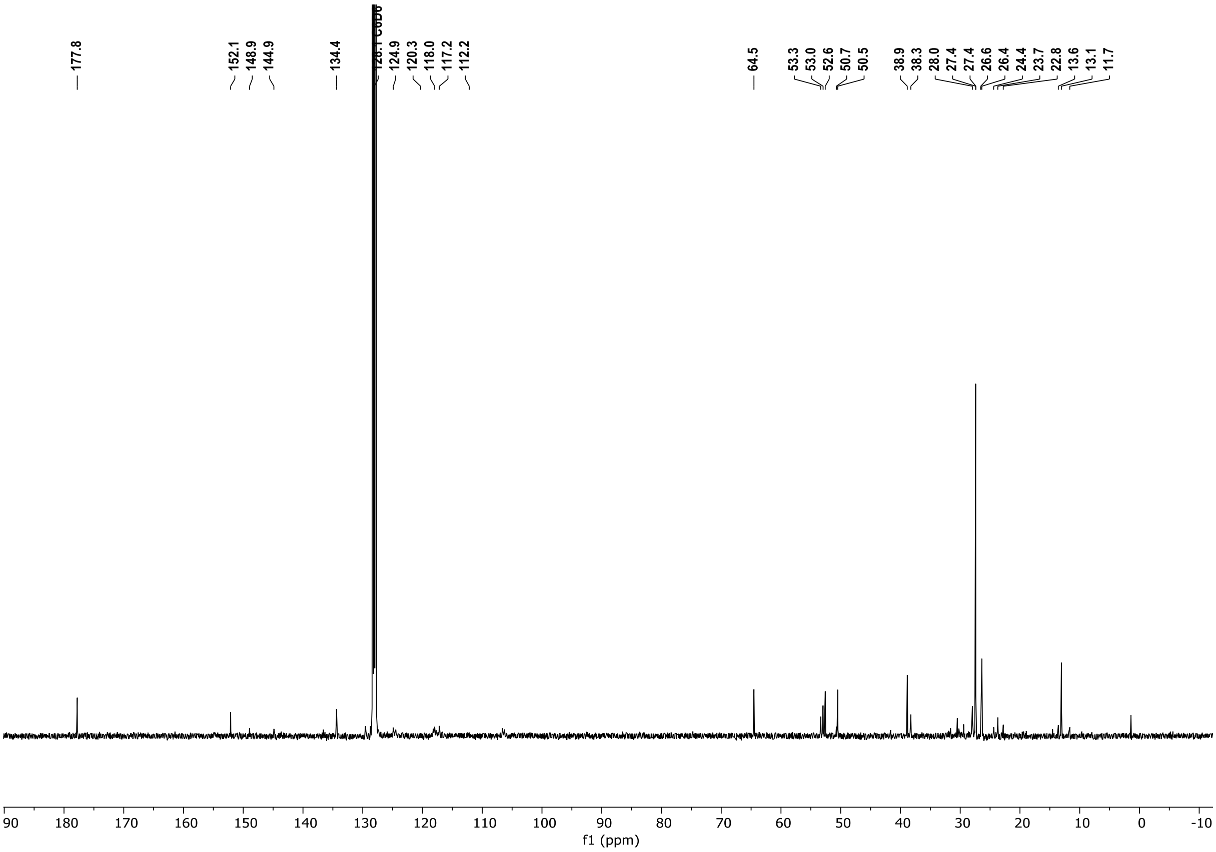

^{1}H NMR (500 MHz, C_6D_6) for **5q**

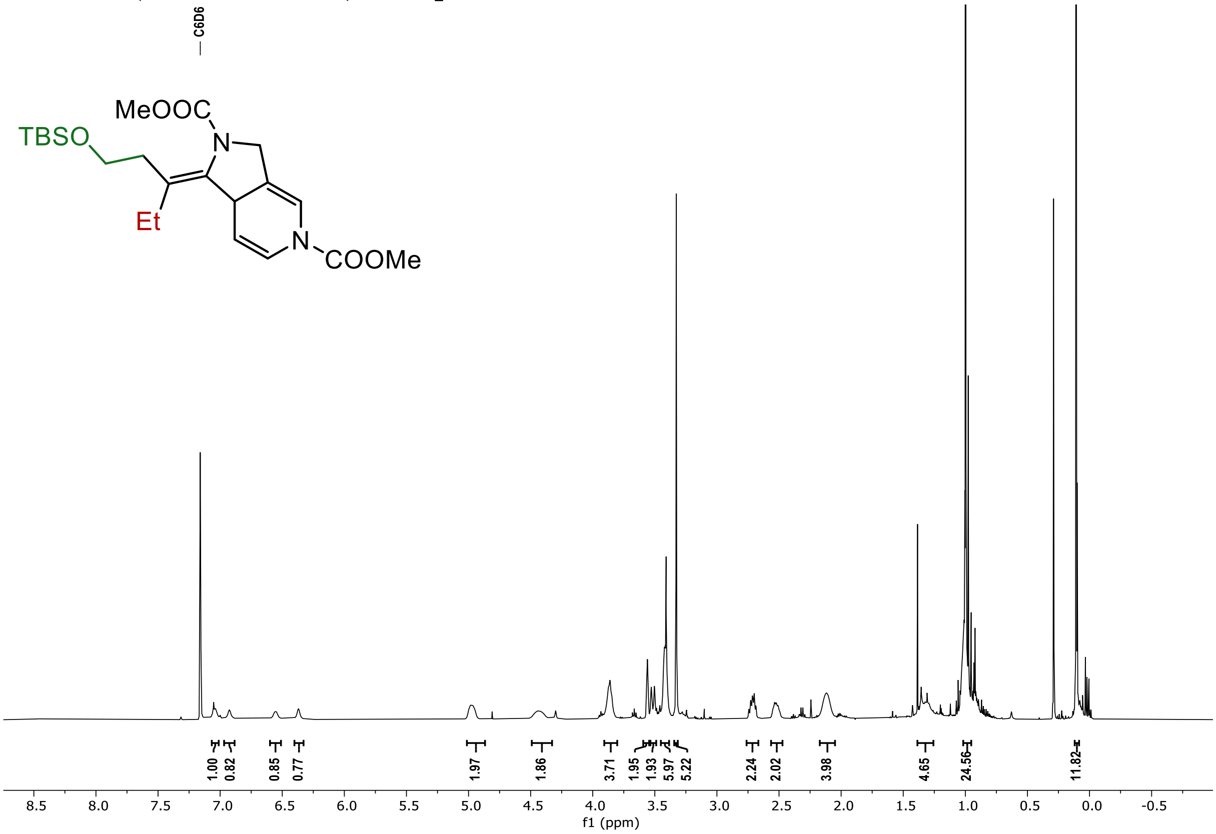

^{13}C NMR (126 MHz, C_6D_6) for **5q**

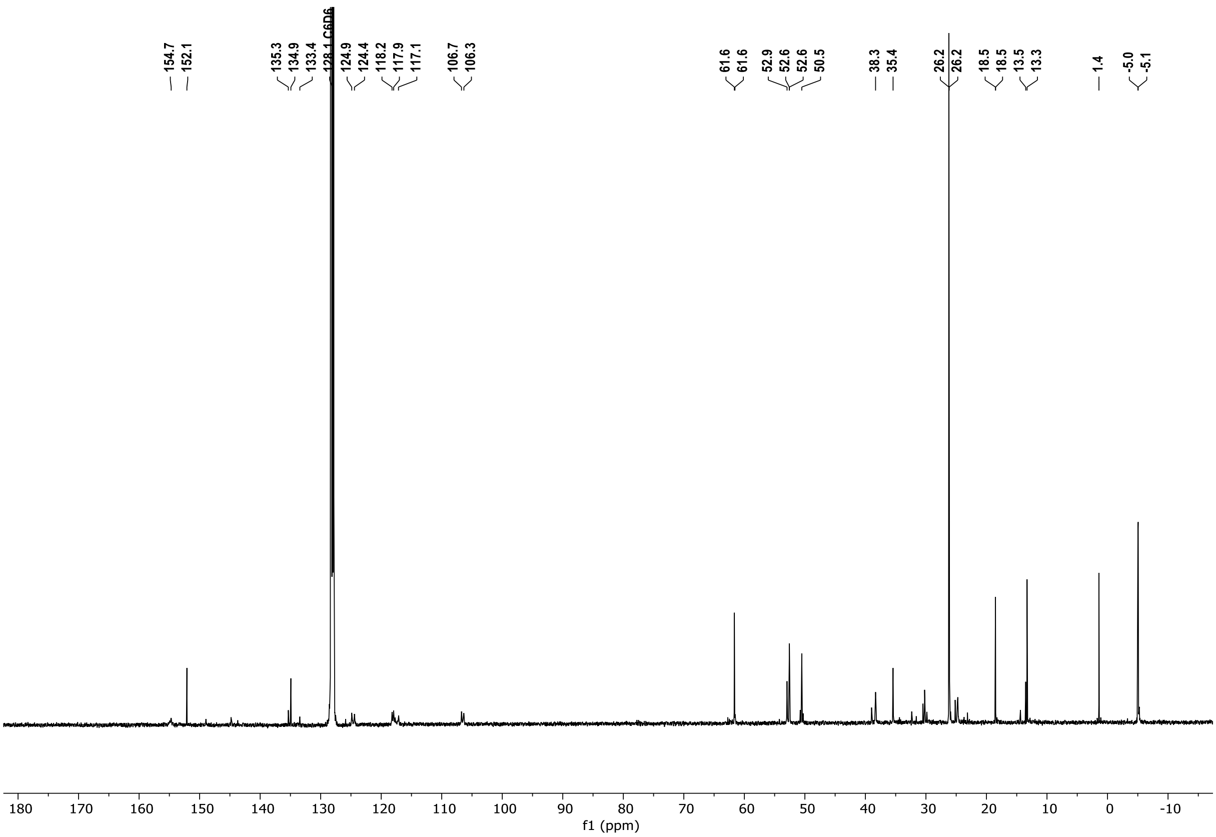

^1H NMR (400 MHz, C_6D_6) for **5r**

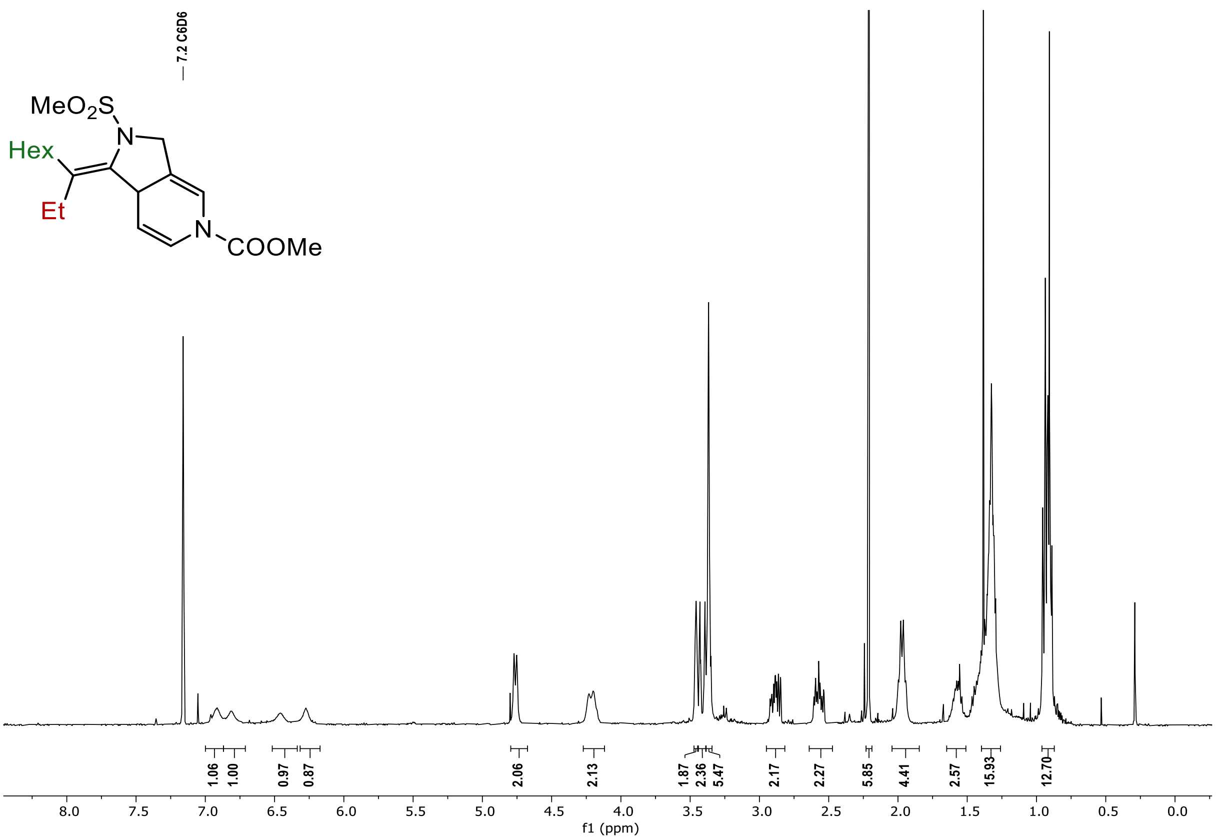

^{13}C NMR (101 MHz, C_6D_6) for **5r**

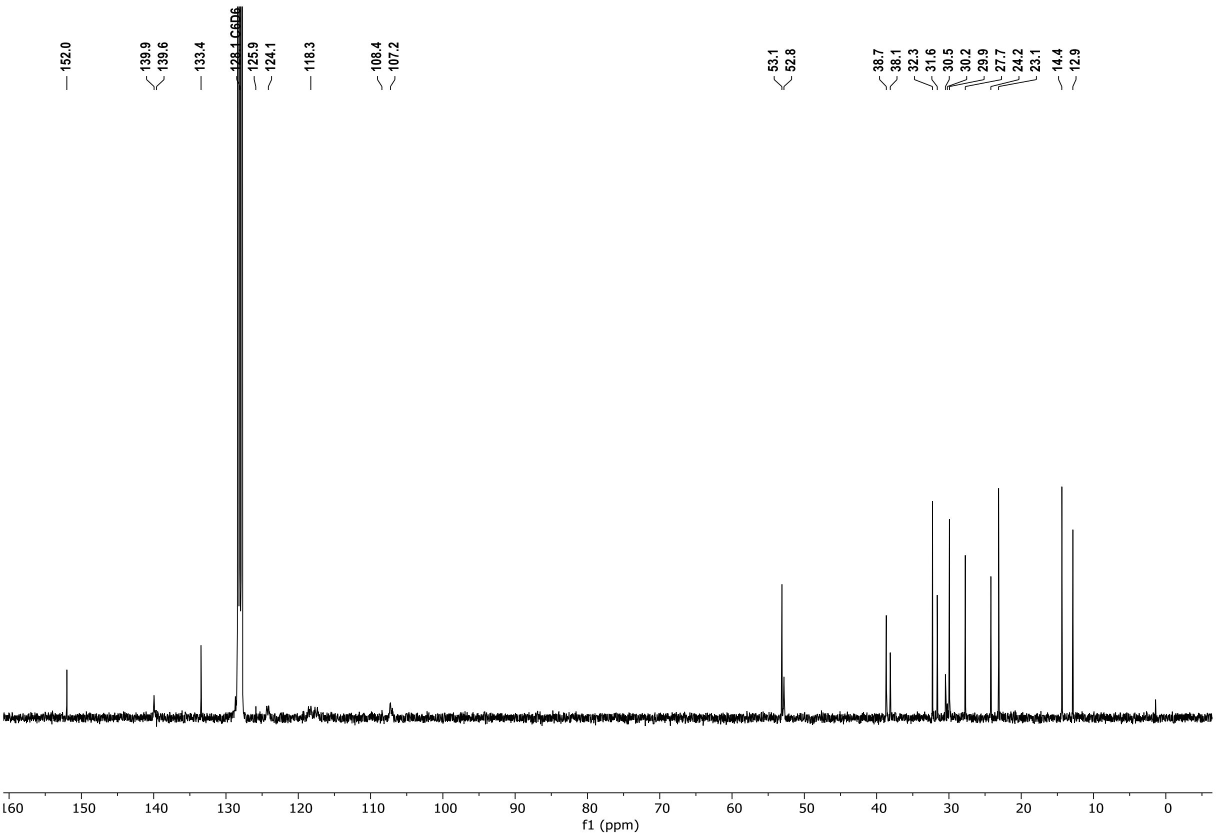

^{1}H NMR (500 MHz, C_6D_6) for **5s**

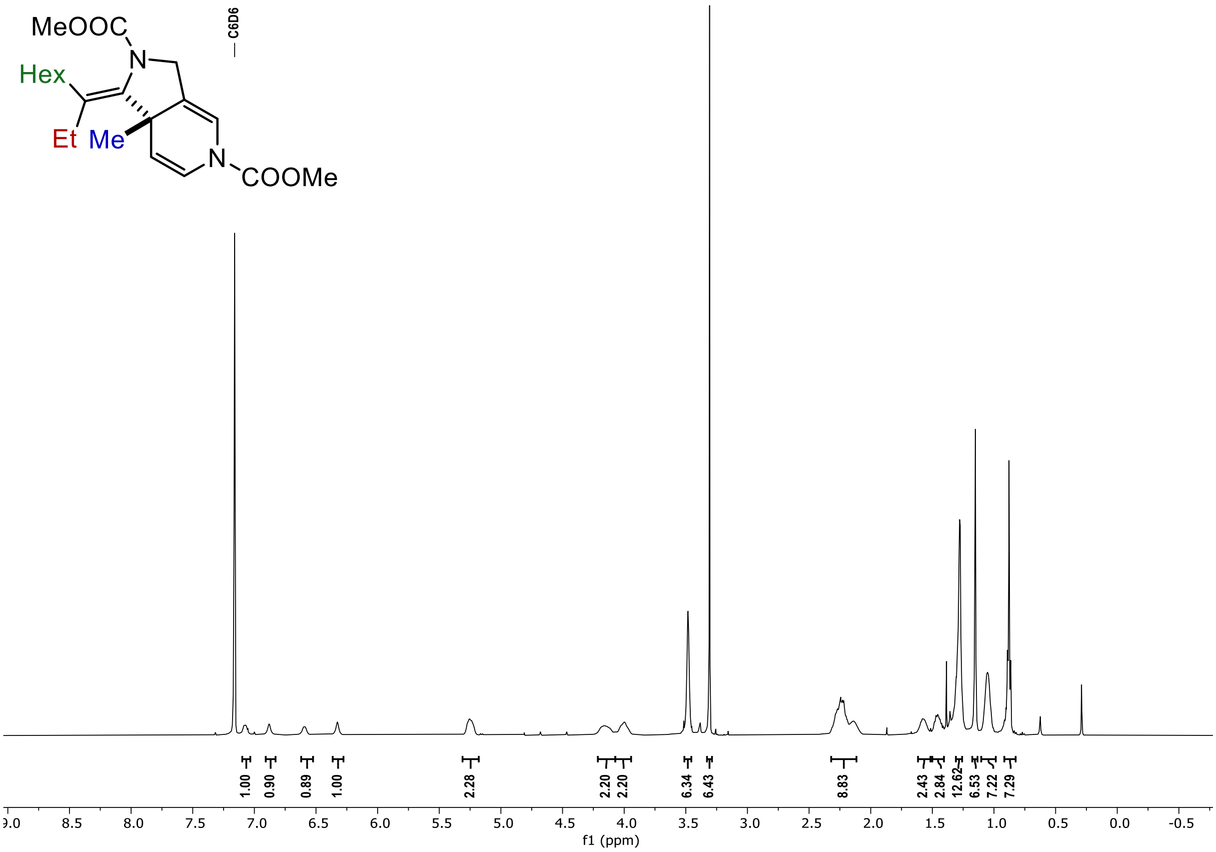

^{13}C NMR (126 MHz, C_6D_6) for **5s**

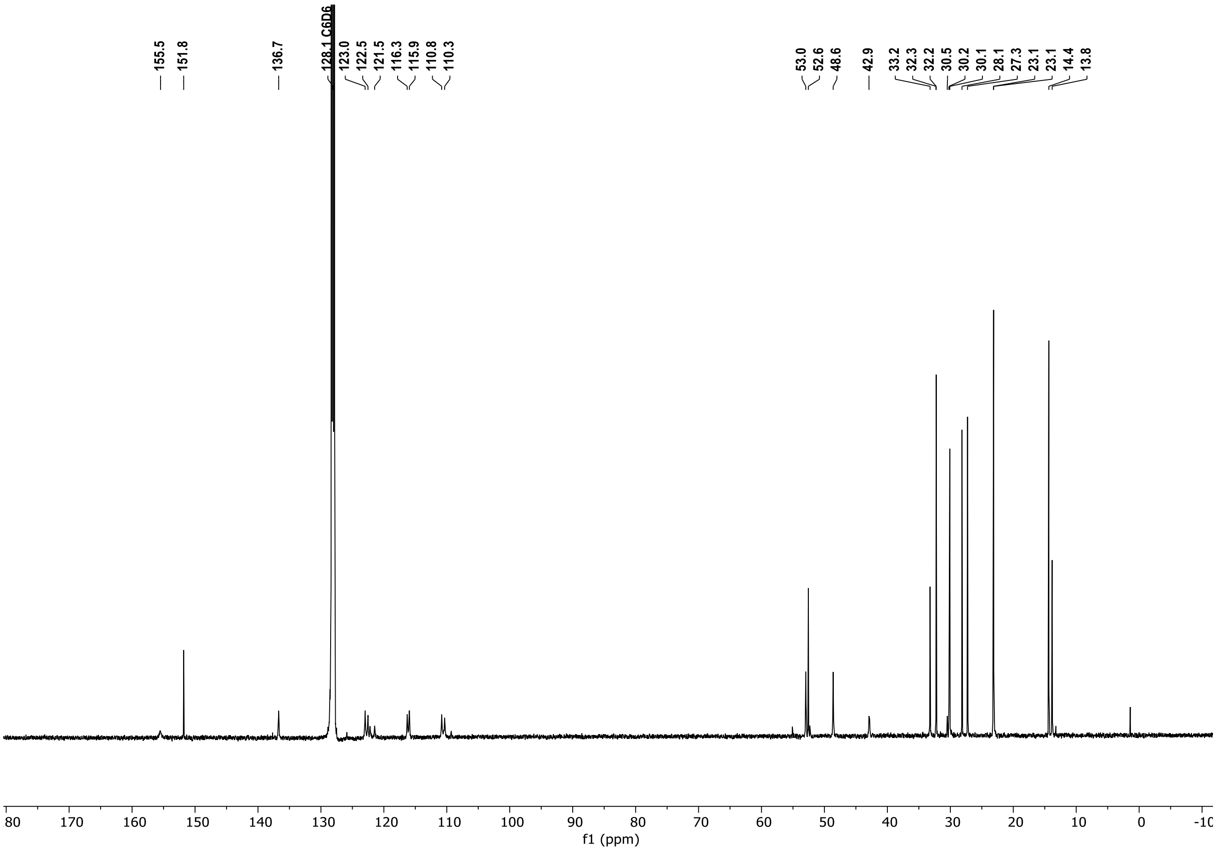

^{1}H NMR (400 MHz, C_6D_6) for **5t**

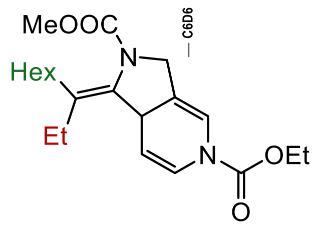

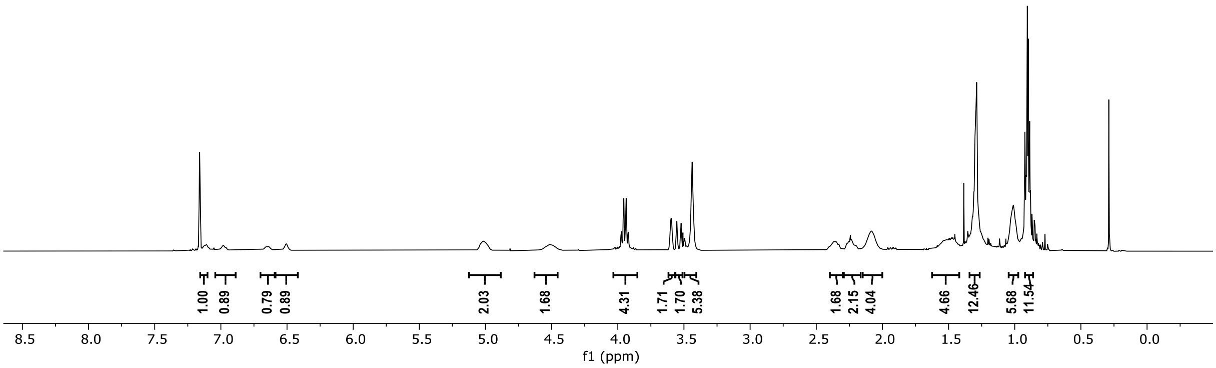

^{13}C NMR (101 MHz, C_6D_6) for **5t**

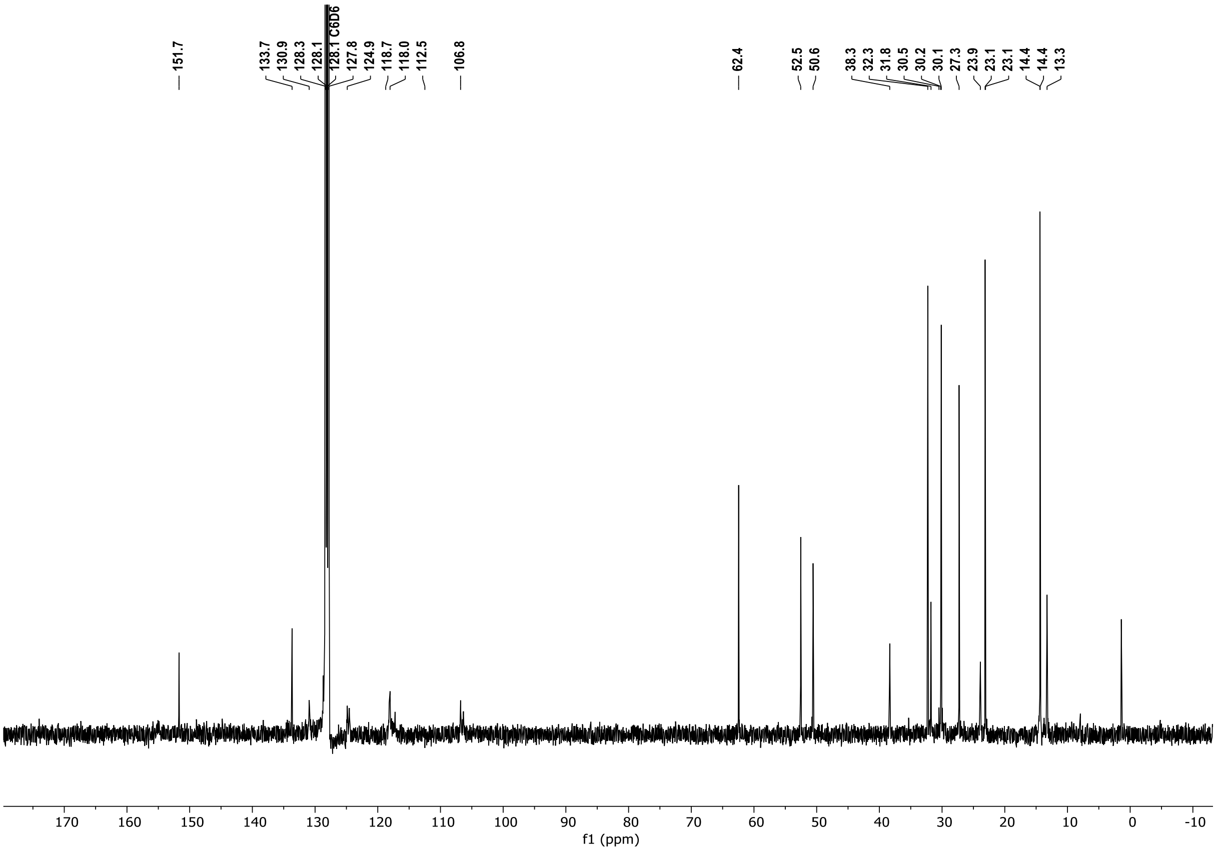

^{1}H NMR (400 MHz, C_6D_6) for **5u**

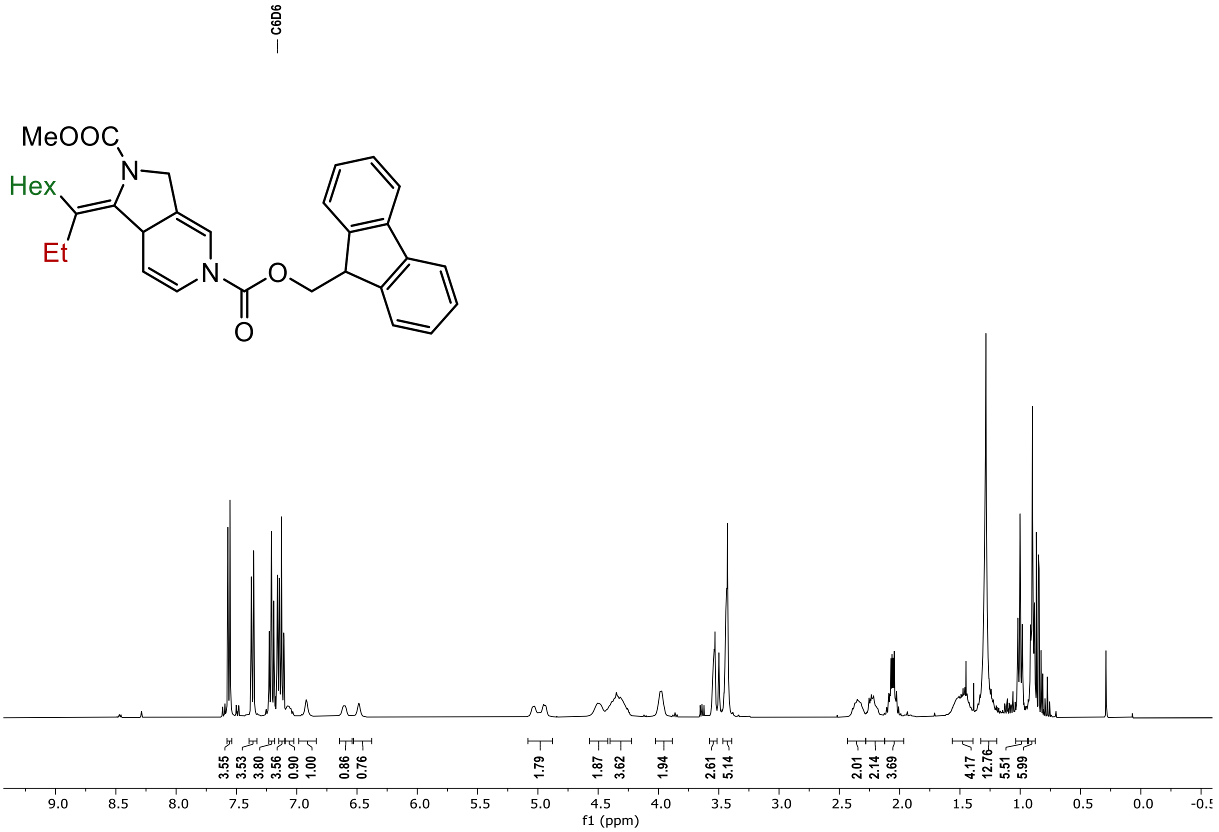

^{13}C NMR (101 MHz, C_6D_6) for **5u**

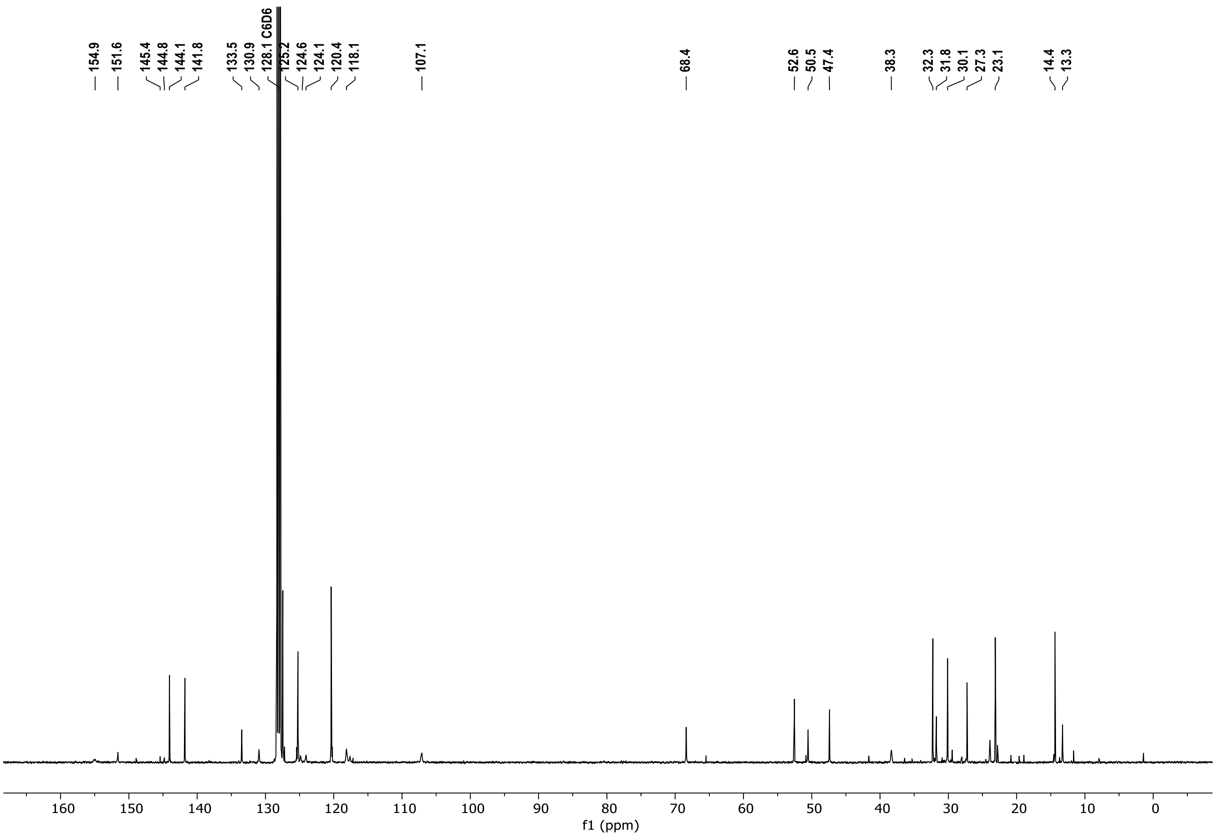

^{1}H NMR (400 MHz, C_6D_6) for **5v**

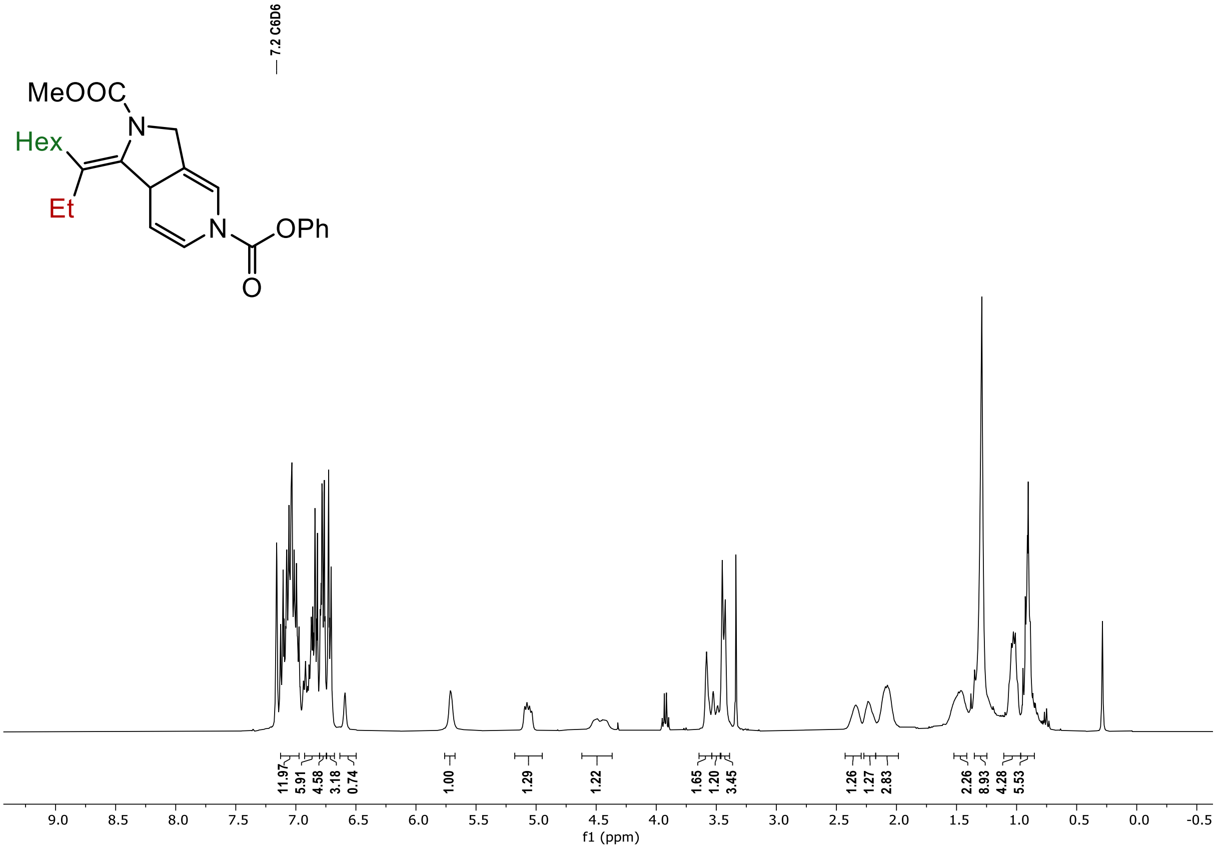

^{13}C NMR (101 MHz, C_6D_6) **5v**

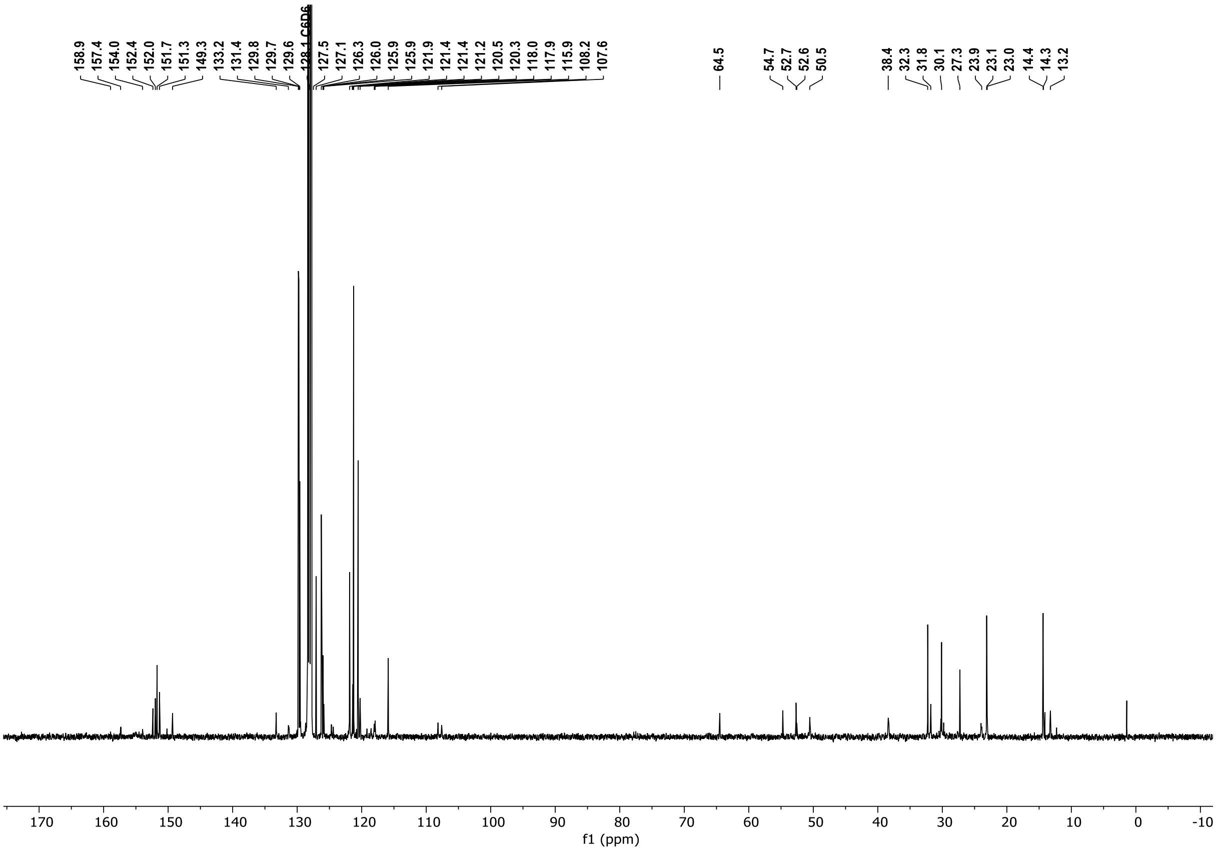

^{1}H NMR (400 MHz, C_6D_6) for **7a**

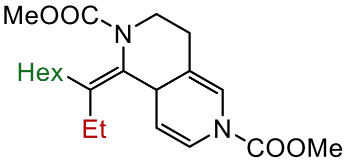

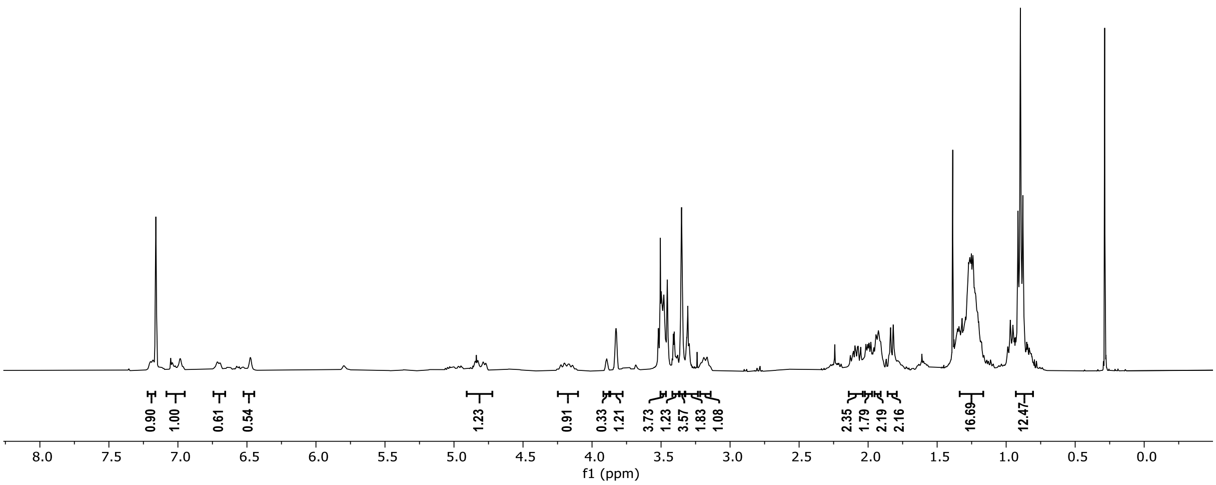

^{13}C NMR (101 MHz, C_6D_6) for **7a**

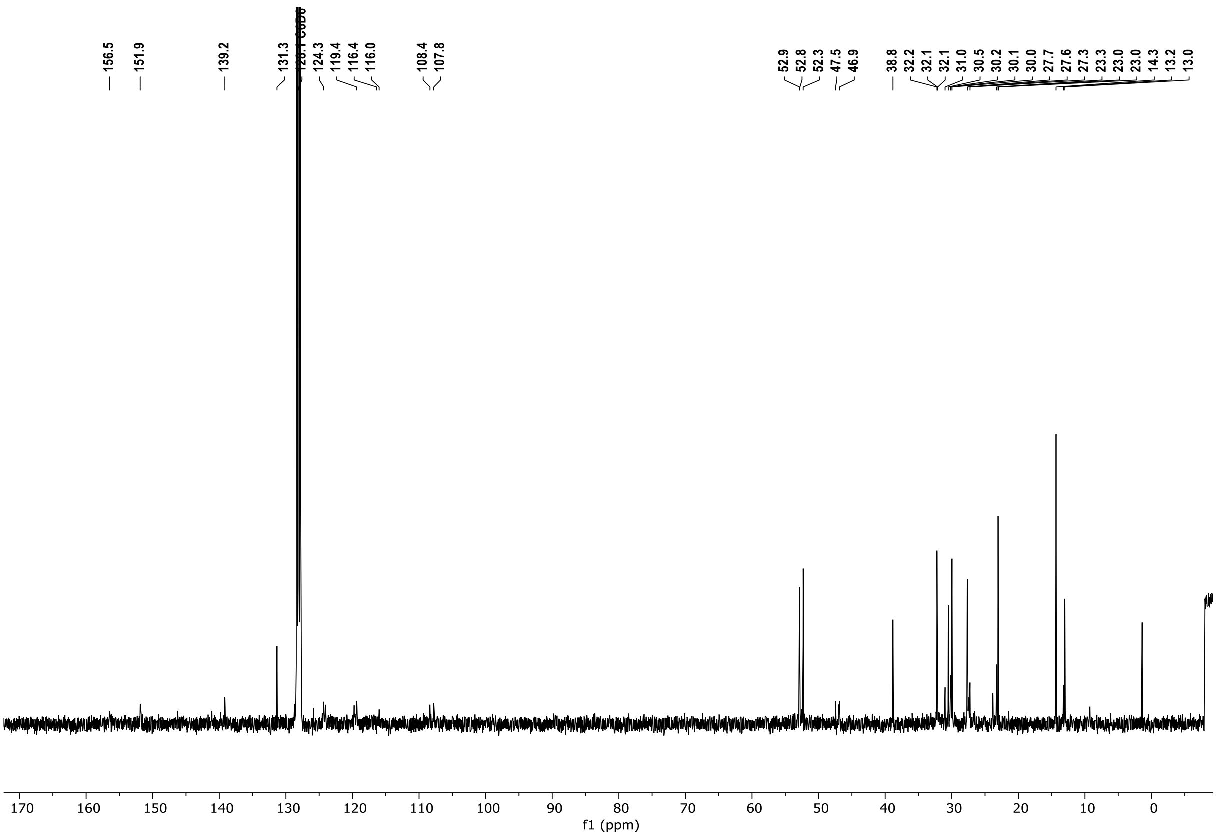

^{1}H NMR (400 MHz, C_6D_6) for **7b**

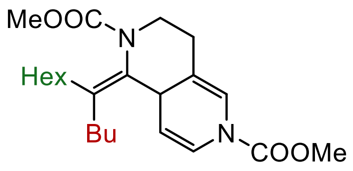

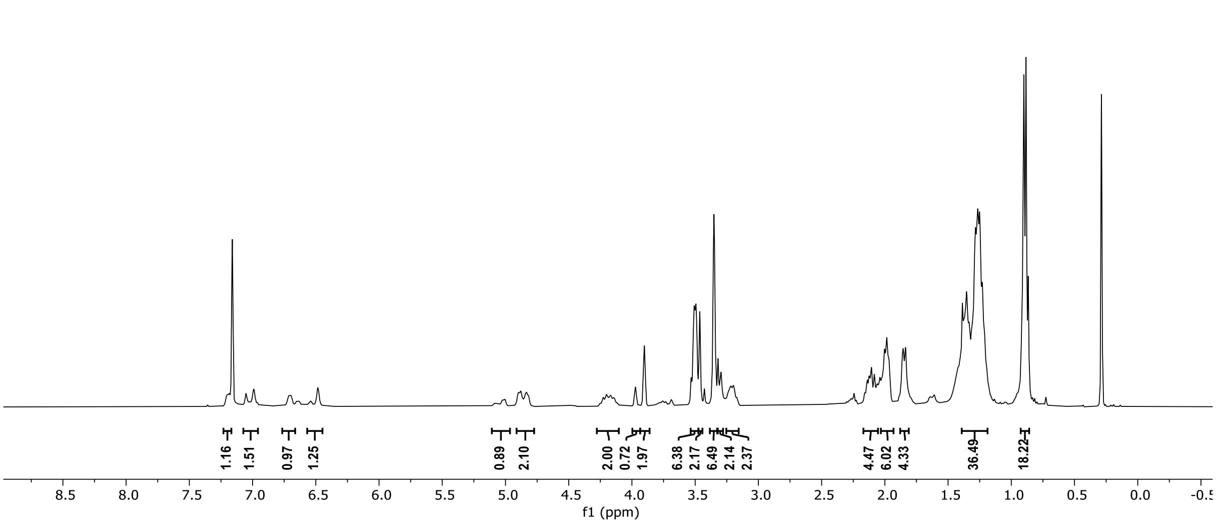

^{13}C NMR (101 MHz, C_6D_6) for **7b**

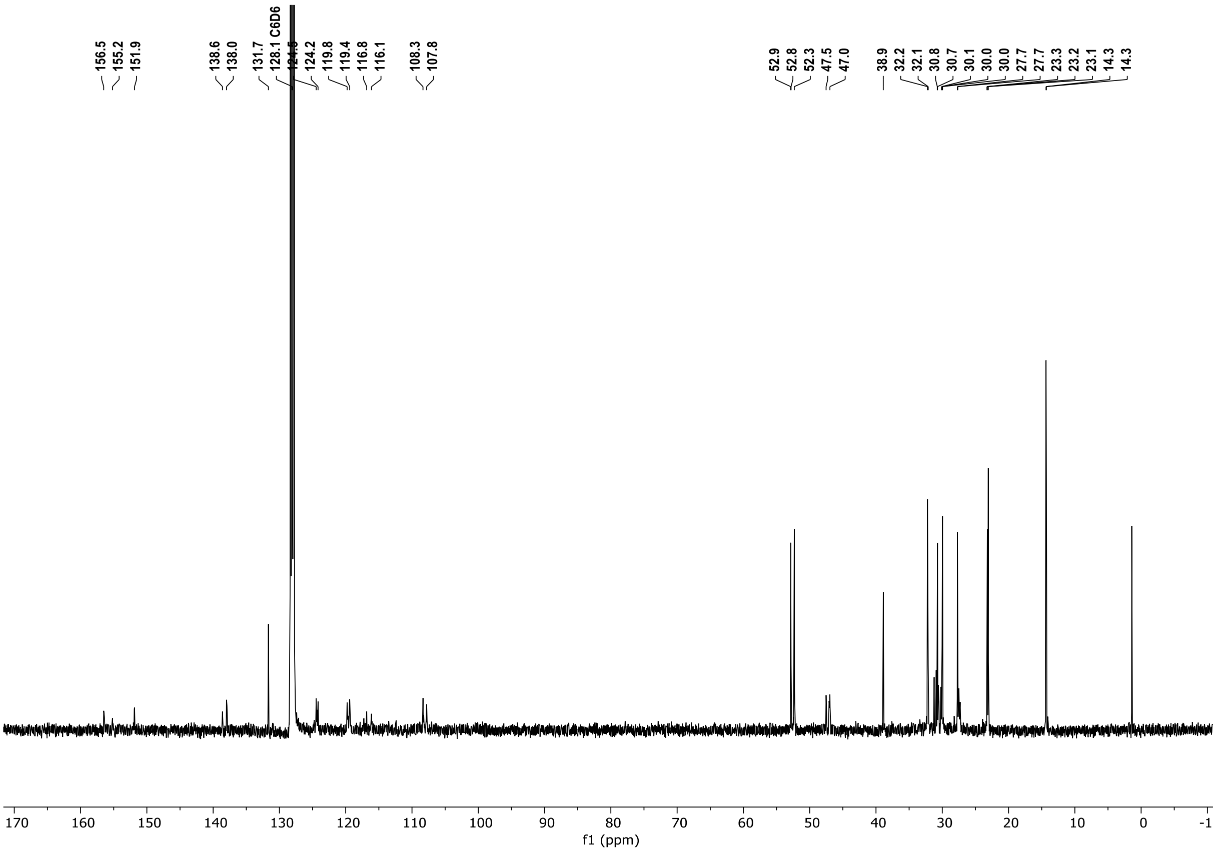

^1H NMR (500 MHz, C_6D_6) for **7c**

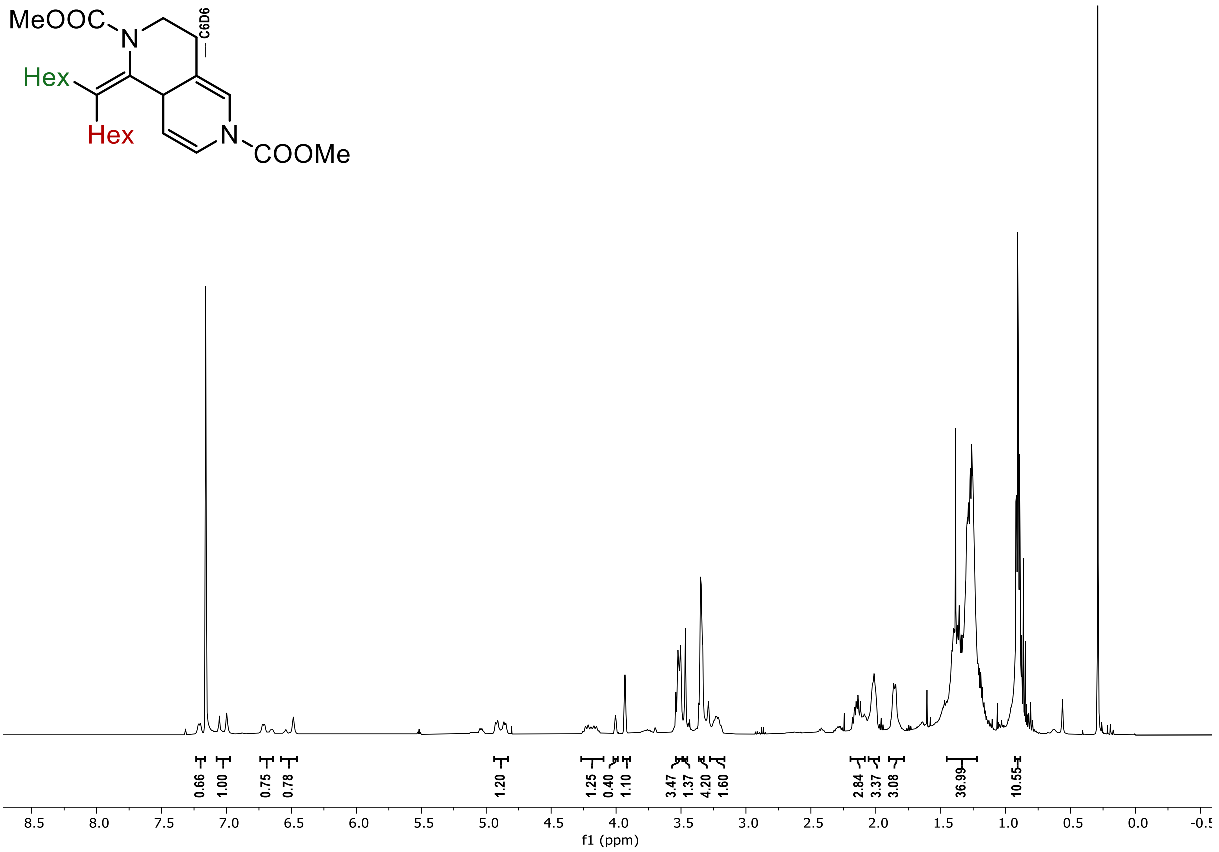

^{13}C NMR (126 MHz, C_6D_6) for **7c**

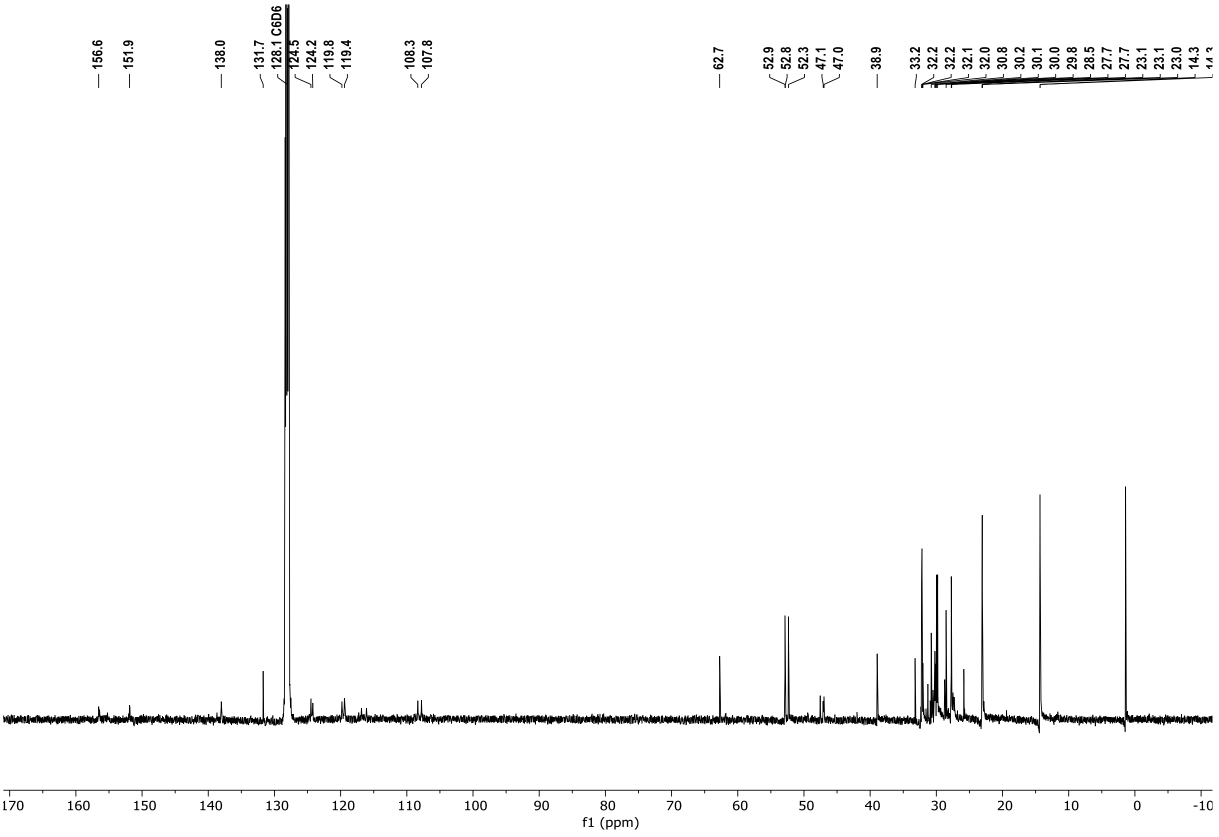

^{1}H NMR (500 MHz, C_6D_6) for **7d**

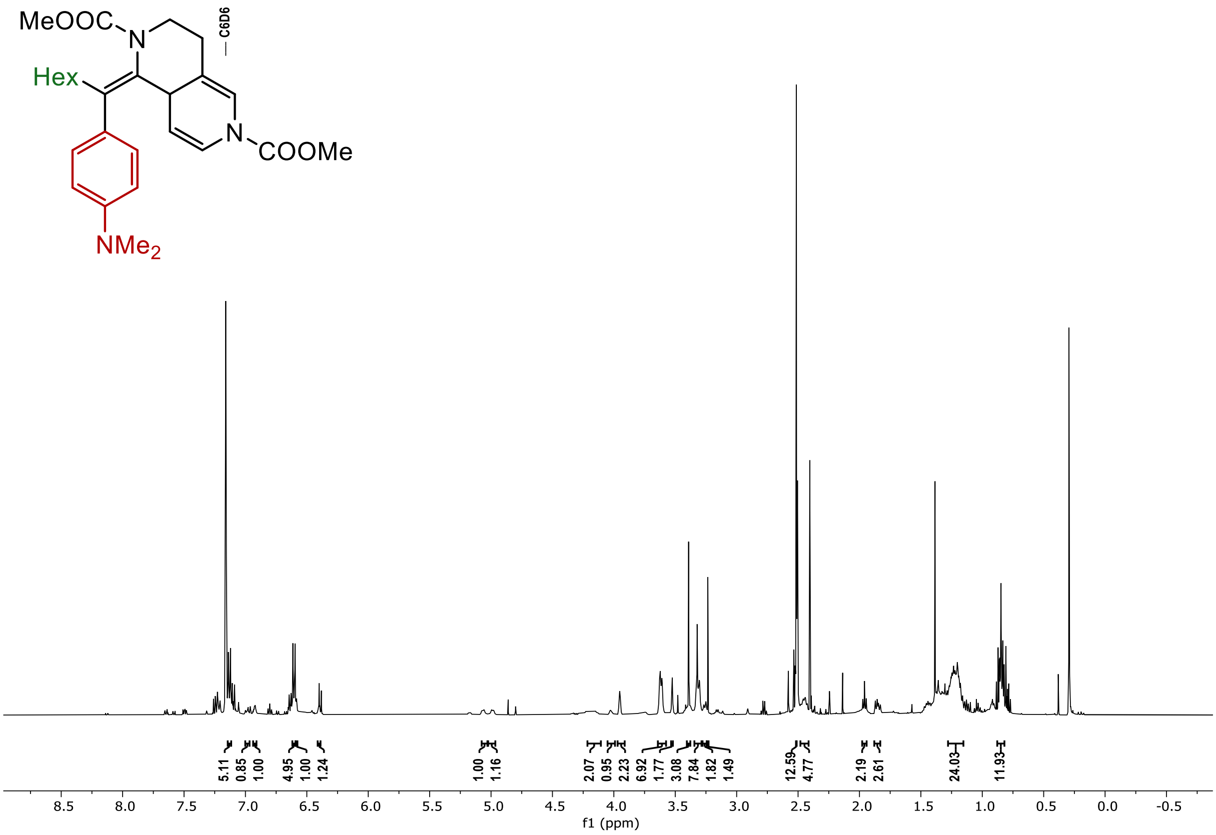

^{13}C NMR (126 MHz, C_6D_6) for **7d**

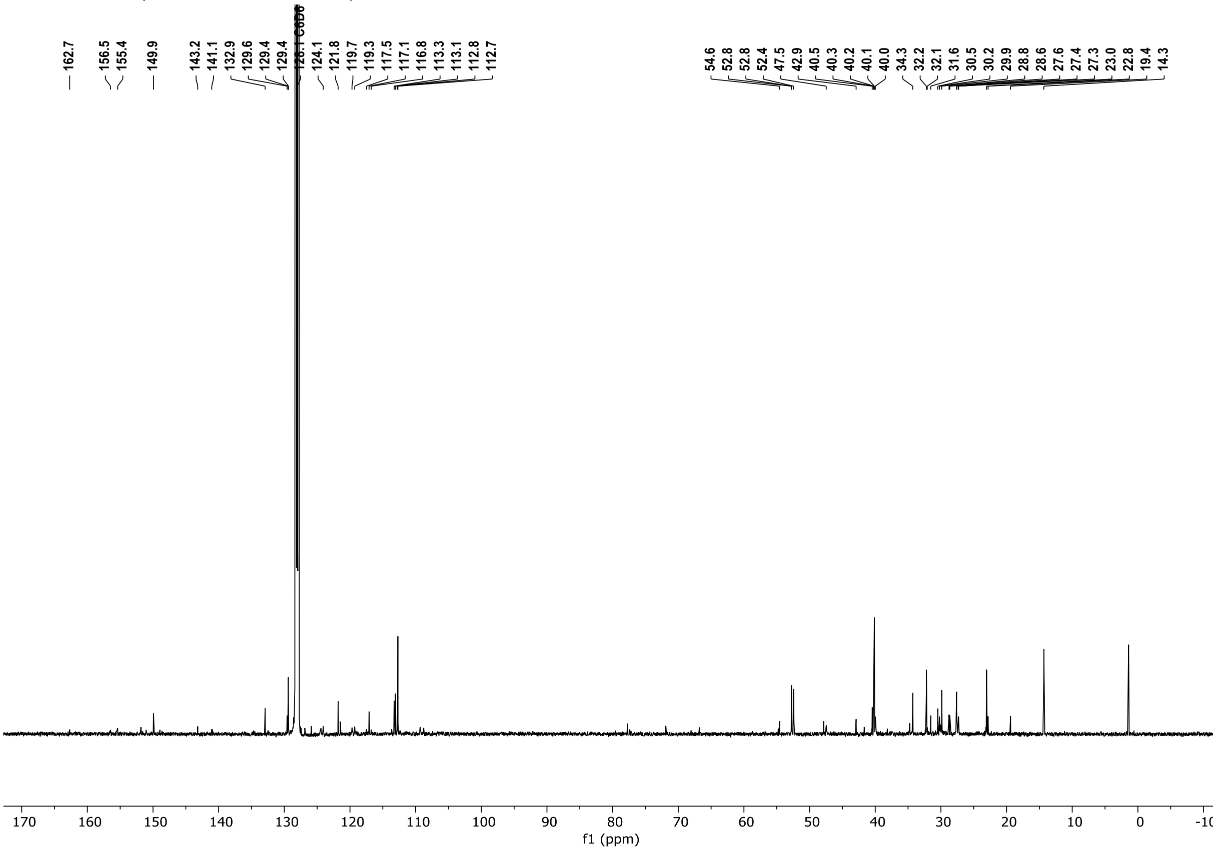

^{1}H NMR (400 MHz, C_6D_6) for **7e**

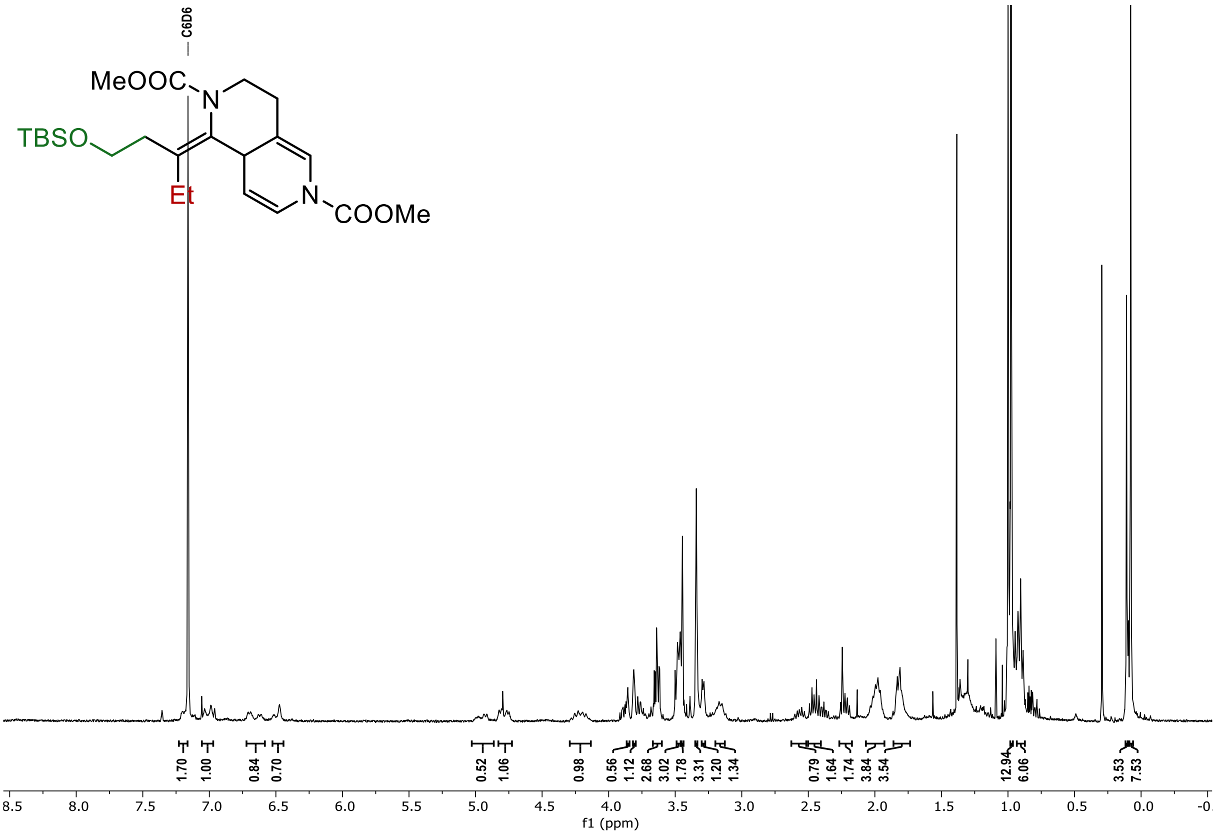

^{13}C NMR (126 MHz, C_6D_6) for **7e**

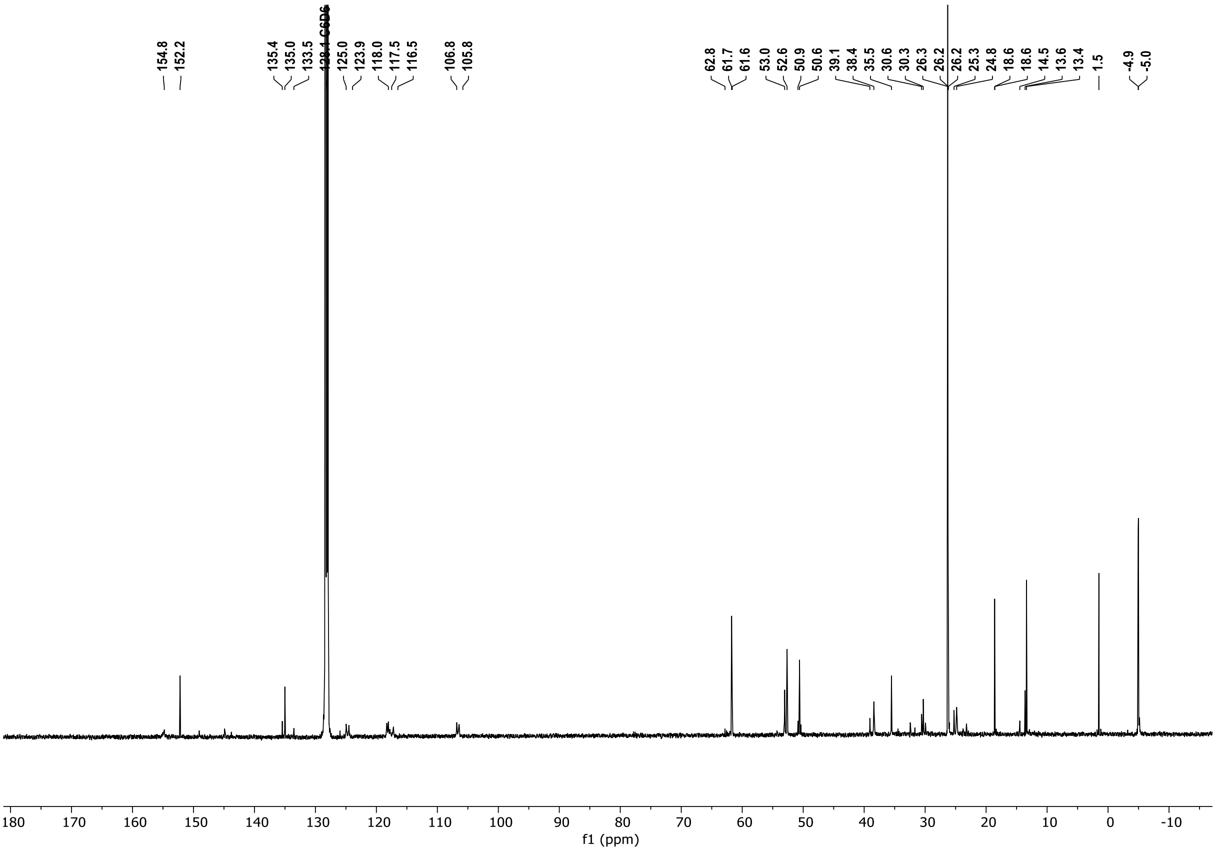

^{1}H NMR (500 MHz, $CDCl_3$) for **8a**

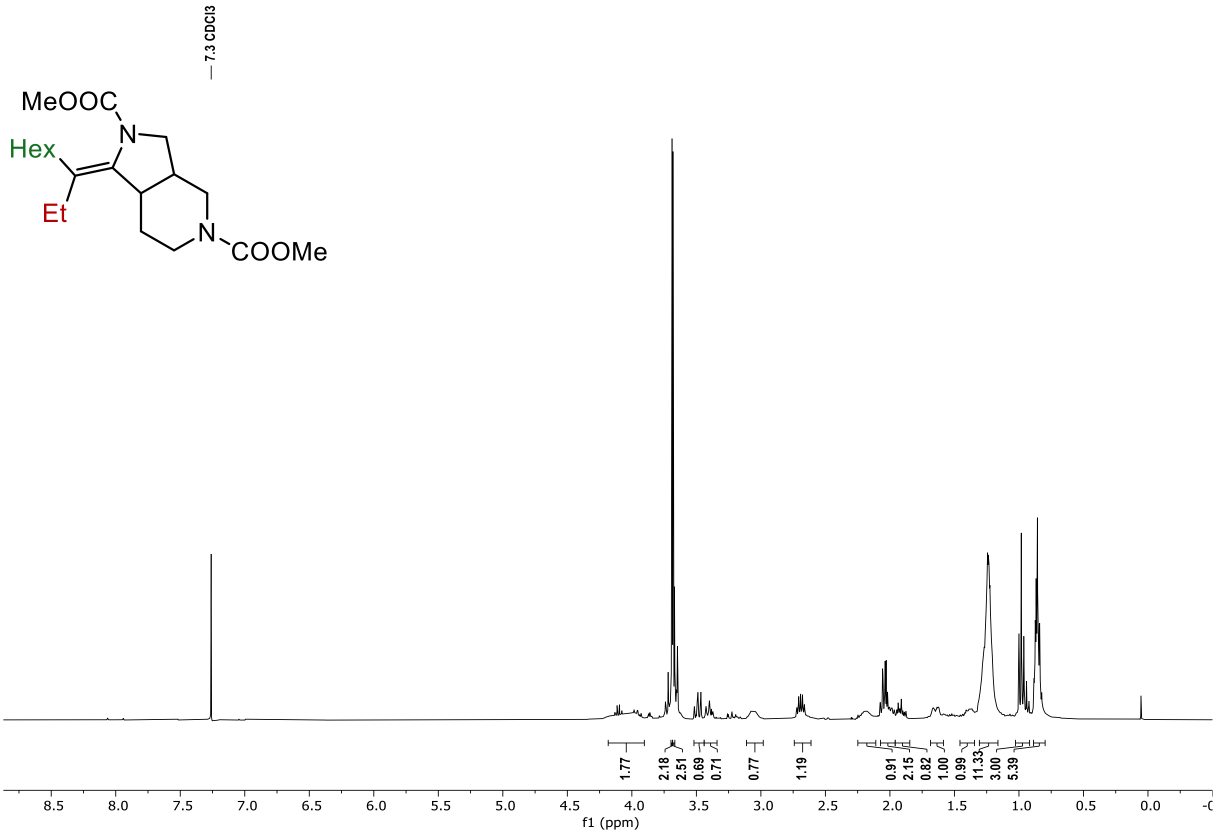

^{13}C NMR (126 MHz, $CDCl_3$) for **8a**

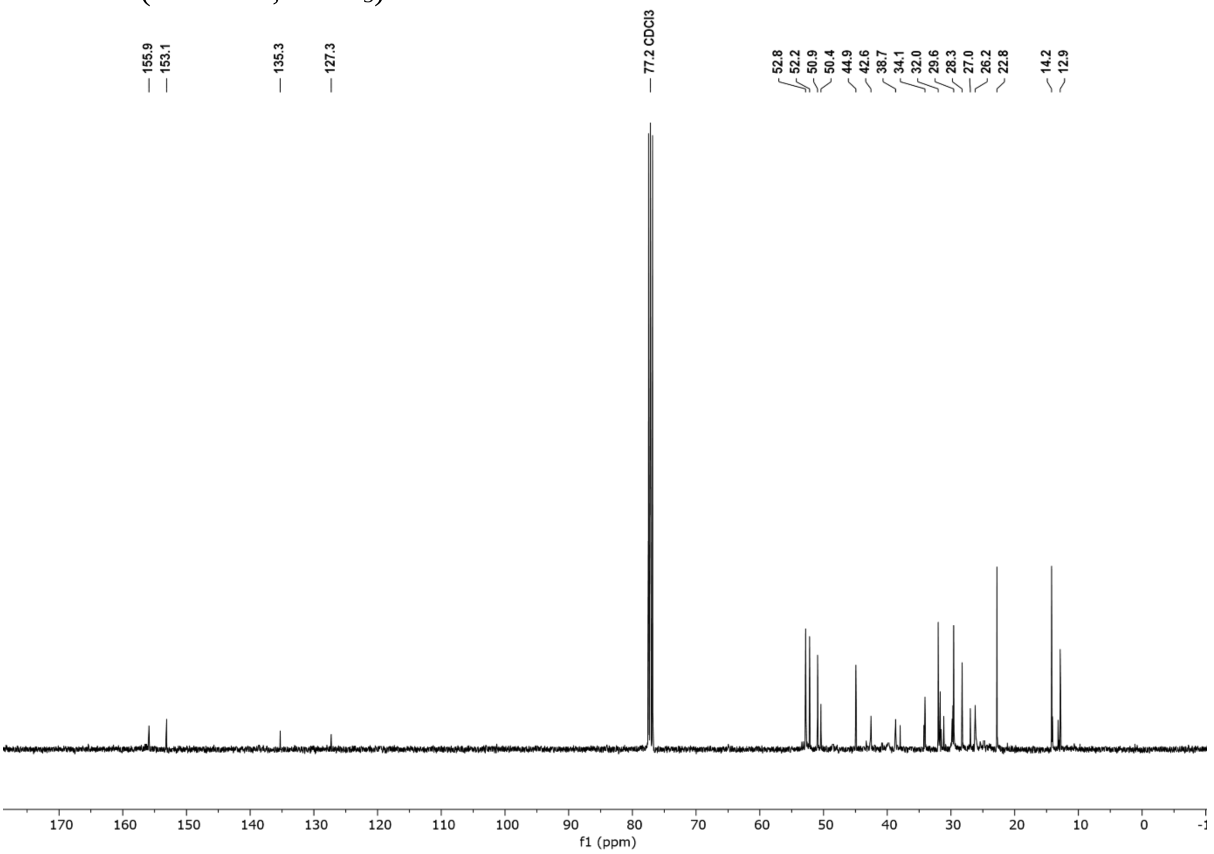

^{1}H NMR (500 MHz, $CDCl_3$) for **9a**

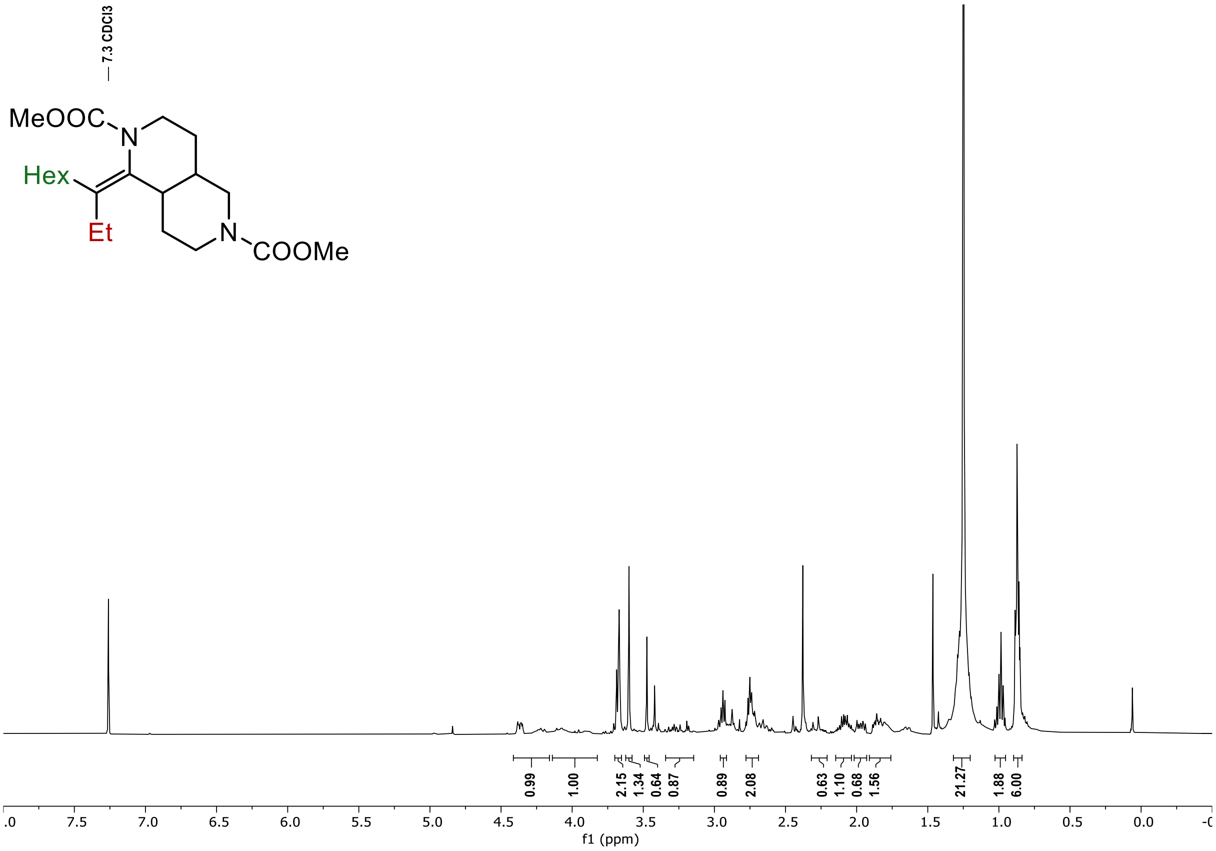

^{13}C NMR (126 MHz, $CDCl_3$) for **9a**

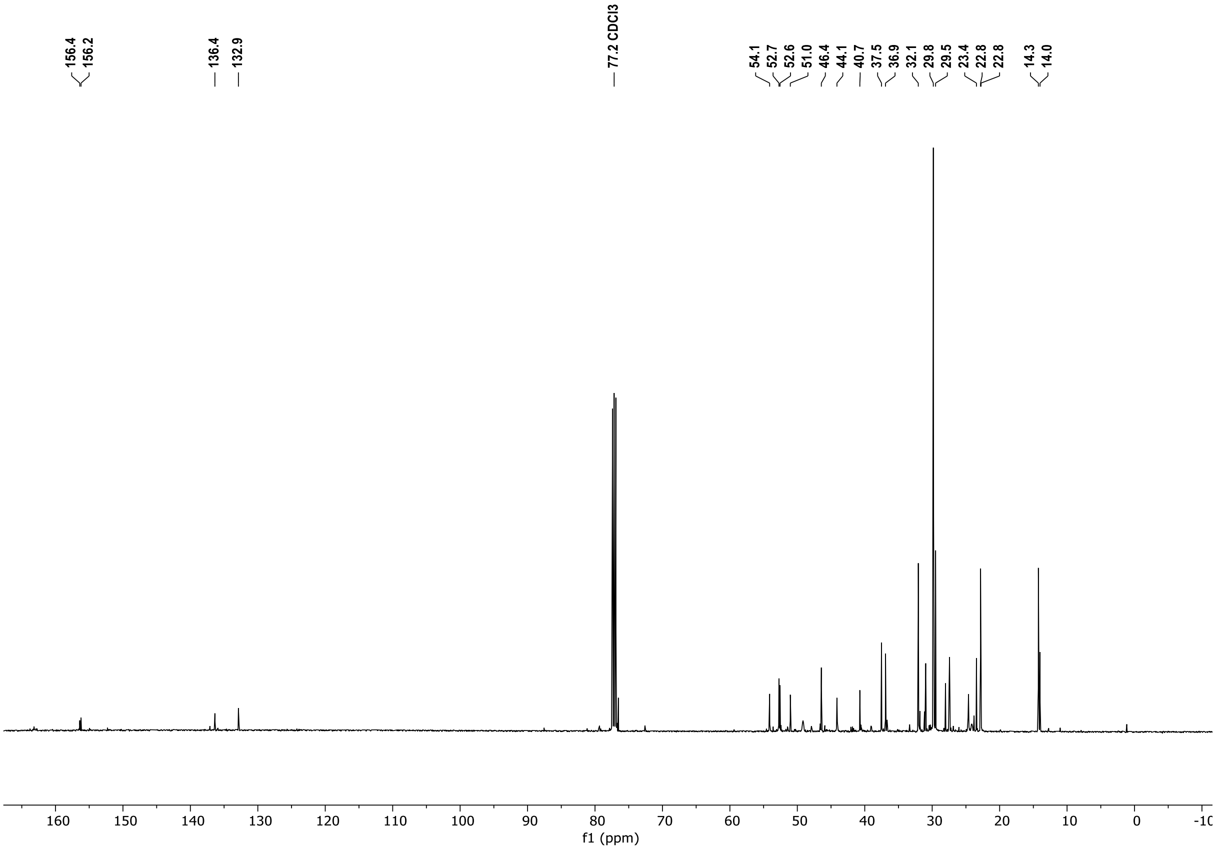

Supplement: Supplementary file 1 [file ol5c02963_si_001.pdf]
